# Inferring neural activity before plasticity as a foundation for learning beyond backpropagation

**DOI:** 10.1038/s41593-023-01514-1

**Published:** 2024-01-03

**Authors:** Yuhang Song, Beren Millidge, Tommaso Salvatori, Thomas Lukasiewicz, Zhenghua Xu, Rafal Bogacz

**Affiliations:** 1 Department of Computer Science, University of Oxford, Oxford, UK; 2 Medical Research Council Brain Network Dynamics Unit, University of Oxford, Oxford, UK; 3 Fractile, Ltd., London, UK; 4 Institute of Logic and Computation, Vienna University of Technology, Vienna, Austria; 5 VERSES AI Research Lab, Los Angeles, CA, USA; 6 State Key Laboratory of Reliability and Intelligence of Electrical Equipment, School of Health Sciences and Biomedical Engineering, Hebei University of Technology, Tianjin, China

## Abstract

For both humans and machines, the essence of learning is to pinpoint which components in its information processing pipeline are responsible for an error in its output, a challenge that is known as ‘credit assignment’. It has long been assumed that credit assignment is best solved by backpropagation, which is also the foundation of modern machine learning. Here, we set out a fundamentally different principle on credit assignment called ‘prospective configuration’. In prospective configuration, the network first infers the pattern of neural activity that should result from learning, and then the synaptic weights are modified to consolidate the change in neural activity. We demonstrate that this distinct mechanism, in contrast to backpropagation, (1) underlies learning in a well-established family of models of cortical circuits, (2) enables learning that is more efficient and effective in many contexts faced by biological organisms and (3) reproduces surprising patterns of neural activity and behavior observed in diverse human and rat learning experiments.

The credit assignment problem^1^ lies at the very heart of learning. Backpropagation^2^, as a simple yet effective credit assignment theory, has powered notable advances in artificial intelligence since its inception^3–5^ and has also gained a predominant place in understanding learning in the brain^1,6–8^. Due to this success, much recent work has focused on understanding how biological neural networks could learn in a way similar to backpropagation^9–12^; although many proposed models do not implement backpropagation exactly, they nevertheless try to approximate backpropagation, and much emphasis is placed on how close this approximation is^9,11,13,14^. However, learning in the brain is superior to backpropagation in many critical aspects. For example, compared to the brain, backpropagation requires many more exposures to a stimulus to learn^15^ and suffers from catastrophic interference of newly and previously stored information^16^. This raises the question of whether using backpropagation to understand learning in the brain should be the main focus of the field.

Here, we propose that the brain instead solves credit assignment with a fundamentally different principle, which we call ‘prospective configuration’. In prospective configuration, before synaptic weights are modified, neural activity changes across the network so that output neurons better predict the target output; only then are the synaptic weights (hereafter termed ‘weights’) modified to consolidate this change in neural activity. By contrast, in backpropagation, the order is reversed; weight modification takes the lead, and the change in neural activity is the result that follows.

We identify prospective configuration as a principle that is implicitly followed by a well-established family of neural models with solid biological groundings, namely, energy-based networks. These networks include Hopfield networks^17^ and predictive coding networks^18^, which have been successfully used to describe information processing in the cortex^19^. To support the theory of prospective configuration, we show that it can both yield efficient learning, which humans and animals are capable of, and reproduce data from experiments on human and animal learning. Thus, on the one hand, we demonstrate that prospective configuration performs more efficient and effective learning than backpropagation in various situations faced by biological systems, such as learning with deep structures, online learning, learning with a limited amount of training examples, learning in changing environments, continual learning with multiple tasks and reinforcement learning. On the other hand, we demonstrate that patterns of neural activity and behavior in diverse human and animal learning experiments, including sensorimotor learning, fear conditioning and reinforcement learning, can be naturally explained by prospective configuration but not by backpropagation.

Guided by the belief that backpropagation is the foundation of biological learning, previous work showed that energy-based networks can closely approximate backpropagation. However, to achieve it, the networks were set up in an unnatural way, such that the neural activity was prevented from substantially changing before weight modification by constraining the supervision signal to be infinitely small (for example, as in equilibrium propagation^11^ and in previous studies using predictive coding networks^12,20^) or last an infinitely short time^14,21^. By contrast, we reveal that energy-based networks without these unrealistic constraints follow the distinct principle of prospective configuration rather than backpropagation and are superior in both learning efficiency and accounting for data on biological learning.

Here, we introduce prospective configuration with an intuitive example, show how it originates from energy-based networks and describe its advantages and quantify them in a rich set of biologically relevant learning tasks. We show that prospective configuration naturally explains patterns of neural activity and behavior in diverse learning experiments.

## Results

### Prospective configuration: an intuitive example

To optimally plan behavior, it is critical for the brain to predict future stimuli, for example, to predict sensations in some modalities on the basis of other modalities^22^. If the observed outcome differs from the prediction, the weights in the whole network need to be updated so that predictions in the ‘output’ neurons are corrected. Backpropagation computes how the weights should be modified to minimize the error on the output, and this weight update results in a change in neural activity when the network next makes the prediction. By contrast, we propose that neural activity is first adjusted to a new configuration so that the output neurons better predict the observed outcome (target pattern); the weights are then modified to reinforce this configuration of neural activity. We call this configuration of neural activity ‘prospective’ because it is the neural activity that the network should produce to correctly predict the observed outcome. In agreement with the proposed mechanism of prospective configuration, it has indeed been widely observed in biological neurons that presenting the outcome of a prediction triggers changes in neural activity; for example, in tasks requiring animals to predict a juice delivery, the reward triggers rapid changes in activity not only in the gustatory cortex but also in multiple cortical regions^23,24^.

**Fig. 1:**
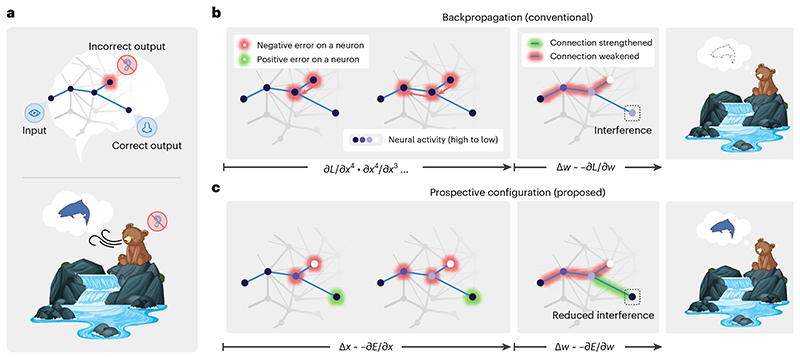
Prospective configuration avoids interference during learning. **a**, Abstract (top) and concrete (bottom) examples of a task inducing interference during learning. One stimulus input (seeing the water) triggers two prediction outputs (hearing the water and smelling the salmon). One output is correct (smelling the salmon), whereas the other output is an error (not hearing the water). **b**,**c**, Backpropagation produces interference during learning; not hearing the water reduces the expectation of smelling the salmon (**b**), although the salmon was indeed smelled. Prospective configuration, on the other hand, avoids such interference (**c**). In backpropagation, negative error propagates from the error output to hidden neurons (**b**; left). This causes a weakening of some connections, which, on the next trial, improves the incorrect output but also reduces the prediction of the correct output, thus introducing interference (**b**; middle and right). In prospective configuration, neural activity settles into a new configuration (different intensities of purple) before weight modification (**c**; left). This configuration corresponds to the activity that should be produced after learning, that is, is ‘prospective’. Hence, it foresees the positive error on the correct output and modifies the connections to improve the incorrect output while maintaining the correct output (**c**; middle and right).

To highlight the difference between backpropagation and prospective configuration, consider a simple example (Fig. 1a). Imagine a bear seeing a river. In the bear’s mind, the sight generates predictions of hearing water and smelling salmon. On that day, the bear indeed smelled the salmon but did not hear the water, perhaps due to an ear injury, and thus the bear needs to change its expectation related to the sound. Backpropagation (Fig. 1b) would proceed by backpropagating the negative error to reduce the weights on the path between the visual and auditory neurons. However, this also entails a reduction of the weights between visual and olfactory neurons that would compromise the expectation of smelling the salmon the next time the river is visited, even though the smell of salmon was present and correctly predicted. These undesired and unrealistic side effects of learning with backpropagation are closely related with the phenomenon of catastrophic interference, where learning a new association destroys previously learned memories^16^. This example shows that, with backpropagation, even learning one new aspect of an association may interfere with the memory of other aspects of the same association.

By contrast, prospective configuration assumes that learning starts with the neurons being configured to a new state, which corresponds to a pattern enabling the network to correctly predict the observed outcome. The weights are then modified to consolidate this state. This behavior can ‘foresee’ side effects of potential weight modifications and compensate for them dynamically (Fig. 1c). To correct the negative error on the incorrect output, the hidden neurons settle to their prospective state of lower activity, and, as a result, a positive error is revealed and allocated to the correct output. Consequently, prospective configuration increases the weights connecting to the correct output, whereas backpropagation does not (Fig. 1b,c). Hence, prospective configuration is able to correct the side effects of learning an association effectively and efficiently and with little interference.

### Origin of prospective configuration: energy-based networks

To show how prospective configuration naturally arises in energy-based networks, we introduce a physical machine analog, which provides an intuitive understanding of energy-based networks and how they produce the mechanism of prospective configuration.

Energy-based networks have been widely and successfully used in describing biological neural systems^17,25^. In these models, a neural circuit is described by a dynamical system driven by reducing an abstract ‘energy’, for example, reflecting errors made by neurons (Methods). Neural activity and weights change to reduce this energy; hence, they can be considered ‘movable parts’ of the dynamical system. We show that energy-based networks are mathematically equivalent to a physical machine (we call it ‘energy machine’), where the energy function has an intuitive interpretation, and its dynamics are straightforward; the energy machine simply adjusts its movable parts to reduce energy.

**Fig. 2:**
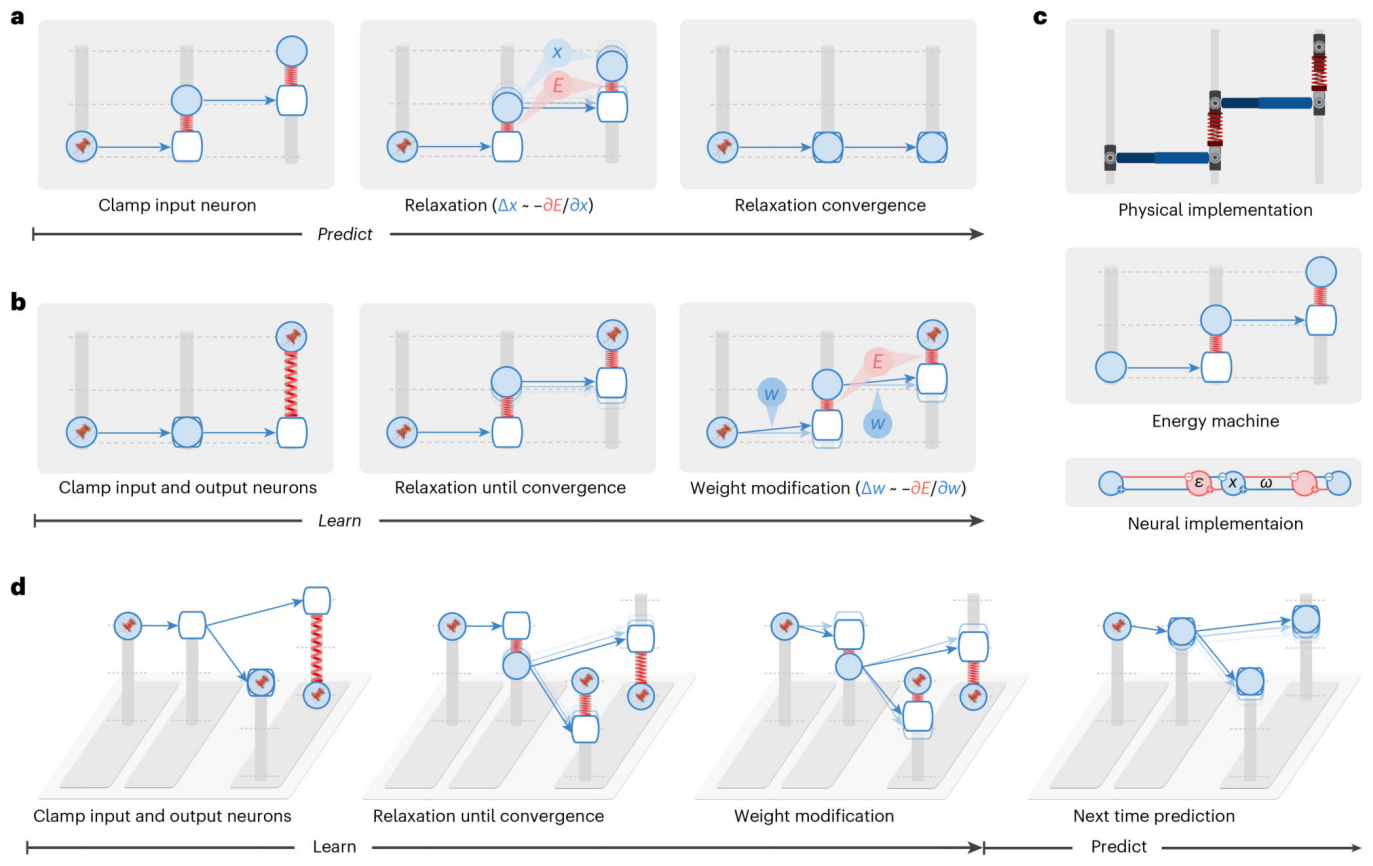
The energy machine reveals a new understanding of energy-based networks, the mechanism of prospective configuration and its theoretical advantages. A subset of energy-based networks can be visualized as mechanical machines that perform equivalent computations. Here, we present the energy machine corresponding to predictive coding networks^12,18^. In the energy machine, the activity of a neuron corresponds to the height of a node (represented by a solid circle) sliding on a post. The input to the neuron is represented by a hollow node on the same post. A synaptic connection corresponds to a rod pointing from a solid node to a hollow node. The weight determines how the input to a postsynaptic neuron depends on the activity of a presynaptic neuron; hence, it influences the angle of the rod. In energy-based networks, relaxation (that is, neural dynamics) and weight modification (that is, weight dynamics) are both driven by minimizing the energy, which corresponds to relaxation of the energy machine by moving the nodes and tuning the rods, respectively. **a**,**b**, Predictions (**a**) and learning (**b**) in energy-based networks visualized by the energy machine. The pin indicates that neural activity is fixed to the input or target pattern. Here, it is revealed that relaxation infers prospective neural activity, toward which the weights are then modified, a mechanism that we call prospective configuration. **c**, Physical implementation (top) and connectivity of a predictive coding network^12,18^ (bottom), which has dynamics mathematically equivalent to those of the energy machine in the middle (see Methods for details). **d**, The learning problem in Fig. 1 visualized by the energy machine, which learns to improve the incorrect output while not interfering with the correct output, thanks to the mechanism of prospective configuration.

The energy machine includes nodes sliding on vertical posts connected with each other via rods and springs (Fig. 2a,b). Translating from energy-based networks to the energy machine, neural activity maps to the vertical position of a solid node, a connection maps to a rod (blue arrow) pointing from one node to another (where the weight determines how the end position of the rod relates to the initial position), and the energy function maps to the elastic potential energy of springs with nodes attached on both ends (the natural length of the springs is 0). Different energy functions and network structures result in different energy-based networks, corresponding to energy machines with different configurations and combinations of nodes, rods and springs. In Fig. 2, we present the energy machine of predictive coding networks^12,18^ because they are most accessible and are established to be closely related to backpropagation^12,14^.

The dynamics of energy-based networks, which are driven by minimizing the energy function, map to relaxation of the energy machine, which is driven by reducing the total elastic potential energy on the springs. A prediction with energy-based networks involves clamping the input neurons to the provided stimulus and updating the activity of the other neurons, which corresponds to fixing one side of the energy machine and letting the energy machine relax by moving nodes (Fig. 2a). Learning with energy-based networks involves clamping the input and output neurons to the corresponding stimulus, first letting the activities of the remaining neurons converge and then updating weights, which corresponds to fixing both sides of the energy machine and letting the energy machine relax first by moving nodes and then tuning rods (Fig. 2b).

The energy machine reveals the essence of energy-based networks; relaxation before weight modification lets the network settle to a new configuration of neural activity corresponding to the neural activity that would have occurred after the error was corrected by the modification of weights, that is, prospective activity (thus, we call this mechanism prospective configuration). For example, the second-layer ‘neuron’ in Fig. 2b increases its activity, and this increase in activity would also be caused by the subsequent weight modification (of the connection between the first and second neurons). In simple terms, relaxation in energy-based networks infers the prospective neural activity after learning, toward which the weights are then modified. This distinguishes it from backpropagation, where weight modification takes the lead, and the change in neural activity is the result that follows.

The bottom of Fig. 2c shows the connectivity of a predictive coding network^12,18^, which has dynamics mathematically equivalent to those of the energy machine shown above it. Predictive coding networks include neurons (blue) corresponding to nodes on the posts and separate neurons encoding prediction errors (red) corresponding to springs. For details, see Methods and Supplementary Fig. 1, where we list equations describing predictive coding networks and show how they map on the neural implementation and the proposed energy machine.

Using the energy machine, Fig. 2d simulates the learning problem from Fig. 1. Here, we can see that prospective configuration indeed foresees the result of learning and its side effects through relaxation. Hence, it corrects the side effects within one iteration, which would otherwise take multiple iterations for backpropagation.

### Advantages of prospective configuration: reduced interference and faster learning

Here, we quantify interference in the above scenario and demonstrate how reduced interference translates into an advantage in performance. In all simulations in the main text, prospective configuration is implemented in predictive coding networks (other energy-based models are considered in the Supplementary Notes, Section 2.1). We also compare the performance of predictive coding networks against artificial neural networks (ANNs) trained with backpropagation because they are closely related, which makes the comparisons fair. In particular, although predictive coding networks include recurrent connections, they generate the same prediction for a given input (when inputs are constrained but outputs are not; Fig. 2a) as standard feedforward ANNs if their weights are set to corresponding values^12,14^. Therefore, loss is the same function of weights in both models, so direct minimization of loss with gradient descent in predictive coding networks (which is not their natural way of training) would produce the same weight changes as backpropagation in ANNs. Hence, comparing predictive coding networks and backpropagation enables isolation of the effects of the learning algorithm (prospective configuration versus direct minimization of loss as in backpropagation).

**Fig. 3:**
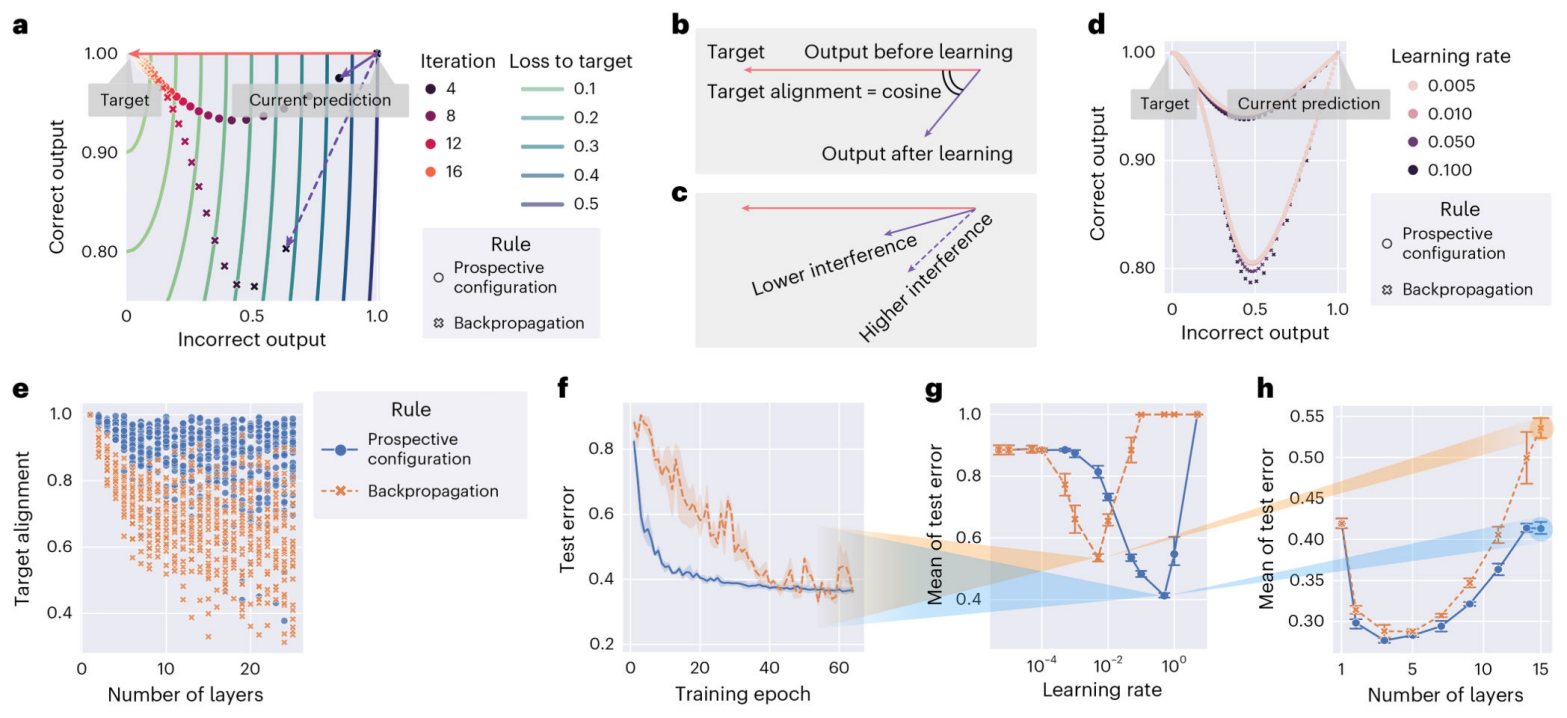
Learning with prospective configuration changes the activity of output neurons in a direction more aligned toward the target. **a**, Simulation of the network from Fig. 1 showing changes in the correct and incorrect output neurons during training (‘Iteration’) trained with both learning rules. Here, learning with prospective configuration (purple solid vector) aligns better with the target (red vector) than learning with backpropagation (purple dashed vector). **b**, Interference can be quantified by ‘target alignment’, the cosine similarity of the direction of the target (red vector) and the direction of learning (purple vector). **c**, Higher target alignment indicates less interference and vice versa. **d**, The same experiment as in **a** repeated with a learning rate ranging from 0.005 to 0.5 represented by the size of the markers, where it is shown that the choice of learning rate changes the trajectories for both methods slightly, but the conclusion holds irrespective of the learning rate. **e**, Target alignment of randomly generated networks trained with both learning rules as a function of depth of the network. Each symbol shows target alignment resulting from training on a single randomly generated pattern. **f**, Test error during training on the FashionMNIST^60^ dataset containing images of clothing belonging to different categories for both learning rules with a deep neural network of 15 layers. Here, ‘test error’ refers to the ratio of incorrectly classified samples among all samples in the test set. **g**, Mean of the test error over training epochs (reflecting how fast test error drops) as a function of learning rate. Results in **f** and **h** are for the learning rates giving the minima of the corresponding curves in **g**. **h**, Mean of test error of other network depths. Each point is from a learning rate independently optimized for each learning rule in the corresponding setup of network depth. In **e**–**h**, prospective configuration demonstrates a notable advantage as the structure gets deeper. Each experiment in **f**–**h** was repeated with *n* = 3 random seeds. Error bars and bands represent the 68% confidence interval.

In Fig. 3a, we compare the activity of output neurons in the example in Fig. 1 between backpropagation and prospective configuration. Initially both output neurons are active (top right), and the output should change toward a target in which one of the neurons is inactive (red vector). Learning with prospective configuration results in changes on the output (purple solid vector) that are aligned better with the target than those for backpropagation (purple dotted vector).

Following the first weight update, we simulate multiple iterations until the network is able to correctly predict the target. Here, ‘iteration’ refers to each time the agent is presented with stimuli and conducts one weight update because of the stimulus. Although the output from backpropagation can reach the target after multiple iterations, the output for the ‘correct neuron’ diverges from the target during learning and then comes back; this is a particularly undesired effect in biological learning, where networks can be ‘tested’ at any point during the learning process, because it may lead to incorrect decisions affecting chances for survival. By contrast, prospective configuration substantially reduces this effect.

Although backpropagation modifies weights to directly reduce cost in the space of weights (that is, performs gradient descent), surprisingly, and rather subversively, it does not push the resulting output activity directly toward the target. To illustrate this, Fig. 3a visualizes the cost with contour lines. Changing the activity of output neurons according to the gradient of the cost would correspond to a change orthogonal to the contour lines, that is, that indicated by the red arrow. However, backpropagation changes the output in a different direction shown by a dashed arrow. Optimizing the weights independently, without considering the effect of updating other weights, leads to output activity not updating toward the target directly due to different weight updates to different layers interfering with each other. By contrast, prospective configuration considers the results of updating other weights by finding a desired configuration of neural activity first. Such a mechanism is missing in backpropagation but is natural in energy-based networks. Supplementary Fig. 2 shows a direct comparison of how these two models evolve in weight and output spaces during learning.

Interference can be quantified by the angle between the direction of the target (from current output to target) and learning (from current output to output after learning, both measured without the target provided), and we define ‘target alignment’ as the cosine of this angle (Fig. 3b); hence, high interference corresponds to low target alignment (Fig. 3c).

It is useful to highlight that target alignment is affected little by the learning rate (Fig. 3d), demonstrating that the learning rate has little effect on the direction and trajectory that output neurons take. The difference in target alignment demonstrated in Fig. 3a is also present for deeper and larger (randomly generated) networks (Fig. 3e). When a network has no hidden layers, the target alignment is equal to 1 (Supplementary Notes, Section 2.4.1). The target alignment drops for backpropagation as the network gets deeper because changes in weights in one layer interfere with changes in other layers (Fig. 1), and the backpropagated errors do not lead to appropriate modification of weights in hidden layers (Supplementary Fig. 2). Because back-propagation modifies the weights in the direction reducing loss, it has positive target alignment for small learning rates but not necessarily close to 1. By contrast, prospective configuration maintains a much higher value along the way. This higher target alignment of prospective configuration can be theoretically explained by the following: (1) there exists a close link between prospective configuration and an algorithm called target propagation^26^ (shown in Supplementary Fig. 3 and Supplementary Notes, Section 2.2), and (2) under certain conditions, target propagation^26^ has a target alignment of 1 (ref. 27; demonstrated in Supplementary Fig. 4 and Supplementary Notes, Section 2.4.2). Thus, the link with target propagation provides theoretical insight (with numerical verification) into why prospective configuration has a higher target alignment.

Higher target alignment directly translates to the efficiency of learning. Test error during training in a visual classification task with a deep neural network of 15 layers decreases faster for prospective configuration than for backpropagation (Fig. 3f).

Throughout the data presented here, if learning rate is not presented in a plot, the plot corresponds to the best learning rate optimized independently for each rule under the setup via a grid search. The optimization target is either learning performance or similarity to experimental data (details can be found in the methods for each experiment). Thus, for example, Fig. 3f shows the test errors as training progress, with the learning rates optimized independently for each learning rule. The optimization target is the ‘mean of test error’ during training, reflecting how fast the test error decreases during training. Fig. 3g plots this mean of test error for different learning rates for both learning rules, and the learning rates giving the minima of the curves were used in Fig. 3f. Fig. 3h repeats the experiment on networks of other depths and shows the mean of the test error during training as a function of network depth. The mean error is higher for lower depths, as these networks are unable to learn the task, and for greater depths, as it takes longer to train deeper networks. Importantly, the gap between backpropagation and prospective configuration widens for deeper networks, paralleling the difference in target alignment. Efficient training with deeper networks is important for biological neural systems known to be deep, for example, the primate visual cortex^28^.

In Section 2.3 of the Supplementary Notes, we develop a formal theory of prospective configuration and provide further illustrations and analyses of its advantages. Supplementary Fig. 5 formally defines prospective configuration and demonstrates that it is indeed commonly observed in different energy-based networks. Supplementary Figs. 6 and 7 empirically verify and generalize the advantages expected from the theory and show that prospective configuration yields more accurate error allocation and less erratic weight modification, respectively.

### Advantages of prospective configuration: effective learning in biologically relevant scenarios

Inspired by these advantages, we show empirically that prospective configuration indeed handles various learning problems that biological systems would face better than backpropagation. Because the field of machine learning has developed effective benchmarks for testing learning performance, we use variants of classic machine learning problems that share key features with learning in natural environments. Such problems include online learning, where weights must be updated after each experience (rather than a batch of training examples)^29^, continual learning with multiple tasks^30^, learning in changing environments^31^, learning with a limited amount of training examples and reinforcement learning^4^. In all aforementioned learning problems, prospective configuration demonstrates a notable superiority over backpropagation.

**Fig. 4:**
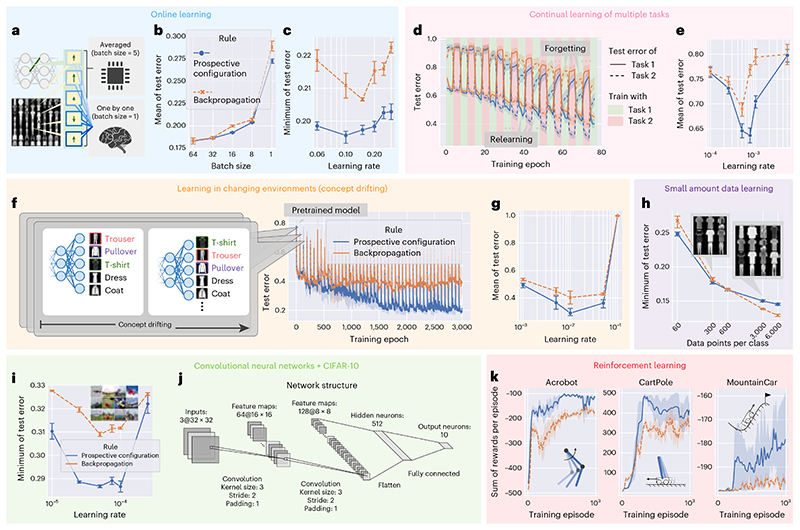
Prospective configuration achieves a superior performance over backpropagation in various learning situations faced by biological systems. **a**–**k**, Learning situations include online learning^29^ (**a**–**c**), continual learning of multiple tasks^30^ (**d**–**e**), learning in changing environments^31^ (**f**–**g**), learning with a limited amount of training examples (**h**) and reinforcement learning^4^ (**k**). Graphs corresponding to each situation are grouped together with the same background color. Simulations of each situation differ from the ‘default setup’ described in the Methods in a single aspect unique to this task. For example, the default setup involves training with minibatches, so the batch size was only set to 1 in **a**–**c** for investigating online learning, whereas it was set to a larger default value in rest of the groups. In supervised learning setups, fully connected networks (**a**–**h**) were evaluated on the FashionMNIST^60^ dataset, and convolutional neural networks^35^ (**i** and **j**) were evaluated on the CIFAR-10 (ref. 36) dataset. In the reinforcement learning setup (**k**), fully connected networks were evaluated on three classic control problems. If the learning rate was not presented, each point (a setup of an experiment) in the plot corresponds to the best learning rate optimized independently for each rule under that setup. **a**, Difference in training setup between computers that can average weight modifications for individual examples to get a ‘statistically good’ value and biological systems that must apply one modification before computing another. **b**, Mean of the test errors during training as a function of batch size. **c**, Minimum of test error during training as a function of learning rate. **d**, Test error during continual learning of two tasks. **e**, Mean of test error of both tasks during training as a function of learning rate. **f**, Test error during training when learning with concept drifting. **g**, Mean of test error during training with concept drifting as a function of learning rate. **h**, Minimum of test error during training with different amounts of training examples (data points per class). **i**, Minimum of test error during training of a convolutional neural network trained with prospective configuration and backpropagation on the CIFAR-10 (ref. 36) dataset. **j**, Structure detail of the convolutional neural network used in **i**. **k**, Sum of rewards per episode during training on three classic reinforcement learning tasks (insets). An episode is a period from initialization of environment to reaching a terminate state. Each experiment in **a**–**h** was repeated with *n* = 10 random seeds. Each experiment in **i**–**k** was repeated with *n* = 3 random seeds because these experiments are more expensive. Error bars and bands represent the 68% confidence interval.

First, based on the example in Fig. 1, we expect prospective configuration to require fewer episodes for learning than backpropagation. Before presenting the comparison, we describe how backpropagation is used to train ANNs. Typically, the weights are only modified after a batch of training examples based on the average of updates derived from individual examples (Fig. 4a). In fact, backpropagation relies heavily on averaging over multiple experiences to reach human-level performance^32^, as it needs to stabilize training^33^. By contrast, biological systems must update the weights after each experience, and we compare learning performance in such a setting. Sampling efficiency can be quantified by mean of test error during training, which is shown in Fig. 4b as a function of batch size (number of experiences that the updates are averaged over). Efficiency strongly depends on batch size for backpropagation because it requires batch training to average out erratic weight updates, whereas this dependence is weaker for prospective configuration, where weight changes are intrinsically less erratic and batch averaging is required less (Supplementary Fig. 7). Importantly, prospective configuration learns faster with smaller batch sizes, as in biological settings. Additionally, final performance can be quantified by the minimum of the test error, which is shown in Fig. 4c, when trained with a batch size equal to 1. Here, prospective configuration also demonstrates a notable advantage over backpropagation.

Second, biological organisms need to sequentially learn multiple tasks, while ANNs show catastrophic forgetting. When trained on a new task, performance on previously learned tasks is largely destroyed^16,34^. The data in Fig. 4d show performance when trained on two tasks alternately (task 1 is classifying five randomly selected classes in the FashionMNIST dataset, and task 2 is classifying the remaining five classes). Prospective configuration outperforms backpropagation both in terms of avoiding forgetting previous tasks and relearning current tasks. The results are summarized in Fig. 4e.

Third, biological systems often need to rapidly adapt to changing environments. A common way to simulate this is ‘concept drifting’^31^, where a part of the mapping between the output neurons to the semantic meaning is shuffled regularly, each time a certain number of training iterations has passed (Fig. 4f). Test error during training with concept drifting is presented in Fig. 4f. Before epoch 0, both learning rules are initialized with the same pretrained model (trained with backpropagation); thus, epoch 0 is the first time the model experiences concept drift. The results are summarized in Fig. 4g and show that, for this task, there is a particularly large difference in mean error (for optimal learning rates). This large advantage of prospective configuration is related to it being able to optimally detect which weights to modify (Supplementary Fig. 6) and to preserve existing knowledge while adapting to changes (Fig. 1). This ability to maintain important information while updating other information is critical for survival in natural environments that are bound to change, and prospective configuration has a very substantial advantage in this respect.

Furthermore, biological learning is also characterized by limited data availability. Prospective configuration outperforms backpropagation when the model is trained with fewer examples (Fig. 4h).

To demonstrate that the advantage of prospective configuration also scales up to larger networks and problems, we evaluated convolutional neural networks^35^ on CIFAR-10 (ref. 36) trained with both learning rules (Fig. 4i), where prospective configuration showed notable advantages over backpropagation. The detailed structure of the convolutional networks is provided in Fig. 4j.

Another key challenge for biological systems is to decide which actions to take. Reinforcement learning theories (for example, *Q* learning) propose that it is solved by learning the expected reward resulting from different actions in different situations^37^. Such prediction of rewards can be made by neural networks^4^, which can be trained with prospective configuration or backpropagation. The sum of rewards per episode during training on three classic reinforcement learning tasks is reported in Fig. 4k, where prospective configuration demonstrates a notable advantage over backpropagation. This large advantage may arise because reinforcement learning is particularly sensitive to erratic changes in network weights (as the target output depends on reward predicted by the network itself for a new state; Methods).

Based on the superior learning performance of prospective configuration, we may expect that this learning mechanism has been favored by evolution; thus, in the next sections, we investigate if it can account for neural activity and behavior during learning better than backpropagation.

### Evidence for prospective configuration: inferring the latent state during learning

Prospective configuration is related to theories proposing that before learning, the brain first infers a latent state of the environment from feedback^38–40^. Here, we propose that this inference can be achieved in neural circuits through prospective configuration, where, following feedback, neurons in ‘hidden layers’ converge to a prospective pattern of activity that encodes this latent state. We demonstrate that data from various previous studies, which involved the inference of a latent state, can be explained by prospective configuration. These data were previously explained by complex and abstract mechanisms, such as Bayesian models^38,39^, whereas here, we mechanistically show with prospective configuration how such inference can be performed by minimal networks encoding only the essential elements of the tasks.

The dynamical inference of a latent state from feedback has been recently proposed to take place during sensorimotor learning^39^. In this experiment, participants received different motor perturbations in different contexts and learned to compensate for these perturbations. Behavioral data suggest that, after receiving feedback, participants first used the feedback to infer context and then adapted the force for the inferred context. We demonstrate that prospective configuration is able to reproduce these behavioral data, whereas backpropagation cannot.

**Fig. 5:**
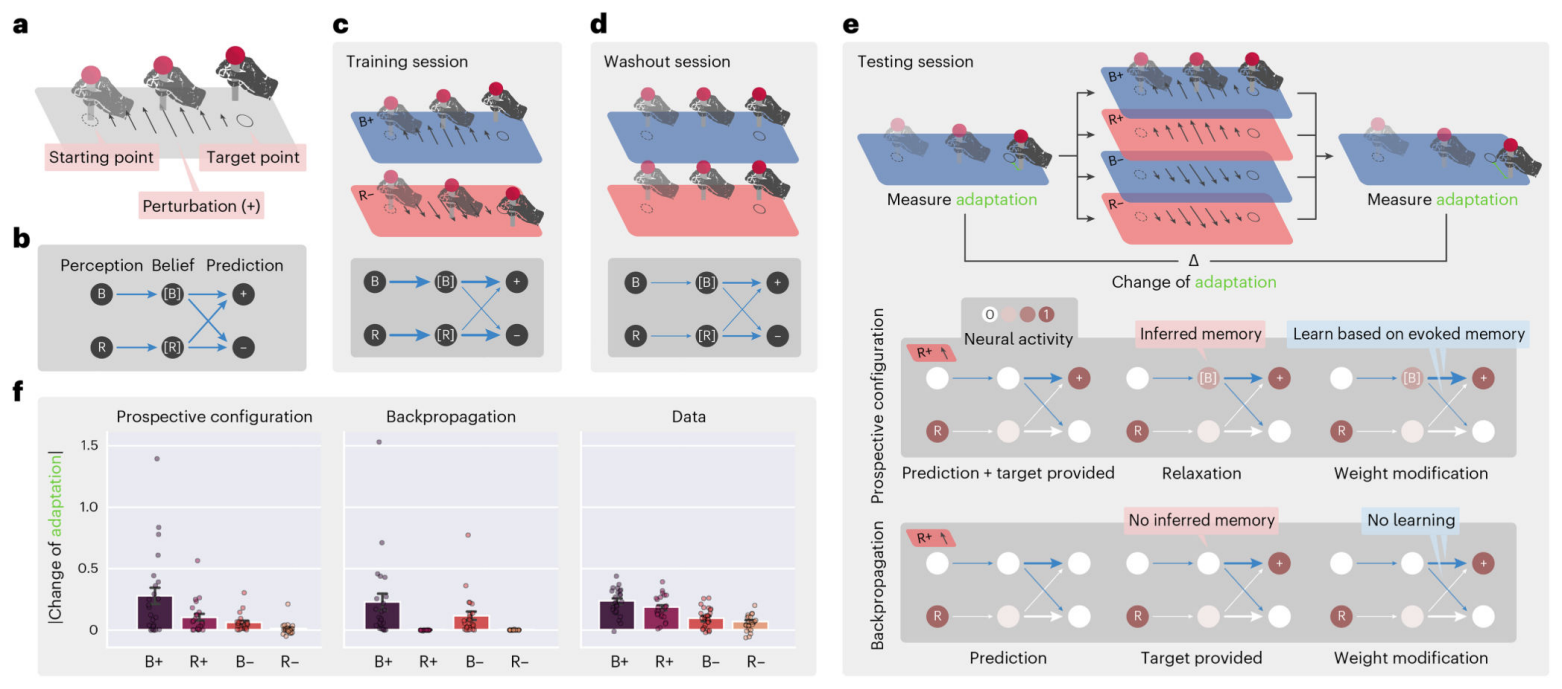
Prospective configuration explains contextual inference in human sensorimotor learning. **a**, Structure of an experimental trial where participants were asked to move a stick from the starting point to the target point while experiencing perturbations. **b**, The minimal network for the task, including six connections encoding the associations from the backgrounds (B and R) to the belief of contexts ([B] and [R]) and from the belief of contexts to the prediction of perturbations (+ and –). **c**–**e**, Sequence of sessions the participants experienced, including training (**c**), washout (**d**) and testing (**e**). Darker gray boxes show the expected network after the session, where thickness represents the strength of connections. In the testing session, the darker box explains how the two learning rules learn differently on the R+ trial, leading to the differences in **f**. **f**, Predictions of the two learning rules compared to behavioral data measured from human participants, where prospective configuration reproduces the key patterns of data, but backpropagation does not. Each experiment was repeated with *n* = 24 random seeds, as there were 24 participants in the behavioral experiment.

Specifically, in the task (Fig. 5a), participants were asked to move a stick from a starting point to a target point while experiencing perturbations. The participants experienced a sequence of blocks of trials (Fig. 5c–e), including training, washout and testing. During the training session, different directions of perturbations, positive (+) or negative (–), were applied in different contexts, blue (B) or red (R) backgrounds, respectively. We denote these trials as B+ and R–. These trials may be associated with latent states, which we denote [B] and [R]; for example, the latent state [B] may be associated with both background B and perturbation +. The next stage of the task was designed to investigate if the latent state [B] can be activated by perturbation + even if no background B is shown. Thus, participants experienced different trials including R+ (that is, perturbation + but no background B). Specifically, after a washout session (during which no perturbation was provided), in the testing session, participants experienced one of the four possible test trials: B+, R+, B– and R–. To evaluate learning on the test trials, motor adaptation (that is, the difference between the final and target stick positions) was measured before and after the test trial in two trials with the blue background (Fig. 5e). Change in the adaptation between these two trials is a reflection of learning about blue context that occurred at the test trial. If participants only associated feedback with the background color (B), then the change in adaptation would only occur with test trials B+ and B–. However, experimental data (Fig. 5f) show that there was also substantial adaptation change with R+ trials (which was even bigger than with B– trials).

To model learning in this task, we considered a neural network (Fig. 5b) where input nodes encode the background color, and outputs encode movement compensations in the two directions. Importantly, this network also includes hidden neurons encoding belief of being in the contexts associated with the two backgrounds ([B] and [R]). Trained with the exact procedure of the experiment^39^ from randomly initialized weights, prospective configuration with this minimal network can reproduce the behavioral data, whereas backpropagation cannot (Fig. 5f).

Prospective configuration can produce change in adaptation with the R+ test trial because after + feedback, it is able to also activate context [B] that was associated with this feedback during training and then learn compensation for this latent state. To shed light on how this inference takes place in the model, schematics in Fig. 5c,d show evolution of the weights of the network over sessions (thickness represents the strength of connections). The schematic in Fig. 5e shows the difference between the two learning rules after exposure to R+; although B is not perceived, prospective configuration infers a moderate excitation of the belief of blue context [B] because the positive connection from [B] to + was built during the training session. The activity of [B] enables the learning of weights from [B] to + and –, while backpropagation does not modify any weights originating from [B].

For simplicity of explanation, we presented simulations with minimal networks; however, Supplementary Fig. 8 shows that networks with a general fully connected structure and more hidden neurons can replicate the above data when using prospective configuration but not when using backpropagation.

Studies of animal conditioning have also observed that feedback in learning tasks involving multiple stimuli may trigger learning about non-presented stimuli^41,42^. One example is provided in Supplementary Fig. 9, where we show that it can be explained by prospective configuration but not by backpropagation.

### Evidence for prospective configuration: discovering task structure during learning

**Fig. 6:**
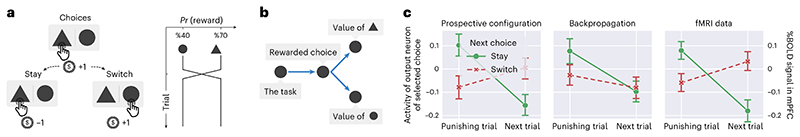
Prospective configuration can discover the underlying task structure during reinforcement learning. **a**, Reinforcement learning task. Human participants were required to choose between two options, leading to either reward (gaining coins) or punishment (losing coins) with different probabilities. The probability of reward was occasionally reversed between the two options. **b**, The minimal network encoding the essential elements of the task. **c**, Activity of the output neuron corresponding to the selected option from networks trained with prospective configuration and backpropagation compared with fMRI data measured in human participants (that is, peak blood oxygenation level-dependent (%BOLD) signal in the mPFC). Prospective configuration reproduces the key finding that the expected value (encoded in %BOLD signal in the mPFC) increases if the next choice after a punishing trial is to switch to the other option. The number of trials is not mentioned in the original paper, so we simulated for *n* = 128 trials for both learning rules. Error bars represent the 68% confidence interval.

Prospective configuration is also able to discover the underlying task structure in reinforcement learning. Specifically, we consider a task where reward probabilities of different options were not independent^38^. In this study, humans were choosing between two options where the reward probabilities were constrained such that one option had a higher reward probability than the other (Fig. 6a). Occasionally the reward probabilities were swapped, so if one probability was increased, the other was decreased by the same amount. Remarkably, the recorded functional magnetic resonance imaging (fMRI) data suggested that participants learned that the values of the two options were negatively correlated and on each trial updated the value estimates of both options in opposite ways. This conclusion was drawn from analysis of the signal from the medial prefrontal cortex (mPFC), which encoded the expected value of reward. The data presented in Fig. 6c compare this signal after making a choice on two consecutive trials: a trial in which the reward was not received (‘punish trial’) and the next trial. If the participant selected the same option on both trials (‘stay’), the signal decreased, indicating that the reward expected by the participant was reduced. Remarkably, if the participant selected the other option on the next trial (‘switch’), the signal increased, suggesting that negative feedback for one option increased the value estimate for the other. Such learning is not predicted by standard reinforcement learning models^38^.

This task can be conceptualized as having a latent state encoding which option is superior, and this latent state determines the reward probabilities for both options. Consequently, we consider a neural network reflecting this structure (Fig. 6b) that includes an input neuron encoding being in the task (equal to 1 in simulations), a hidden neuron encoding the latent state and two output neurons encoding the reward probabilities for the two options. Trained with the exact procedure of the experiment^38^ from randomly initialized weights, prospective configuration with this minimal network can reproduce the data, whereas backpropagation cannot (Fig. 6c). In Supplementary Fig. 10, we show that prospective configuration reproduces these data because it can infer the rewarded choice by updating the activity of the hidden neuron based on feedback.

Taken together, the presented simulations illustrate that prospective configuration is a common principle that can explain a range of surprising learning effects in diverse tasks.

## Discussion

Our paper identifies the principle of prospective configuration, according to which learning relies on neurons first optimizing their pattern of activity to match the correct output and then reinforcing these prospective activities through synaptic plasticity. Although it was known that in energy-based networks the activity of neurons shifts before weight update, it has been previously thought that this shift is a necessary cost of error propagation in biological networks, and several methods have been proposed to suppress it^11,12,14,20,21^ to approximate backpropagation more closely. By contrast, we demonstrate that this reconfiguration of neural activity is the key to achieving learning performance superior to that of backpropagation and to explaining experimental data from diverse learning tasks. Prospective configuration further offers a range of experimental predictions distinct from those of backpropagation (Supplementary Figs. 11 and 12). Together, we have demonstrated that prospective configuration enables more efficient learning than backpropagation by reducing interference, demonstrates superior performance in situations faced by biological organisms, requires only local computation and plasticity and matches experimental data across a wide range of tasks.

Our theory addresses a long-standing question of how the brain solves the plasticity-stability dilemma, for example, how it is possible that, despite adjustment of representation in the primary visual cortex during learning^43^, we can still understand the meaning of visual stimuli we learned over our lifetime. According to prospective configuration, when some weights are modified, compensatory changes are made to other weights to ensure the stability of correctly predicted outputs. Thus, prospective configuration reduces interference between different weight modifications while learning a single association. Previous computational models have proposed mechanisms that reduce interference between new and previously acquired information while learning multiple associations^34,44^. It is highly likely that such mechanisms and prospective configuration operate in the brain in parallel to minimize both types of interference.

Prospective configuration is related to inference and learning procedures in statistical modeling. If the ‘energy’ in energy-based schemes is variational free energy, prospective configuration can be seen as an implementation of variational Bayes that subsumes inference and learning^45^. For example, dynamic expectation maximization^46,47^ can be regarded as a generalization of predictive coding networks in which the D-step optimizes representations of latent states (analogously to relaxation until convergence during inference) while the E-step optimizes model parameters (analogously to weight modification during learning).

Other recent work^48,49^ also noticed that the natural form of energy-based networks (‘strong control’ in their words) performs different learning than backpropagation. Their analysis concentrates on an architecture of deep feedback control, and they demonstrated that a particular form of their model is equivalent to predictive coding networks^49^. The unique contribution of our paper is to show the benefits of such strong control and explain why they arise. The principle of prospective configuration is also present in other recent models. For example, Gilra and Gerstner^50^ developed a spiking model in which feedback about the error on the output directly affects the activity of hidden neurons before plasticity takes place. Haider et al.^51^ developed a faster inference algorithm for energy-based models that computes a value to which the activity is likely to converge, termed latent equilibrium^51^. Iteratively setting each neuron’s output based on its latent equilibrium leads to much faster inference^51^ and enables efficient computation of the prospective configuration.

Predictive coding networks require symmetric forward and backward weights between layers of neurons, so a question arises concerning how such symmetry may develop in the brain. If predictive coding networks are initialized with symmetric weights (as in our simulations), the symmetry will persist because the changes in weight between neurons A and B are the same as those for feedback weight (between neurons B and A). Even if the weights are not initialized symmetrically, the symmetry may develop if synaptic decay is included in the model^52^ because then the initial asymmetric values decay away, and weight values become more influenced by recent changes that are symmetric. Nevertheless, weight symmetry is not generally required for effective credit assignment^53,54^.

Here, we assumed for simplicity that the convergence of neural activity to an equilibrium happens rapidly after the stimuli are provided so that the synaptic weight modification after convergence may take place while the stimuli are still present. Nevertheless, predictive coding networks can still work even if weight modification takes place while the neural activity is converging. Specifically, Song et al. demonstrated that if neural activities are only updated for the first few steps, the update of the weights is equivalent to that in backpropagation^14^. As a reminder, we demonstrate here that if the neural activities are updated to equilibrium, the update of the weights follows the principle of prospective configuration and possesses the desirable demonstrated properties. Thus, a learning rule where neural activities and weights are updated in parallel will experience a weight update that is equivalent to backpropagation at the start and then move to prospective configuration as the system converges to equilibrium^55^. Furthermore, predictive coding networks have been extended to describe recurrent structures^56–58^, and it has been shown that such networks can learn to predict dynamically changing stimuli even if weights are modified before the activity converged for a given ‘frame’ of the stimulus^57^.

The advantages of prospective configuration suggest that it may be profitably applied in machine learning to improve the efficiency and performance of deep neural networks. An obstacle for this is that the relaxation phase is computationally expensive. However, recent work demonstrated that by modifying weights after each step of relaxation, the model becomes comparably fast to backpropagation and easier for parallelization^55^.

Most intriguingly, it has been demonstrated that the speed of energy-based networks can be greatly increased by implementing the relaxation on analog hardware^59^, potentially resulting in energy-based networks being faster than backpropagation. Therefore, we anticipate that our discoveries may change the blueprint of next-generation machine learning hardware, switching from the current digital tensor base to analog hardware and being closer to the brain and potentially far more efficient.

## Methods

This section provides the necessary details for replication of the results described in the main text.

### Models

Throughout this work, we compare the established theory of backpropagation to the proposed new principle of prospective configuration. As explained in the main text, backpropagation is used to train ANNs, where the activity of a neuron is fixed to a value based on its input, whereas prospective configuration occurs in energy-based networks, where the activity of a neuron is not fixed.

Because in ANNs the activity of neurons ***x*** is determined by their input, the output of the network can be obtained by propagating the inputs ‘forward’ through the computational graph. The output can then be compared to a target pattern to get a measure of difference known as a loss. Because the value of a node (activity of a neuron) in the computational graph is explicitly computed as a function of its input, the computational graph is usually differentiable. Thus, training ANNs with backpropagation modifies the weights ***w*** to take a step toward the negative gradient of loss L,

(1) $$\text{Δ}w=-\alpha \frac{\partial L}{\partial w},$$

during which the activities of neurons ***x*** are fixed, and *α* is the learning rate. The weights ***w*** requiring modification might be many steps away from the output on the computational graph, where the loss L is computed; thus, $\frac{\partial L}{\partial w}$ is often obtained by applying the chain rule of computing a derivative through intermediate variables (activity of output and hidden neurons). For example, consider a network with four layers, and let ***x**^l^* denote the activity of neurons in layer *l* and ***w**^l^* denote the weights of connections between layers *l* and *l* + 1. The change in weights originating from the first layer is then computed: $\frac{\partial L}{\partial w^{1}}=\frac{\partial L}{\partial x^{4}}\cdot \frac{\partial x^{4}}{\partial x^{3}}\ldots \frac{\partial x^{2}}{\partial w^{1}}$. This enables the loss to be backpropagated through the graph to provide a direction of update for all weights.

In contrast to ANNs, in energy-based networks, the activity of neurons ***x*** is not fixed to the input from a previous layer. Instead, an energy function *E* is defined as a function of the neural activity ***x*** and weights ***w***. For networks organized in layers (considered in this paper), the energy can be decomposed into a sum of local energy terms *E^l^*,

(2) $$E=\sum_{l}E^{l}\left(x^{l},w^{l-1},x^{l-1}\right).$$

Here, *E^l^* is called local energy because it is a function of ***x**^l^*, ***x***^*l* − 1^ and ***w***^*l* − 1^, which are neighbors and connected to each other. This ensures that the optimization of energy *E* can be implemented by local circuits because the derivative of *E* with respect to any neural activity (or weights) results in an equation containing only the local activity (or weights) and the activity of adjacent neurons. Predictions with energy-based networks are computed by clamping the input neurons to an input pattern and then modifying the activity of all other neurons to decrease the energy:

(3) $$\text{Δ}x=-\gamma \frac{\partial E}{\partial x},$$

where *γ* is the integration step of the neural dynamics. Because the terms in *E* can be divided into local energy terms, this results in an equation that can be implemented with local circuits. This process of modifying neural activity to decrease the energy is called relaxation, and we refer to the equation describing relaxation as neural dynamics because it describes the dynamics of the neural activity in energy-based networks. After convergence of relaxation, the activities of the output neurons are taken as the prediction made by the energy-based network. Different energy-based networks are trained in slightly different ways. For predictive coding networks^12,18^, training involves clamping the input and output neurons to input and target patterns, respectively. Then, relaxation is run until convergence $\left(x=\overset{*}{x}\right)$, after which the weights are updated using the activity at convergence to further decrease the energy:

(4) $$\text{Δ}w=-\alpha {\frac{\partial E}{\partial w}|}_{x=\overset{*}{x}}.$$

This will also result in an equation that can be implemented with local plasticity because it is just a gradient descent on the local energy. We refer to such an equation as weight dynamics, because it describes the dynamics of the weights in energy-based networks.

Backpropagation and prospective configuration are not restricted to specific models. Depending on the structure of the network and the choice of the energy function, one can define different models that implement the principle of backpropagation or prospective configuration. In the main text and most of the Supplementary Notes, we investigate the most standard layered network. In this case, both ANNs and energy-based networks include *L* layers of weights ***w***^1^, ***w***^2^, …, ***w**^L^* and *L* + 1 layers of neurons ***x***^1^, ***x***^2^, …, ***x***^*L* + 1^, where ***x***^1^ and ***x***^*L* + 1^ are the input and output neurons, respectively. We consider the relationship between activities in adjacent layers for ANNs given by

(5) $$x^{l}=w^{l-1}f\left(x^{l-1}\right),$$

and the energy function for EBNs described by

(6) $$E^{l}=\frac{1}{2}{\left(x^{l}-w^{l-1}f\left(x^{l-1}\right)\right)}^{2}.$$

This defines the ANNs to be the standard multilayer perceptrons (MLPs) and the energy-based networks to be the predictive coding network. In Eq. (6) and below, the square operator (***v***)^2^ denotes the inner product of vector ***v*** with itself. The comparison between backpropagation and prospective configuration in the main text is thus between the above MLPs and predictive coding networks; this choice is justified as (1) they are the most standard models^61^ and (2) it is established that the two are closely related^12,14^ (that is, they make the same prediction with the same weights and input pattern), thus enabling a fair comparison. Nevertheless, we show that the theory (Supplementary Fig. 5) and empirical comparison (Supplementary Figs. 6 and 7) between backpropagation and prospective configuration generalize to other choices of network structures and energy functions, that is, other energy-based networks and ANNs, such as GeneRec^62^ and Almeida–Pineda^63–65^.

Putting Eqs. (5) and (6) into the general framework, we can obtain the equations that describe MLPs and predictive coding networks, respectively. Assume that the input and target patterns are ***s***^in^ and ***s***^target^, respectively. Prediction with MLPs is

(7) $$x^{1}=s^{\text{in}}\;\text{and}\;x^{l}=w^{l-1}f\left(x^{l-1}\right)\;\text{for}\;l>1,$$

where ***x***^*L* + 1^ is the prediction. Training MLPs with backpropagation is described by

(8) $$\text{Δ}w^{l}=-\alpha \frac{\partial L}{\partial w^{l}}=-\alpha \frac{\partial L}{\partial x^{L+1}}\cdot \frac{\partial x^{L+1}}{\partial x^{L}}\ldots \frac{\partial x^{l+1}}{\partial w^{l}}\text{where}\;L=\frac{1}{2}{\left(s^{\text{target}}-x^{L+1}\right)}^{2},$$

which backpropagates the error $\frac{\partial L}{\partial x^{l}}$ layer by layer from output neurons.

The neural dynamics of predictive coding networks can be obtained using Eq. (2):

(9) $$\text{Δ}x^{l}=-\gamma \frac{\partial E}{\partial x^{l}}=-\gamma \frac{\partial \left(E^{l}+E^{l+1}\right)}{\partial x^{l}}.$$

Similarly, the weight dynamics of predictive coding networks can be found,

(10) $$\text{Δ}w^{l}=-\alpha \frac{\partial E}{\partial w^{l}}=-\alpha \frac{\partial E^{l+1}}{\partial w^{l}}.$$

To reveal the neural implementation of predictive coding networks, we define the prediction errors to be

(11) $$\varepsilon^{l}=x^{l}-w^{l-1}f\left(x^{l-1}\right).$$

The neural and weight dynamics of predictive coding networks can be expressed (by evaluating derivatives in Eqs. (9) and (10)) as

(12) $$\text{Δ}x^{l}=-\gamma \varepsilon^{l}+f\prime \left(x^{l}\right)\circ {\left(w^{l}\right)}^{T}\varepsilon^{l+1}\;\text{and}$$

(13) $$\text{Δ}w^{l}=\alpha \varepsilon^{l+1}{\left(f\left(x^{l}\right)\right)}^{T},$$

where the symbol ∘ denotes element-wise multiplication. Assuming that ***ε**^l^* and ***x**^l^* are encoded in the activity of error and value neurons, respectively, Eqs. (11) and (12) can be realized with the neural implementation in Fig. 2c. In particular, error ***ε*** and value ***x*** neurons are represented by red and blue nodes, respectively; excitatory + and inhibitory − connections are represented by connections with solid and hollow nodes, respectively. Thus, Eqs. (11) and (12) are implemented with red and blue connections, respectively. It should also be noted that the weight dynamics are also realized locally. The weight change described by Eq. (13) corresponds to simple Hebbian plasticity^66^ in the neural implementation of Fig. 2c; that is, the change in a weight is proportional to the product of activity of presynaptic and postsynaptic neurons. Thus, a predictive coding network, as an energy-based network, can be implemented with local circuits only due to the local nature of energy terms (as argued earlier in this section). Note that when the network is expressive enough such that learning can reduce the energy *E* to 0, the loss L must also become 0 as L is one of the terms in energy *E*, that is $L=E^{L+1}$, and, in this case, the predictive coding network is guaranteed to minimize the loss, just like backpropagation^67^.

The full algorithm of the predictive coding network is summarized in Algorithm 1. In all simulations in this paper (unless stated otherwise), the integration step of the neural dynamics (that is, relaxation) is set to *γ* = 0.1, and the relaxation is performed for 128 steps (𝒯 in Algorithm 1). During relaxation, if the overall energy is not decreased from the last step, the integration step is reduced by 50%; if the integration step is reduced two times (that is, reaching 0.025), relaxation is terminated early. By monitoring the number of relaxation steps performed, we notice that in most of the tasks we performed, relaxation is terminated early at around 60 iterations.

Algorithm 1 **Learn with a predictive coding network^12,18^**

    **Input:** input pattern ***s***^in^; target pattern ***s***^target^; synaptic weights {***w***^1^, ***w***^2^, …, ***w**^L^*}

    **Output:** updated synaptic weights {***w***^1^, ***w***^2^, …, ***w**^L^*}

1 ***x***^1^ = ***s***^in^;                           // Clamp input neurons to input pattern

2 ***x***^*L*+1^ = ***s***^target^;                // Clamp output neurons to target pattern

3 **for** *l* = 2; *l* < *L* + 1; *l* = *l* + 1 **do**                              // Initialize ***x***

4      ***x**^l^* = **0**;

5 **end**

6 **for** *t* = 0; *t* < *τ*; *t* = *t* + l **do**                                           // Relaxation

7      **for** *l* = 1; *l* < *L* + 1; *l* = l + 1 **do**

8         ***ε***^*l*+1^ = ***x***^*l*+1^ – ***w**^l^f* (***x**^l^*) ;                     // according to Eq. (11)

9      **end**

10    **for** *l* = 2; *l* < *L* + 1; *l* = *l* + 1 **do**

11         $\text{Δ}x^{l}=\gamma \left(-\varepsilon^{l}+f\prime \left(x^{l}\right)\circ \left({\left(w^{l}\right)}^{T}\varepsilon^{l+1}\right)\right)\;$;                                                     // according to Eq. (12)

12         ***x**^l^* = ***x**^l^* + Δ***x**^l^*;

13    **end**

14 **end**

15 **for** *l* = 1; *l* < *L* + 1; *l* = *l* + 1 **do**                    // Update weights

16      $\text{Δ}w^{l}=\alpha \varepsilon^{l+1}{\left(f\left(x^{l}\right)\right)}^{T}$;

17      ***w**^l^* = ***w**^l^* + Δ***w*^*l*^**;

18 **end**

In the Supplementary Information, we also investigate other choices of network structures and energy functions, resulting in other ANNs and energy-based networks. Overall, the energy-based networks investigated include predictive coding networks^12,18^, target predictive coding networks and GeneRec^62^, and the ANNs investigated include backpropagation and Almeida–Pineda^63–65^. Details of all the models can be found in corresponding previous work and are also given in the Supplementary Notes, Section 2.1.

### Interference and measuring interference (that is, target alignment)

In Fig. 3a, because it simulates the example in Fig. 1, the network has one input neuron, one hidden neuron and two output neurons; weights were all initialized to 1, the input pattern was [1], and the target pattern was [0, 1]. Learning rates of both learning rules were 0.2, and the weights were updated for 24 iterations. Fig. 3d repeated the same experiment as in Fig. 3a but with the learning rate searched from (0.005, 0.01, 0.05, 0.1), which is wide enough to cover essentially all learning rates used to train deep neural networks in practice.

In Fig. 3e, there were 64 neurons in each layer (including input and output layers) for each network; weights were initialized via standard Xavier uniform initialization^68^. No activation function was used, that is, linear networks were investigated. Depths of networks (*L*) took values from {1, 2, …, 24, 25}, as reported on the *x* axis. Input and target patterns were a pair of randomly generated patterns with a mean of 0 and standard deviation (s.d.) of 1. Learning rates of both learning rules were 0.001. Weights were updated for one iteration, and target alignment was measured. The whole experiment was repeated 27 times with each individual experiment reported as a point.

Simulations in Fig. 3f–h followed the experimental setup in Fig. 4a–h; these are described at the end of Biologically relevant tasks.

### Biologically relevant tasks

In supervised learning simulations, fully connected networks in Fig. 4a–h were trained and tested on FashionMNIST^60^, and convolutional neural networks^35^ (Fig. 4i,j) were trained and tested on CIFAR-10 (ref. 36). With FashionMNIST, models were trained to perform classification of gray-scaled fashion item images into ten categories, such as trousers, pullovers and dresses. FashionMNIST was chosen because it is of moderate and appropriate difficulty for multilayer non-linear deep neural networks so that the comparisons with energy-based networks are informative. Classification of the data in CIFAR-10 is more difficult, as it contains colored natural images belonging to categories such as cars, birds and cats and is thus only evaluated with convolutional neural networks. Both datasets consist of 60,000 training examples (that is, training set) and 10,000 test examples (that is, test set).

The experiments in Fig. 4a–h followed the configurations described below, except for the parameters investigated in specific panels (such as batch size, size of the dataset and size of the architecture), which were adjusted as stated in the descriptions of the specific experiments. The neural network was composed of four layers and 32 hidden neurons in each hidden layer. Note that the state-of-the-art MLP models of FashionMNIST are all quite large^69^. However, they are highly overparameterized and thus are not suitable to base our comparison on because the accuracy reaches more than 95% regardless of the learning rule due to the overparameterization. Thus, there was no space for demonstrating any meaningful comparison in these state-of-the-art overparameterized models. Overall, the size of the model on FashionMNIST demonstrated in this paper was a reasonable choice, with baseline models reaching reasonable performance (~0.12 test error for the standard machine learning setup) while maintaining enough room for demonstrating performance differences for different learning rules. The size of the input layer was 28 × 28 for FashionMNIST^60^ gray scaled, and the size of the output layer was ten as the number of classes for both datasets. The weights were initialized from a normal distribution with a mean of 0 and s.d. of $\sqrt{\frac{2}{n^{l}+n^{l+1}}}$, where *n^l^* and *n*^*l* + 1^ are the numbers of neurons in the layer before and after the weight, respectively. This initialization is known as Xavier normal initialization^68^. The activation function *f* () is sigmoid. We defined one iteration as updating the weights for one step based on a minibatch. Each iteration contained (1) a numerical integration procedure of relaxation of energy-based networks, which captures its continuous process; and (2) one update of weights at the end of the above procedure. The number of examples in a minibatch, called the batch size, was by default 32. One epoch comprised presenting the entire training set split over multiple minibatches. At the end of each epoch, the model was tested on the test set, and the classification error was recorded as the ‘test error’ of the epoch. The neural network was trained for 64 epochs, thus yielding 64 test errors. The mean of the test error over epochs, that is, during training progress, is an indicator of how fast the model learns, and the minimum of the test errors over epochs is an indicator of how well the model can learn, ignoring the possibility of overfitting due to training for too long. Learning rates were optimized independently for each configuration and each model. Each experiment was repeated ten times (unless stated otherwise), and the error bars represent the 68% confidence interval computed using bootstrap.

We now describe settings specific to individual experiments. In Fig. 4b, different batch sizes were tested (as shown on the *x* axis). In Fig. 4c, the batch size was set to 1. In continual learning of Fig. 4d, training alternated between two tasks. Task 1 involved classifying five randomly selected classes in a dataset, and task 2 involved classifying the remaining five classes. The whole network was shared by the two tasks; thus, different from the network used in other panels, the network only had five output neurons. This better corresponds to continual learning with multiple tasks in nature, because, for example, if humans learn to perform two different tasks, they typically use one brain and one pair of hands (that is, the whole network is shared), as they do not have two different pairs of hands (that is, humans share the output layers across tasks). Task 1 was trained for four iterations, task 2 was trained for four iterations, and the training continued until a total of 84 iterations was reached. After each iteration, error on the test set of each task was measured as ‘test error’. In Fig. 4e, the mean of test error of both tasks during training of Fig. 4d at different learning rates is reported. In Fig. 4d–g investigating concept drifting^31,70,71^, changes to class labels were made every 64 epochs, and the models were trained for 3,000 epochs in total. Thus, every 64 epochs, five of ten output neurons were selected, and the mapping from these five output neurons to the semantic meaning was pseudorandomly shuffled. In Fig. 4h, different numbers of data points per class (shown on the *x* axis) were included in the training set (subsets were randomly selected according to different seeds).

In Fig. 4i, we trained a convolutional network with prospective configuration and backpropagation, with the structure detailed in Fig. 4j. For each learning rule, we independently searched seven learning rates ranging from {0.0005, 0.00025, 0.0001, 0.000075, 0.00005, 0.000025, 0.00001}. Both learning rules were trained for 80 epochs, with a batch size of 200. Because training deep convolutional networks is more difficult and slower than training shallow fully connected networks, a few improvements were applied to both learning rules. Specifically, a weight decay of 0.01 and an Adam optimizer^72^ were applied for both learning rules. To reduce running time, the weights were updated more frequently in predictive coding networks; that is, the weights were updated at all steps of inference instead of at the last step of inference. Inference was run for a fixed number of 16 iterations; thus, weights were updated 16 times for each batch of data. Thus, for fair comparison, backpropagation also updated weights 16 times on each batch of data. Training in each configuration (each learning rule and each learning rate) was repeated three times with different seeds.

To extend a predictive coding network to a convolutional neural network (or to any network with a layered structure^58,73^), we can define the forward function of a layer (that is, how the input of layer *l* + 1 is computed from the neural activity of layer *l*) with weights ***w**^l^* to be $F_{w^{l}}\left(x^{l}\right)$. For example, for the MLPs described above, $F_{w^{l}}\left(x^{l}\right)=w^{l}f\left(x^{l}\right)$. For a convolutional network, $F_{w^{l}}\left(x^{l}\right)$ is a more complex function of ***w**^l^* and ***x**^l^*, and also ***w**^l^* and ***x**^l^* are not simple matrix and vector anymore (to be defined later). Defining an ANN with F() would be (that is, Eq. (5) becomes) $x^{l}=F_{w^{l-1}}\left(x^{l-1}\right)$. Defining an energy function of a predictive coding network with F() would be (that is, Eq. (6) becomes) $E^{l}=\frac{1}{2}{\left[x^{l}-F_{w^{l-1}}\left(x^{l-1}\right)\right]}^{2}$. Thus, neural and weight dynamics would be (that is, Eqs. (12) and (13) become) $\text{Δ}x^{l}=-\gamma \varepsilon^{l}+\frac{\partial F_{w^{l}}\left(x^{l}\right)}{\partial x^{l}}\varepsilon^{l+1}$ and $\text{Δ}w^{l}=\alpha \varepsilon^{l+1}\frac{\partial F_{w^{l}}\left(x^{l}\right)}{\partial w^{l}}$, respectively. As $F_{w^{l}}\left(x^{l}\right)$ is defined, $\frac{\partial F_{w^{l}}\left(x^{l}\right)}{\partial x^{l}}$ and $\frac{\partial F_{w^{l}}\left(x^{l}\right)}{\partial w^{l}}$ are obtained via auto differentiation in PyTorch (https://pytorch.org/tutorials/beginner/basics/autogradqs_tutorial.html). Thus, training a convolutional predictive coding network is as simple as replacing lines 11 and 16 in Algorithm 1 with the above corresponding equations.

In the following, we define $F_{w^{l}}\left(x^{l}\right)$ for convolutional networks. First, $x^{l}\in {\mathbb{R}}^{c_{l}\times h_{l}\times w_{l}}$, where *c_l_*, *h_l_* and *w_l_* are the number of features, height and width of the feature map, respectively. The numbers for each layer are presented in Fig. 4j in the format *c_l_@**h**_l_* × ***w**_l_*. For example, for the first layer (input layer), the shape was 3*@*32 × 32 as it is 32 × 32 colored images, that is, with three feature maps representing red, green and blue. We denote kernel size, stride and padding of this layer as *k_l_*, *s_l_* and *p_l_*, respectively. The numbers for each layer are presented in Fig. 4j. Thus, $w^{l}\in {\mathbb{R}}^{c_{l+1}\times c_{l}\times k_{l}\times k_{l}}$. Finally, ***x***^*l* + 1^ is obtained via

(14) $$x^{l+1}\left[c,x,y\right]=f\left(x^{l}\left[:,xs_{l}-p_{l}:xs_{l}-p_{l}+k_{l},ys_{l}-p_{l}:ys_{l}-p_{l}+k_{l}\right]\right)\cdot w^{l}\left[c,:,:,:\right],$$

where [*a*, *b*,…] means indexing the tensor along each dimension, : means all indexes at that dimension, *a*: *b* means slice of that dimension from index *a* to *b* − 1, and · is dot product. In the above equation, if the slicing of ***x**^l^* on the second and third dimensions, that is, ***x**^l^* [:, *xs_l_* − *p_l_* : *xs_l_* − *p_l_* + *k_l_*, *ys_l_* − *p_l_* : *ys_l_* − *p_l_* + *k_l_*], is outside its defined range ${\mathbb{R}}^{c_{l}\times h_{l}\times w_{l}}$, the entries outside range are considered to be 0, known as padding mode of zeros.

In Fig. 3f, networks of 15 layers were trained and tested on the FashionMNIST^60^ dataset. Learning rates in Fig. 3f were optimized independently by a grid search over (5.0, 1.0, 0.5, 0.1, 0.05, 0.01, 0.005, 0.001, 0.0005, 0.0001, 0.00005, 0.00001, 0.000005) for each learning rule, as shown Fig. 3g; that is, each learning rule in Fig. 3f used the learning rate that gave a minimal point in the corresponding curve in Fig. 3g. The experiment in Fig. 3h investigated other network depths ({1, 2, 4, 6, 8, 10, 12, 14, 15}) in the same setup. Similar to Fig. 3f, the learning rate for each learning rule and each ‘number of layers’ was the optimal value (in terms of mean of test error as the *y* axis of the figure) independently searched from (5.0, 1.0, 0.5, 0.1, 0.05, 0.01, 0.005, 0.001, 0.0005, 0.0001, 0.00005, 0.00001, 0.000005). Hidden layers were always of size 64 in the above experiments. In the above experiment, only a part of the training set was used (60 data points per class) so that the test error was evaluated more frequently to reflect the difference on efficiency of the investigated learning rules. The activation function *f* () used is LeakyReLU instead of the standard sigmoid because sigmoid results in difficulty in training deep neural networks. Other unmentioned details followed the defaults, as described above.

In the reinforcement learning experiments (Fig. 4k), we evaluated performance on three classic reinforcement learning problems: Acrobot^74,75^, MountainCar^76^ and CartPole^77^. We interacted with these environments via a unified interface by OpenAI Gym^78^. The observations *s_t_* of these environments are vectors describing the status of the system, such as velocities and positions of different moving parts (for details, refer to the original articles or documentation from OpenAI Gym). Each entry of the observation *s_t_* is normalized to mean 0 and s.d. 1 via Welford’s online algorithm^79,80^. The action space of these environments is discrete. Thus, we can have a network taking in observation *s_t_* and predicting the value (*Q*) of each action *a_t_* with different output neurons. Such a network is known as an action-value network, in short, a *Q* network. In our experiment, the *Q* network contained two hidden layers, each of which contained 64 neurons, initialized the same way as the network used for supervised learning, described before. One can acquire the value of an action *a_t_* at a given observation *s_t_* by feeding *s_t_* into the *Q* network and reading out the prediction on the output neuron corresponding to the action *a_t_*; such a value is denoted *Q* (*s_t_*, *a_t_*). The training of *Q* is a simple regression problem to target ${\hat{R}}_{t}$, obtained via *Q* learning with experience replay (summarized in Algorithm 2). Considering *s_t_* to be ***s***^in^ and ${\hat{R}}_{t}$ to be ***s***^target^, the *Q* network can be trained with prospective configuration or backpropagation. Note that ${\hat{R}}_{t}$ is the target of the selected action *a_t_* (that is, the target of one of the output neurons corresponds to the selected action *a_t_*); thus, ${\hat{R}}_{t}$ is, in practice, considered to be ***s***^target^ [*a_t_*]. For prospective configuration, it means that the rest of the output neurons except the one corresponding to *a_t_* are freed; for backpropagation, it means that the error on these neurons is masked out.

A predictive coding network with slightly different settings from the defaults was used for prospective configuration. The integration step was fixed to be half of the default (*γ* = 0.05), and relaxation was performed for a fixed and smaller number of steps (𝒯 = 32). This change was introduced because *Q* learning is more unstable (smaller integration step) and more expensive (smaller number of relaxation steps) than supervised learning tasks. To produce a smoother curve of ‘sum of rewards per episode’ in Fig. 4k from *SumRewardPerEpisode* in Algorithm 2, the *SumRewardPerEpisode* curve was averaged along *TrainingEpisode* with a sliding window with a length of 200. Each experiment was repeated with three random seeds, and the shadows represent 68% confidence interval across them. Learning rates were searched independently for each environment and each model from the range {0.05, 0.01, 0.005, 0.001, 0.0005, 0.0001}. The results reported in Fig. 4k are for the learning rates yielding the highest mean of ‘sum of rewards per episode’ over training episodes.

Algorithm 2 ***Q* learning with experience replay**

    **Input:** Action-value network *Q*.

    **Result:** Trained action-value network *Q*.

1 Initialize experience replay R of capacity 50000;

2 **for**

      *Training Episode* = 0; *Training Episode* < 10000; *Training Episode* = *Training Episode* + 1

      **do**

3         *ρ* = max (0.01, 0.08 – 0.01 ∗ (*Training Episode*/200));       // Anneal probability of exploring

4         Get initial observation *s_t_* and set episode termination signal *d_t_* = *False*;

5         Initialize *Sum Reward Per Episode* = 0;

6         **while** ! *d_t_* **do**                                                                                             // Collect experience

7             With probability *ρ* sample a random action *a_t_*, otherwise select *a_t_* = arg max_*a*_ *Q* (*s_t_*, *a*);

8             Execute *a_t_*, observe reward *r_t_*, new observation *r*_*t*+1_ and *d_t_*;

9             Accumulate *Sum Reward Per Episode*+ = *r_t_*;

10           Store transition (*s_t_*, *a_t_*, *r_t_*, *s*_*t*+1_, *d_t_*) in R;

11           Set *s_t_* = *s*_*t*+1_;

12       **end**

13       **if** *length* (R) > 2000 **then**                                                                                  // Replay and train

14             **for** *epoch* = 0; *epoch* < 10; *epoch* = *epoch* + 1 **do**

15                   Sample random minibatch (size=60) of (*s_t_*, *a_t_*, *r_t_*, *s*_*t*+1_, *d_t_*) from R;

16                    ${\hat{R}}_{t}=\{\begin{array}{ll} r_{t} & \text{if}\;d_{t}==True\\ r_{t}+0.98{\;\max}_{a}Q\left(s_{t+1},a\right), & \text{otherwise} \end{array};$

17                   Set ***s***^in^ = *s_t_*;

18                   Set $s^{\text{target}}\left[a_{t}\right]={\hat{R}}_{t}$;

19                   Train *Q* with ***s***^in^ and ***s***^target^ [*a_t_*] with prospective configuration or backpropagation;

20             **end**

21         **end**

22         *Report Sum Reward Per Episode*;

23 **end**

### Simulation of motor learning

As shown in Fig. 5, we trained a network that included two input neurons, two hidden neurons and two output neurons. The two input neurons were one-to-one connected to the two hidden neurons, and the two hidden neurons were fully connected to the two output neurons. The two input neurons were considered to encode presenting the blue and red background, respectively. The two output neurons were considered to encode the prediction of the perturbations toward positive and negative directions, respectively. Presenting and not presenting a background color were encoded 1 and 0, respectively; presenting and not presenting perturbations of a particular direction were encoded 1 and 0, respectively. The weights were initialized from a normal distribution with mean 0 and an s.d. fitted to the behavioral data (see below), simulating that the participants had not built any associations before the experiments. Learning rates were independent for the two layers, as we expected the connections from perception to belief and from belief to predictions to have different degrees of plasticity. The two learning rates were also fitted to the data (see below).

The number of participants and training and testing trials follow exactly as described for the human experiment^38^. In particular, for each of the 24 simulated participants, the weights were initialized with a different seed of the random number generator. They each experienced two stages: training and testing. Note that the pretraining stage performed in the human experiment was not simulated here as its goal was to make human participants familiar with the setup and devices.

In the training stage, the model experienced 24 blocks of trials. In each block, the model was presented with the following sequence of trials, matching the original experiment^38^:

- The model was trained with two trials without perturbation, B_0_ and R_0_, with the order counterbalanced across consecutive blocks. Note that, in the human experiment, there were two trial types without perturbations (channel and washout trials), but they were simulated in the same way here as B_0_ or R_0_ trials because they both did not include any perturbations.
- The model was trained with 32 trials with perturbations, where there were equal numbers of B+ and R– within each of the 8 trials in a pseudorandom order.
- The model experienced two trials, B_0_ and R_0_, with the order counterbalanced across consecutive blocks.
- The model experienced *n* ← {14, 16, 18} washout trials (equal numbers of B_0_ and R_0_ trials in a pseudorandom order), where *n* ← {*a*, *b*, *c*} denotes sampling without replacement from a set of values *a*, *b* and *c* and replenishing the set whenever it becomes empty.
- The model experienced one triplet, where the exposure trial was either B+ or R–, counterbalanced across consecutive blocks. Here, a triplet consisted of three sequential trials: B_0_, the specified exposure trial and B_0_ again.
- The model experienced additional *n* ← {6, 8, 10} washout trials (equal numbers of B_0_ and R_0_ trials in a pseudorandom order).
- The model experienced one triplet again, where the exposure trial was either B+ or R–, whichever was not used on the previous triplet.

In the testing stage, the model then experienced eight repetitions of four blocks of trials. In each block, one of the combinations of B+, R+, B– and R– was tested. The order of the four blocks was shuffled in each of the eight repetitions. In each block, the model first experienced *n* ← {2, 4, 6} washout trials (equal numbers of B_0_ and R_0_ trials in a pseudorandom order). The model then experienced a triplet of trials, where the exposure trial was the combination (B+, R+, B– or R–) tested in a given block to assess single-trial learning of this combination. The change in adaption in the model was computed as the absolute value of the difference in the predictions of perturbations on the two B_0_ trials in the above triplet, where the prediction of perturbation was computed as the difference between the activities of the two output neurons. The predictions were averaged over participants and the above repetitions.

The parameters of each learning rule were chosen such that the model best reproduced the change in adaptation shown in Fig 5f. In particular, we minimized the sum over set *C* of the four exposure trial types of the squared difference between average change in adaptation in experiment (*d_c_*) and model (*x_c_*):

(15) $$\sum_{c\in C}{\left(ax_{c}-d_{c}\right)}^{2}.$$

The model predictions were additionally scaled by a coefficient *a* fitted to the data because the behavioral data and model outputs had different scales. An exhaustive search was performed over model parameters. The s.d. of initial weights could take values from {0.01, 0.05, 0.1}, and two learning rates for two layers could take values from {0.00005, 0.0001, 0.0005, 0.01, 0.05}. For each learning rule and each combination of the above model parameters, the coefficient *a* was then resolved analytically (restricted to be positive) to minimize the sum of the squared errors of Eq. (15).

### Simulation of human reinforcement learning

As shown in Fig. 6b, we trained a network that included one input neuron, one hidden neuron and two output neurons. The input neuron was considered to encode being in the task, so it was set to 1 throughout the simulation. The two output neurons encoded the prediction of the value of the two choices. Reward and punishment were encoded as 1 and −1, respectively, because the participants were either winning or losing money. The model selected actions stochastically based on the predicted value of the two choices (encoded in the activity of two output neurons) according to the softmax rule (with a temperature of 1). The weights were initialized from a normal distribution of mean 0 and an s.d. fitted to experimental data (see below), simulating that the human participants had not built any associations before the experiments. The number of simulated participants (number of repetitions with different seeds) was set to 16, as in the human experiment^38^. The number of trials was not mentioned in the original paper, so we simulated for 128 trials for both learning rules.

To compare the ability of the two learning rules to account for the pattern of signal from the mPFC, for each of the rules, we optimized the parameters describing how the model is set up and learns (the s.d. of initial weights and the learning rate). Namely, we searched for the values of these parameters for which the model produces the most similar pattern of its output activity to that in the experiment. In particular, we minimized the sum over set *C* of four trial types in Fig. 6c of the squared difference between model predictions *x_c_* and data *d_c_* on mean mPFC signal:

(16) $$\sum_{c\in C}{\left(ax_{c}+b-d_{c}\right)}^{2}.$$

The model predictions were additionally scaled by a coefficient *a* and offset by a bias *b* because the fMRI signal had different units and baseline than the model. To compute the model prediction for a given trial type, the activity of the output neuron corresponding to the chosen option was averaged across all trials of this type in the entire simulation. The scaled average activity from the model is plotted in Fig. 6c, where the error bars show the 68% confidence interval of the scaled activity. To fit the model to experimental data, the values of model parameters and the coefficient were found as described in the previous section. In particular, we used exhaustive grid search on the parameters. The models were simulated for all possible combinations of s.d. of initial weights and the learning rate from the following set: {0.01, 0.05, 0.1}. For each learning rule and each combination of the above model parameters, the coefficient *a* (restricted to be positive) and the bias *b* were then resolved analytically to minimize the sum of the squared error of Eq. (16).

### Statistics and reproducibility

The work in this paper involved computer simulations, but due to random initialization of weight parameters, the simulations were repeated multiple times. No statistical method was used to predetermine the number of repetitions, but for simulations corresponding to behavioral or neurophysiological experiments, the number of repetitions was matched to the number of participants in the given experiment. No data were excluded from the analyses. Because the order of execution has no effect on the results of the numeric experiments, they were not randomized. The investigators were not blinded to outcome assessment.

To visualize the variability of simulation results, we either presented individual data points or error bars showing confidence intervals or box plots. Confidence intervals were computed using bootstrap throughout the paper, and detailed descriptions of the implementation can be found at https://seaborn.pydata.org/tutorial/error_bars.html#confidence-interval-error-bars. The details of the methods used to produce the box plots are available at https://seaborn.pydata.org/generated/seaborn.boxplot.html.

### Reporting summary

Further information on research design is available in the Nature Portfolio Reporting Summary linked to this article.

## Supplementary Material

Supplementary Information

Supplementary Materials

## Data Availability

Learning tasks analyzed in Fig. 4a–j were built using the publicly available FashionMNIST^60^ and CIFAR-10 (ref. 36) datasets. These datasets are incorporated in most machine learning libraries, and their original releases are available at https://github.com/zalandoresearch/fashion-mnist and https://www.cs.toronto.edu/~kriz/cifar.html, respectively. Reinforcement learning tasks analyzed in Fig. 4i were built using the publicly available simulators by OpenAI Gym^78^. Source data are provided with this paper.

## Code availability

Complete code and full documentation reproducing all simulation results written in Python are publicly available at https://github.com/YuhangSong/Prospective-Configuration released under GNU General Public License v3.0 without any additional restrictions (for license details, see https://opensource.org/licenses/GPL-3.0 by the open source initiative).
