# Supplementary material for: Inferring neural activity before plasticity as a foundation for learning beyond backpropagation
Source: Nat Neurosci. 2024 Jan 3;27(2):348–58. doi: 10.1038/s41593-023-01514-1 (PMC7615830; doi:10.1038/s41593-023-01514-1)
Supplement: Supplementary file 1 [file 41593_2023_1514_MOESM1_ESM.pdf]

nature neuroscience

Article https://doi.org/10.1038/s41593-023-01514-1

Inferring neural activity before plasticity as a foundation for learning beyond backpropagation

In the format provided by the authors and unedited

Inferring neural activity before plasticity as a foundation for learning beyond backpropagation

1 Supplementary Information

This document contains Supplementary Figures referred in the paper, followed by Supplementary Notes with additional description and analysis of the simulated models.

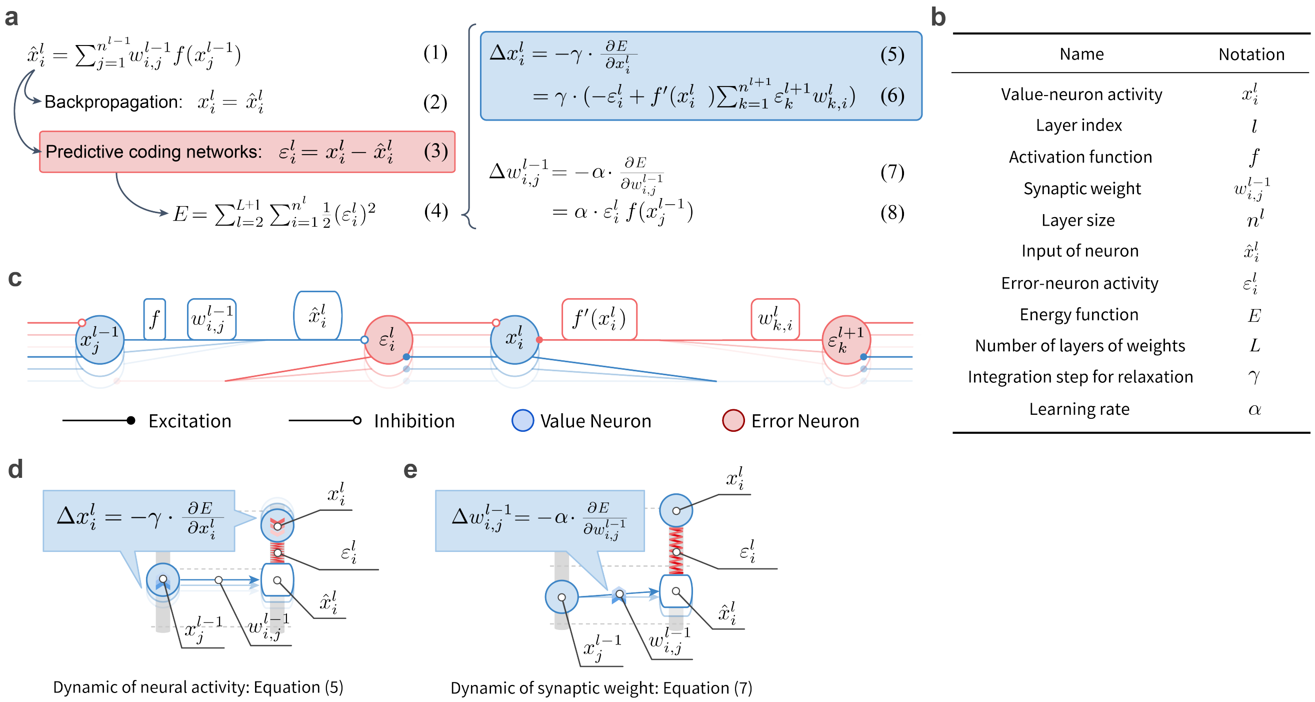

Supplementary Fig. 1

Predictive coding networks, neural implementation and corresponding energy machine. The figure shows a list of the equations describing the equilibrium-seeking dynamics and plasticity of predictive coding networks (panels a-b), how these equations map to a neural implementation, and how they map to the machine analog introduced in Fig. 2.

▶ **a** | List of equations describing predictive coding networks. Eq. (1) in this figure describes the input to a given layer $\hat{x}_i^l$ from the neurons in the previous layer x_j^{l-1}. In artificial neural networks trained with backpropagation, neural activities of a given layer x_i^l are set as the input to this layer $\hat{x}_i^l$ (Eq. (2)). In contrast, in predictive coding networks, neural activities of this layer x_i^l are **not** set as the input to this layer $\hat{x}_i^l$, instead an error ε_i^l is defined between them (Eq. (3)). Additionally, predictive coding networks define the energy E of the network to be the sum of all the squared errors $\frac{1}{2}\left(\varepsilon_i^l\right)^2$ (Eq. (4)). The dynamic of neural activity Δx_i^l in predictive coding networks is set to change the neural activity in proportion to the negative gradient of the energy with respect to the neural activity, so as to reduce the energy (Eq. (5)), which can be further derived as Eq. (6). The dynamic of synaptic weights $\Delta w_{i,j}^{l-1}$ of predictive coding networks is set to be in proportion to the negative gradient of the energy with respect to the weight, so as to reduce the energy (Eq. (7)), which can be further derived as Eq. (8).

▶ **b** | A list of symbols shared by all panels in the figure for easy reference.

▶ **c** | Mapping of equations describing predictive coding networks in panel a to a neural implementation. The neural implementation includes value neurons (blue) performing computations in Eq. (6), and separate error neurons encoding prediction errors (red) performing computations in Eq. (3), where positive sign is encoded by excitatory connections while negative sign is encoded by the inhibitory connections. It should be noticed that the weight dynamics $\Delta w_{i,j}^{l-1}$ is also realized locally: weight change described by Eq. (8). corresponds to simple Hebbian plasticity[1] in the architecture shown in panel a, i.e., the change in a weight is proportional to the product of activity of pre-synaptic and post-synaptic neurons. Different suggestions have been made on how this architecture could be realized in cortical circuits. An influential study[2] has suggested that error and value neurons correspond to separate neurons, so in such architecture the plasticity rule is precisely Hebbian, as explained above. Some other models[3] implementing predictive coding networks[4] include an error compartment (in apical dendrite) and a value compartment (in soma) within a single neuron. In such architecture the plasticity is still local as it depends on the product of activity in one neurons and potential of the apical dendrite in the other neuron.

▶ **d-e** | Mapping of equations describing predictive coding networks in panel a to the machine analog introduced in Fig. 2. The exact same set of equations describing predictive coding networks also describe a physical machine connected with rods, nodes and springs. ▶ **d** | The dynamic of neural activity Δx_i^l of predictive coding networks (Eq. (5)) describes relaxing the physical machine by moving the nodes. ▶ **e** | The dynamic of synaptic weight $\Delta w_{i,j}^{l-1}$ of predictive coding networks (Eq. (7)) describes relaxing the physical machine by tuning the rods.

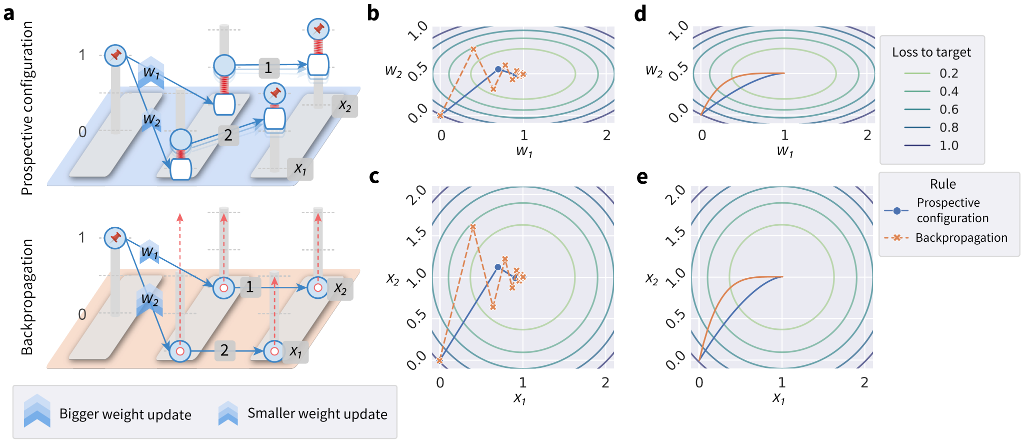

Supplementary Fig. 2

Differences in learning between prospective configuration and backpropagation. This figure shows an example of a simple network revealing striking differences in how errors are propagated and weights modified by the two algorithms. For this network it is possible to explicitly visualize how learning changes weights and outputs, and explicitly show that although backpropagation follows the gradient of loss in the space of weights, it does not in the space of outputs.

▶ **a** | Setup of the example. In this example, we consider a network consisting of 1 input neuron, 2 hidden neurons and 2 output neurons, with the structure shown with the energy machine. The input is always 1 and the target of both output neurons are both 1. The weights in the first layer are initialized to 0, while in the second layer to 1 (top) and 2 (bottom). We visualize with the energy machine how prospective configuration and backpropagation learn differently in this example. Prospective configuration assigns larger error to the top hidden neurons than the bottom, and hence would increase w_1 more than w_2. By contrast, backpropagation does the opposite: since the backpropagated errors are scaled by the weights to output layer, the error for the bottom hidden neuron is higher than for the top. Importantly, in this learning problem, weight w_2 does not need to be modified as much as w_1, because any changes in w_2 will be amplified by the high weight to the output neuron. Prospective configuration indeed modifies w_2 less than w_1, while backpropagation does the opposite. This suggests that backpropagation does not modify the weights optimally to move output toward the target, and we will illustrate it in the following panels.

▶ **b** | Landscape of the weights (w_1 and w_2). We consider a setup in which the network only learns the two weights in the first layer: w_1 and w_2, while the weights in the second layer are fixed all the time during the training. This is so that the weight space is small (only two dimensional, so that we can visualize the landscape of weights); and we choose to learn the two weights in the first layer instead the second (last) layer so that the problem is not trivial. All the combinations of weights on the same contour line gives the same loss to the target (in short, loss), where we can see the combination of $w_1 = 1$ and $w_2 = 0.5$ gives loss of 0. Assuming the weights (w_1, w_2) start from $(0, 0)$, backpropagation (orange) takes steps following the direction orthogonal to the contour lines, i.e. the direction of local gradient descent. It is well-known that backpropagation cannot have more global vision of the minimal point of the landscape: thus, often forms the trajectory of learning as the orange curve, "bouncing" towards the global minimum point. Prospective configuration (blue), on the contrary, although does not follow gradient in the weight space (blue line is not orthogonal to the contour lines), it moves more directly to the global minimum of the landscape. This is exactly due to the mechanism of prospective configuration giving the learning rule a more global view of the system: as mentioned above, prospective configuration infers that since

the bottom weight of second layer is larger (= 2) than the top one (= 1), it only needs small error being assigned to the bottom neuron of the hidden layer so as to correct the error on the bottom output.

▶ **c** | Landscape of the outputs (x_1 and x_2). The panel shows changes in output neurons' activity, x_1 and x_2, resulting from the weight updates in panel b. As in panel b, the contour lines indicate the loss. Comparing panels b and c reveals that changes of backpropagation (orange) are orthogonal to the loss contour lines in weight space, but not in output neuron space; while changes of prospective configuration (blue) are not orthogonal to loss contour lines in weight space, but are closer to being orthogonal in output neuron space. Overall, the comparison reveals fundamental difference between backpropagation and prospective configuration: backpropagation does local gradient decent in weight space (local means it only sees the infinitely small area around it current state); while prospective configuration infers the configuration of neuron activities that reduces the loss in the output space, thus, the trajectory in the weight space is fundamentally different from that for backpropagation. This fundamental difference leads to advantage of prospective configuration over backpropagation: it moves more directly towards the global minimum in the weight space and output space. Learning rate in this panel is the same as the learning rate used in the corresponding learning rule in panel b.

▶ **d-e** | The same experiments as b and c but with a small learning rate $\alpha = 0.05$ for both learning rules. As can be seen in the two panels, the notable difference between the two learning rules persists despite the learning rate being sufficiently small (the trajectory being sufficiently smooth).

Implementation details. In panels b and c, learning rate for backpropagation in this figure is set to $\alpha = 0.4$, while that for prospective configuration is solved so that it produces the same magnitude of weight change ($\sqrt{\Delta w_1^2 + \Delta w_2^2}$) during the first iteration as backpropagation. Weights are updated for 15 iterations in panels b and c, and for 150 iterations in panels d and e. Details of the learning rules are described in the Methods section and also in Supplementary Notes, Section 2.1.

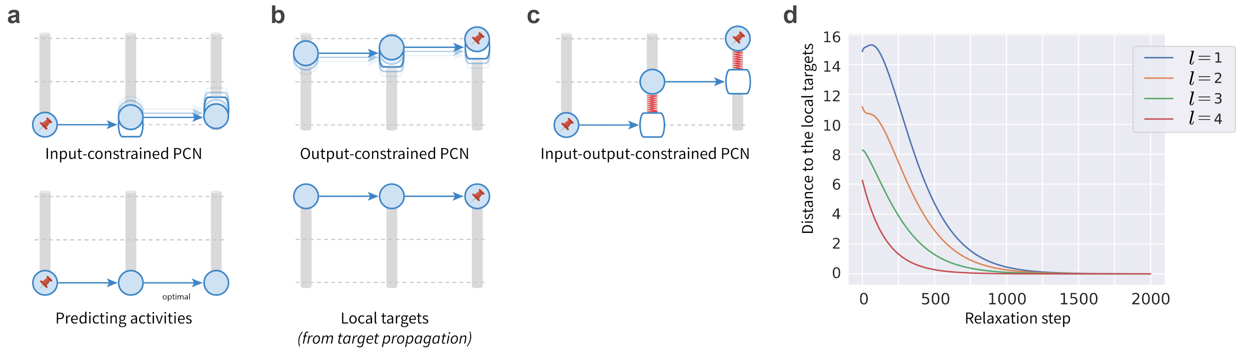

Supplementary Fig. 3

Relationship of prospective configuration to target propagation. Prospective configuration is related to another influential algorithm of credit assignment — target propagation[5]. Since target propagation has target alignment equal to 1[6], this relationship provides an explanation for the high target alignment of prospective configuration. Target propagation is an algorithm, which explicitly computes the neural activity in hidden layers required to produce the desired target pattern. We call these values local targets. We demonstrate that one of energy-based networks, predictive coding networks[7,8] (PCNs) tends to move the activity during relaxation towards these local targets. The relationship of PCNs to target propagation can be visualized with the proposed energy machine in Fig. 2, hence panels a–c illustrate how the neural activity in a PCN depends on whether inputs and outputs are constrained, and these properties are formally proved in Supplementary Notes, Section 2.2.

▶ **a** | With only input neurons constrained (and outputs unconstrained) PCNs can generate prediction about the output, and hence we refer to this pattern of neural activity as the predicting activity.

▶ **b** | With only output neurons constrained (and inputs unconstrained), the neural activity of PCNs relaxes to the local target from target propagation. This happens because with only outputs constrained, other nodes have a freedom to move to values that generate the outputs, and when the energy reduces to 0 (as shown in the bottom display) all neurons must have the activity generating the target output.

▶ **c** | With both input and output neurons constrained, the neural activity of PCNs relaxes to the weighted sum of the local target from target propagation and the predicting activity. Note that the position of the hidden node is in between the positions from panels a and b.

▶ **d** | The distance between the neural activity to the local target at different layers along the relaxation progress in output-constrained PCNs. Here, the neural activity of the output-constrained PCNs converges to the local target, and the layers closer to the output layer (larger l) converge to the local target earlier than the others, which is as expected from the physical intuition of the energy machine.

Implementation details. We train the models to predict a target pattern from an input pattern (both randomly generated from $\mathcal{N}(0,1)$, and the input and target patterns are of 5 and 1 entries, respectively). The structure of the networks is $5 \rightarrow 5 \rightarrow 5 \rightarrow 5 \rightarrow 1$. There is no activation function, i.e., it is a linear network. For the computation of the local target in target propagation, refer to the original paper[5]. The mean square difference is used to measure the distance to the local target.

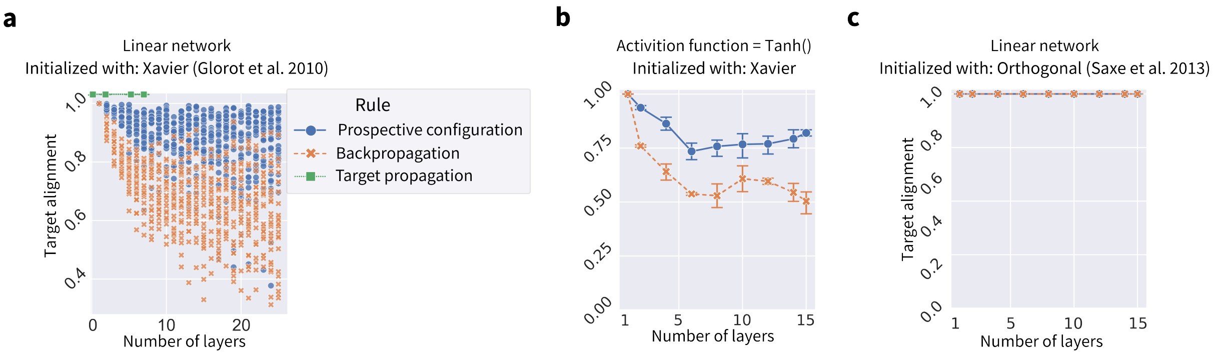

Supplementary Fig. 4

Target alignment in deep neural networks with different learning algorithms, non-linearities and initializations. This figure extends the analyses from Fig. 3e in the main paper of target alignment in randomly generated networks with different depth.

▶ **a** | Target alignment for target propagation in deep linear network initialized with standard Xavier normal initialization[9]. For comparison, the results presented in Fig. 3e of the main paper for predictive coding networks and backpropagation are also shown. The results for target propagation are only shown for networks with up to 5 layers, because the algorithm became numerically unstable for deeper networks. The target alignment of target propagation is equal to 1 as implied by previous analytic work[6] (for details see Supplementary Notes, Section 2.4.2).

▶ **b** | Target alignment for networks with a non-linear (*Tanh*) activation function, initialized with standard Xavier normal initialization[9]. The higher value of target alignment for predictive coding networks than backpropagation shown in panel a generalizes to networks with non-linearity.

▶ **c** | Target alignment of linear networks with orthogonal initialization (where weight in each layer satisfy $\left(\boldsymbol{w}^l\right)^T \boldsymbol{w}^l = \boldsymbol{I}$)[10]. Saxe et al.[10] discovered that with such initialization weights evolve independently of each other during learning, thus, learning times can be independent of depth, even for arbitrarily deep linear networks. As shown in the figure, interestingly, orthogonal initialization gives target alignment of 1 for both learning rules. We also demonstrated this analytically in Supplementary Notes, Section 2.4.3. This perfect target alignment can be intuitively expected, because the independence of weights mentioned above is related to a lack of interference, and it further illustrates that reduction in target alignment is caused by interference between weights.

Each experiment in panels b-c is repeated with $n = 3$ random seeds. Error bars and bands represent the 68% confidence interval.

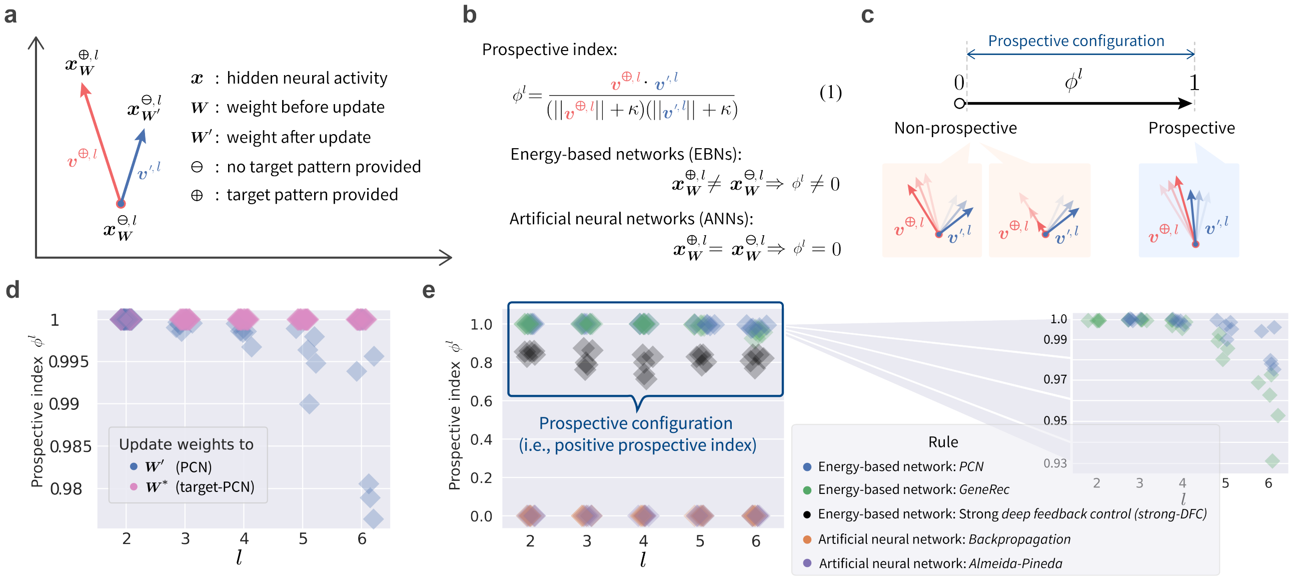

Supplementary Fig. 5

Formal definition of prospective configuration. Formal definition of prospective configuration with prospective index (panels a–c), a metric that one can measure for any learning model. With this metric, we show that prospective configuration is present in different *energy-based networks* (EBNs), but not in *artificial neural networks* (ANNs) (panels d–e).

▶ **a** | To introduce the prospective index, we consider the hidden neural activity $\boldsymbol{x}^l$ in layer l, at three moments of time. First, a learning iteration starts from $\boldsymbol{x}^l$ under the current weights $\boldsymbol{W}$ without target pattern provided $\ominus$: $\boldsymbol{x}_{\boldsymbol{W}}^{\ominus,l}$. Second, a target pattern is provided $\oplus$, and neural activity settles to $\boldsymbol{x}_{\boldsymbol{W}}^{\oplus,l}$. Third, $\boldsymbol{W}$ is updated to $\boldsymbol{W}'$, the target pattern is removed $\ominus$, and the neural activity settles to $\boldsymbol{x}_{\boldsymbol{W}'}^{\ominus,l}$. We define two vectors $\boldsymbol{v}^{\oplus,l}$ and $\boldsymbol{v}^{\prime,l}$, representing the direction of the neural activity changes as a result of the target pattern being given $\ominus \rightarrow \oplus$ and the weights being updated $\boldsymbol{W} \rightarrow \boldsymbol{W}'$, respectively.

▶ **b** | The prospective index ϕ^l is the cosine similarity of $\boldsymbol{v}^{\oplus,l}$ and $\boldsymbol{v}^{\prime,l}$. A small constant $\kappa = 0.00001$ is added in the denominator to ensure that the prospective index is still defined if the length of one of the vectors is 0 (in which case the prospective index in equal to 0). For EBNs, the neural activity settles to a new configuration when the target pattern is provided, i.e., $\boldsymbol{x}_{\boldsymbol{W}}^{\oplus,l} \neq \boldsymbol{x}_{\boldsymbol{W}}^{\ominus,l}$, so ϕ^l is non-zero; for ANNs, the neural activity stays unchanged when the target pattern is provided, i.e., $\boldsymbol{x}_{\boldsymbol{W}}^{\oplus,l} = \boldsymbol{x}_{\boldsymbol{W}}^{\ominus,l}$, so ϕ^l is zero.

▶ **c** | A positive ϕ^l implies that $\boldsymbol{v}^{\oplus,l}$ and $\boldsymbol{v}^{\prime,l}$ are pointing in the same direction, i.e., the neural activity after the target pattern provided $\boldsymbol{x}_{\boldsymbol{W}}^{\oplus,l}$ is similar to the neural activity after the weight update $\boldsymbol{x}_{\boldsymbol{W}'}^{\ominus,l}$, i.e., is prospective. We define the models following the principle of prospective configuration as those with positive ϕ^l (averaged over all layers). Additionally, prospective index close to 1 implies that a weight update rule in a model is able to consolidate the pattern of activity following relaxation, so a similar pattern is reinstated during prediction on the next trial.

▶ **d** | The prospective index ϕ^l of different layers l in PCNs and a variant of PCNs called target-PCNs. Several observations can be made, and they are explained and proved in Supplementary Notes, Section 2.3.

▶ **e** | The prospective index ϕ^l of different EBNs and ANNs. Here, we can see that all EBNs produce positive ϕ^l, i.e., the prospective configuration is commonly observed in EBNs, but not in ANNs. Among the EBNs, *Deep Feedback Control*[11] (DFC) was proposed to work with "infinitely weak nudging", as in equilibrium propagation[12]. More recent work demonstrates that it also works with "strong control"[13,14]

(thus, called strong-DFC), i.e., with the natural form of EBNs. The prospective index was measured for this strong-DFC model and shows it belongs to one of EBNs that process prospective configuration. Details of the simulated strong-DFC model can be found in Supplementary Notes, Section 2.1.

Implementation details. We train various models to predict a target pattern from an input pattern (both randomly generated from $\mathcal{N}(0,1)$). The structure of the networks is $64 \rightarrow 64 \rightarrow 64 \rightarrow 64 \rightarrow 64 \rightarrow 64 \rightarrow 64$. The weights are initialized using Xavier normal initialization[9] (described in the Methods). No activation function was used. Batch size is set to 1. The models were trained for one iteration (i.e., one update of the weights), the prospective index was then measured for this update. Prospective indices of input and output layers are not reported. This is because the input and output layers are held fixed during learning; thus, the prospective index is not defined for them. Experiments were repeated 5 times. The EBNs investigated include PCNs[7,8], target-PCNs, and *GeneRec*[15], while the ANNs investigated include backpropagation and *Almeida-Pineda*[16–18]. Details of all simulated models are given in Supplementary Notes, Section 2.1.

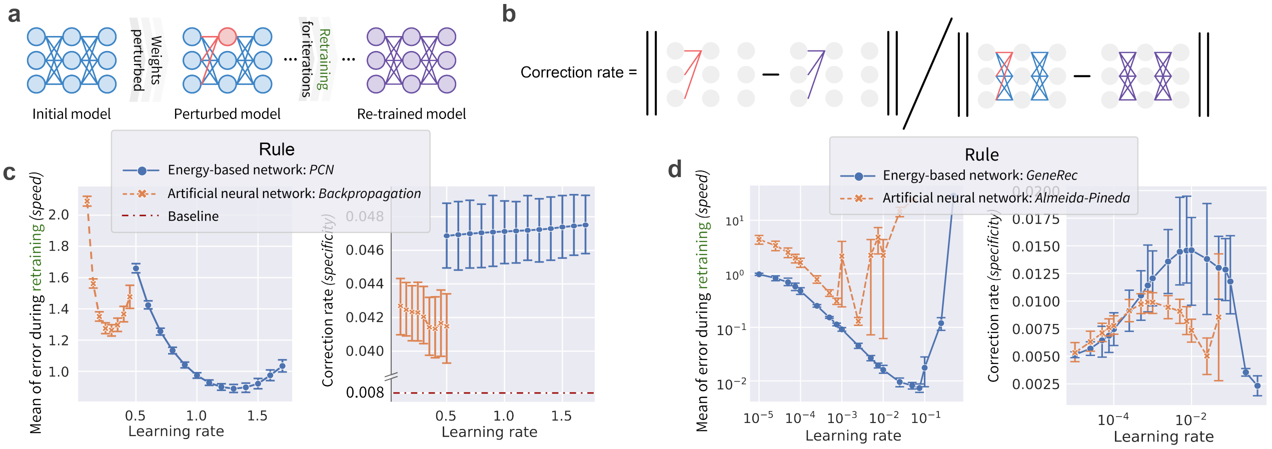

Supplementary Fig. 6

Prospective configuration yields a more accurate weight modification. A numerical experiment (panels a–b) verifies that *energy-based networks* (EBNs) yield a more accurate weight modification than *artificial neural networks* (ANNs) (panels c–d). The following intuition can be provided for why the prospective configuration enables an accurate weight modification. In EBNs, if more error is assigned to a neuron, this neuron will settle to a prospective activity that reduces the error. The prospective activity of this neuron is then propagated through the network, resulting in less error being assigned to other neurons, thus the error being assigned more accurately.

▶ **a** | Experimental procedure: we take a pre-trained model (illustration here does not reflect the real size of the model), randomly select a hidden neuron and perturb the synaptic weights connecting to this neuron (red), then retrain this model on the same pattern for a fixed number of iterations. During retraining, an optimal learning agent is expected to identify that the error in the output neurons is due to the perturbed weights, thus, (1) correct the error faster, and (2) correct the perturbed weights more. We refer to the above two properties as *speed* and *specificity*. Speed can be measured with the mean of error over retraining iterations (the lower, the better).

▶ **b** | Specificity can be measured by correction rate (the higher, the better): the ratio of how much the perturbed weights are corrected compared to how much all the weights (in all layers) are corrected after all retraining iterations.

▶ **c** | A comparison between an EBN, predictive coding network[7,8] (PCN), and an ANN, trained with backpropagation. In the right plot, there is an additional baseline, which is the number of perturbed weights divided by the number of all the weights, indicating the expected correction rate if a learning rule randomly assigns errors.

▶ **d** | The same comparison as in panel c, but for another EBN, namely, *GeneRec*[15]. GeneRec describes learning in recurrent networks, and ANN with this architecture is not trained by standard backpropagation, but by a variant of backpropagation, called *Almeida-Pineda*[16–18].

Implementation details. We first pre-train the models to predict a target pattern from an input pattern (both randomly generated from $\mathcal{N}(0,1)$ and of 32 entries). The structure of the networks is $32 \rightarrow 32 \rightarrow 32 \rightarrow 32$. The pre-training session is sufficiently long (1000 iterations) to reach convergence. Then, one neuron is randomly selected from the $(32+32)$ hidden neurons, and all weights connecting to this neuron are "flipped" (i.e., multiplied by -1). Current weights of the network are recorded as $\boldsymbol{W}_b$. The part of current weights that were just flipped are recorded as $\boldsymbol{W}_b^f$. The network is then re-trained on the same pattern for 64 iterations. After each re-training iteration, the model makes a prediction. The square difference

between the prediction and the target pattern is recorded as the “error during re-training” of this iteration. After the entire re-training session, the “errors during re-training” are averaged over the 64 re-training iterations, producing the left plots of panels c–d. Current weights of the network are recorded as $\boldsymbol{W}_a$. The part of current weights that were flipped before the re-training session are recorded as $\boldsymbol{W}_a^f$. The *correction rate* is computed as $\| \boldsymbol{W}_a^f - \boldsymbol{W}_b^f \| \ / \ \| \boldsymbol{W}_a - \boldsymbol{W}_b \|$, which produces the right plots of panels c–d. Each configuration was repeated $n = 20$ times, and the error bars represent 68% confidence intervals.

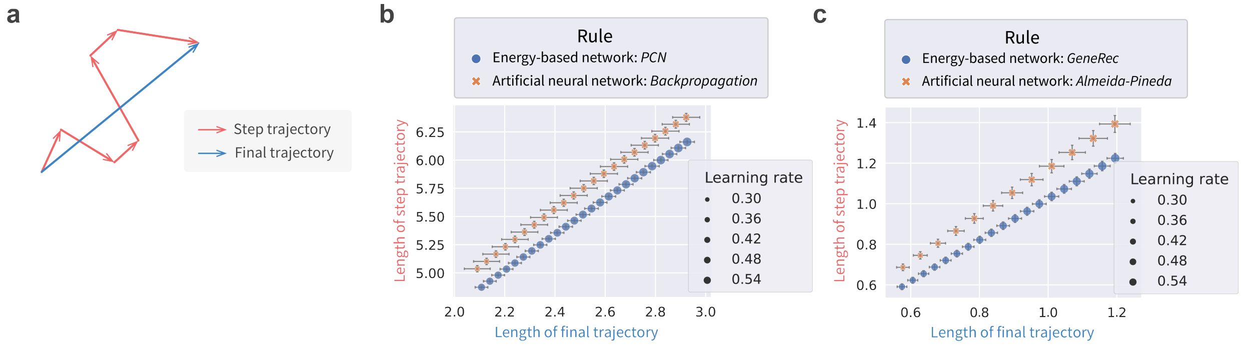

Supplementary Fig. 7

Prospective configuration produces less erratic weight modification. An experiment verifies that *energy-based networks* (EBNs) (i.e., prospective configuration), produce a less erratic weight modification than *artificial neural networks* (ANNs) (i.e., backpropagation).

▶ **a** | Experimental procedure. The weights are updated for a fixed number of steps on a fixed number of data points, which produces the step trajectory in the weight space (each red arrow corresponds to one weights update). Connecting the start and end points of the step trajectory (i.e., the initial and final weights of the model) produces the final trajectory (blue). A learning rule with less erratic weight modification would produce a shorter step trajectory relative to the final trajectory. This property of less erratic weight modification is also desirable for biological systems, because each weight modification costs metabolic energy.

▶ **b** | Comparison of the length of step and final trajectories between EBN, predictive coding network (PCN), and an ANN, trained with backpropagation. Note that the length of both trajectories depends on the learning rate. Thus, in panels b–c, we present the length of the step and final trajectory on y and x axis, respectively; each point is from a specific learning rate (represented by the size of the marker; the legend does not enumerate all sizes). In such plots, when the two learning rules produce roughly the same length of final trajectory (which could be from different learning rates), one can compare the length of their step trajectory.

▶ **c** | The same comparison as in panel c, but for another EBN, namely, *GeneRec*[15]. GeneRec describes learning in recurrent networks, and ANN with this architecture is not trained by standard backpropagation, but by a variant of backpropagation, called *Almeida-Pineda*[16–18].

Implementation details. We train the models to predict a target pattern from an input pattern (both randomly generated from $\mathcal{N}(0,1)$ and of 32 entries), and there are 32 pairs of them (32 data points). The structure of the networks is $32 \to 32 \to 32 \to 32$. The batch size is one, as biological systems update the weights after each experience. The training is conducted for 64 epochs (one epoch iterates over all 32 data points). At the end of each epoch, current weights of the network are recorded as one set. Thus, it results in a sequence of 64 sets of weights. Each set of weights is used as one point to construct the step trajectory. The first and last sets of weights are used to construct the final trajectory. The length of the step and final trajectories can then be computed and reported in Supplementary Figs. 7b–c. For each combination of learning rule and learning rate, simulation is repeated $n = 20$ times with different seeds, and the error bars represent 68% confidence intervals.

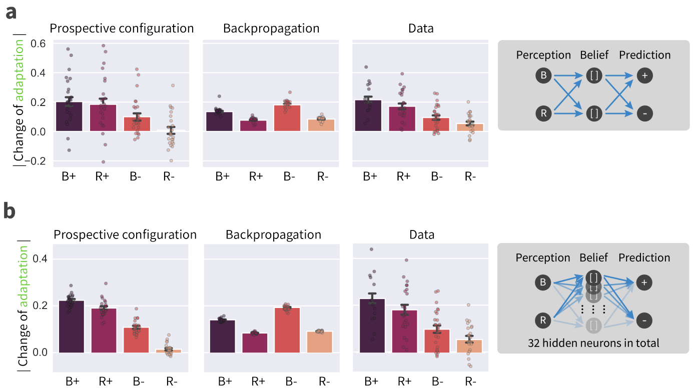

Supplementary Fig. 8

Motor learning experiment with fully-connected structure and more hidden neurons. In the experiments explaining biological observations, for simplicity, we simulated minimal networks necessary to perform these tasks, but it is important to establish if task structure can be discovered and learned by the networks without specifying network structure. Thus, here we repeat the motor learning experiment in Fig. 5 with general fully-connected structure (panel a) and 32 hidden neurons (panel b). Insets illustrate the structure of the networks. In both cases, prospective configuration is able to discover the task structure itself and reproduce the experimental observations; while backpropagation cannot. One should also notice that the variance of the simulation results (spread of changes in adaptation) decreases with increase of the network size.

Each experiment is repeated with $n = 24$ random seeds. Error bars and bands represent the 68% confidence interval.

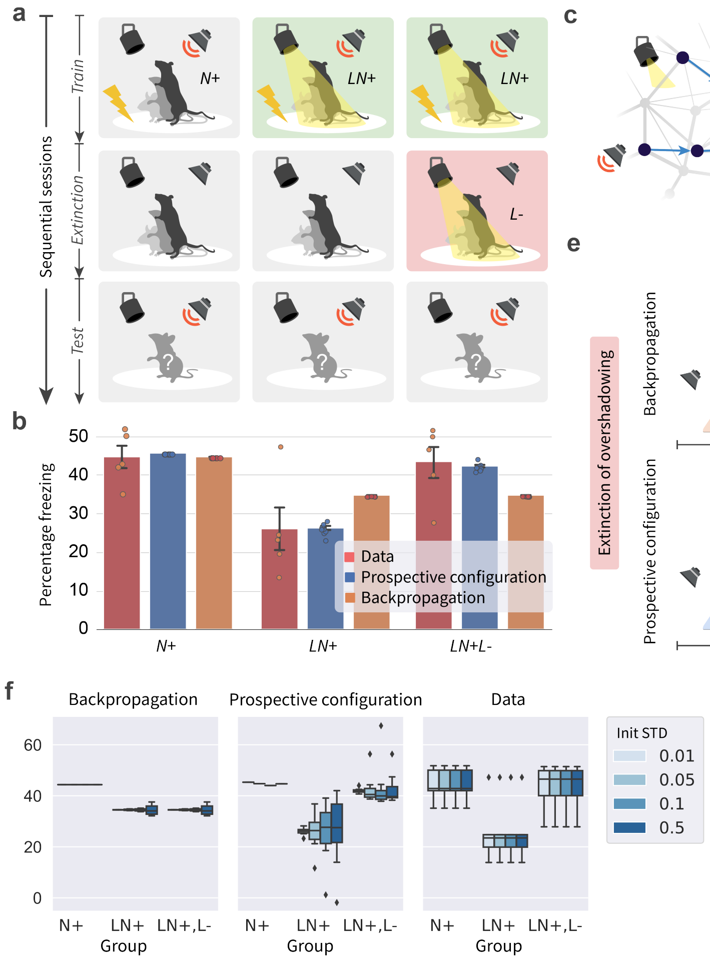

Supplementary Fig. 9

Prospective configuration explains extinction of overshadowing in fear conditioning. The extinction of overshadowing effect[19] can be accurately reproduced and explained by prospective configuration, but not backpropagation (comparing “Data” against “Prospective configuration” and “Backpropagation” in panel b).

▶ **a** | Experimental procedure. Rats were divided into three groups, corresponding to three columns. Each group underwent three sessions sequentially, corresponding to the top three rows, namely, train, extinction, and test. The goal of the training session was to associate fear (+) with different presented stimuli *N* or *LN* depending on the group: rats experienced an electric shock paired with different stimuli, where *N* and *L* stands for noise and light, respectively. Next, during the extinction session no shock was given, and for the third group the light was presented but without the shock, aiming to eliminate the fear (-) of light (*L*). Finally, all groups underwent a test session measuring how much fear was associated with the noise: the noise was presented and the percentage of freezing of rats was measured.

▶ **b** | Experimental and simulation results. The bar chart plots the percentage of freezing during test for each group, both measured in the animal experiments[19] (i.e., Data) and simulated by the two learning rules. Two effects are present in experimental data. First, comparing the groups *N*+ and *LN*+ demonstrates the overshadowing effect: there is less fear of noise if the noise had been compounded with light when paired with shock *LN*+ than if the noise alone had been paired with shock *N*+ (that is, light overshadows noise in a conditioned fear experiment). This effect can be accounted for by the canonical

model of error-driven learning — the Rescorla-Wagner model[20], and consequently it can be also produced by both error-driven models we consider — backpropagation and prospective configuration (explained in panel d). Second, comparing the groups *LN+* and *LN+L-* shows the striking effect of extinction of overshadowing: presenting the light without the shock increases the fear response to the non-presented stimulus — noise. This effect is not produced by backpropagation, but can be reproduced by prospective configuration (explained in panel e).

▶ **c** | The neural architecture considered: both stimuli are processed by hidden neurons (i.e., intermediate neurons corresponding to visual and auditory cortices) and are then combined to produce the prediction of electric shock (i.e., fear).

▶ **d** | Explanation of overshadowing effect, i.e., the reduced percentage freezing comparing group *LN+* against *N+*. With the energy machine introduced in Fig. 2, the diagram illustrates the state of the network after the *Train* sessions in groups *LN+* and *LN+L-*. The network learns to predict a shock (i.e. produces output of 1), on the basis of two stimuli, hence each of the inputs to the output neuron must be 0.5. Therefore, if only one stimulus is presented, the output of the network is reduced to 0.5. The network shown in this panel is acting as the starting point of learning in panel e.

▶ **e** | Explanation of extinction of overshadowing effect, i.e., the increased percentage freezing after noise in group *LN+L-* in comparison to *LN+*. This effect suggests that during extinction trials, where light is presented without a shock, the animals increased fear prediction to noise. As shown in this panel, backpropagation (top) cannot explain this, since the error cannot be backpropagated to and drive a weight modification on a non-activated branch where no stimuli are presented; prospective configuration (bottom), however, can account for this. Specifically, on the non-activated branch, the hidden neural activity decreases from zero to a small negative value (it may correspond to a neural activity decreasing below the baseline[21]). Since a weight modification depends on the product of the presynaptic activity and the postsynaptic activity representing the error, which are both negative here, the weight on the non-activated branch is strengthened.

▶ **f** | Robustness to different standard deviations of initial weights. We also simulated networks with different standard deviations of initial weights (ranging from 0.01 to 0.5, represented by the depth of the colour). It is shown that prospective configuration fits better to the data measured in the animal experiments than backpropagation, regardless of the standard deviation of initial weights. The box plots show the quartiles of the percentage of freezing, the line in the middle indicates the median, while the whiskers extend to show the rest of the distribution, except for the points that are determined to be outliers, which are shown separately.

Each experiment in panels b and f is repeated with $n = 8$ random seeds. Error bars and bands represent the 68% confidence interval.

Implementation details. As shown in panel c, the simulated network includes 2 input, 2 hidden, and 1 output neurons. The weights are initialized from a normal distribution of mean 0 and standard deviation 0.01, reflecting that the animals have not built an association between stimulus and electric shock before the experiments. Presenting or not presenting the stimulus (noise, light, or shock) is encoded as 1 and 0, respectively. The two input neurons are considered to be the visual and auditory neurons; thus, their activity corresponds to perceiving light and noise, respectively. The output neuron is considered to encode the prediction of the electric shock. The training and extinction sessions are both simulated for 32 iterations with the learning rate of 0.01. In the test session, the model makes a prediction with the presented stimulus (noise only). As in the Methods section, we denote by x_c the prediction for each group c from a set $C = \{N+, LN+, LN+L-\}$. To map the prediction to the percentage of freezing, it is scaled by a coefficient a (as the neural activity and the measure of freezing have different units) and shifted by

a bias b (as the rats may have some tendency to freeze after salient stimuli even if they had not been associated with a shock). The numbers reported in panel b are these scaled predictions. The coefficient a (constrained to be positive) and bias b are optimized for prospective configuration and backpropagation independently, analogously as described in the Methods section, i.e. their values that minimize summed squared error given below are found analytically.

$$\sum_{c \in C} (a x_c + b - d_c)^2 \tag{1}$$

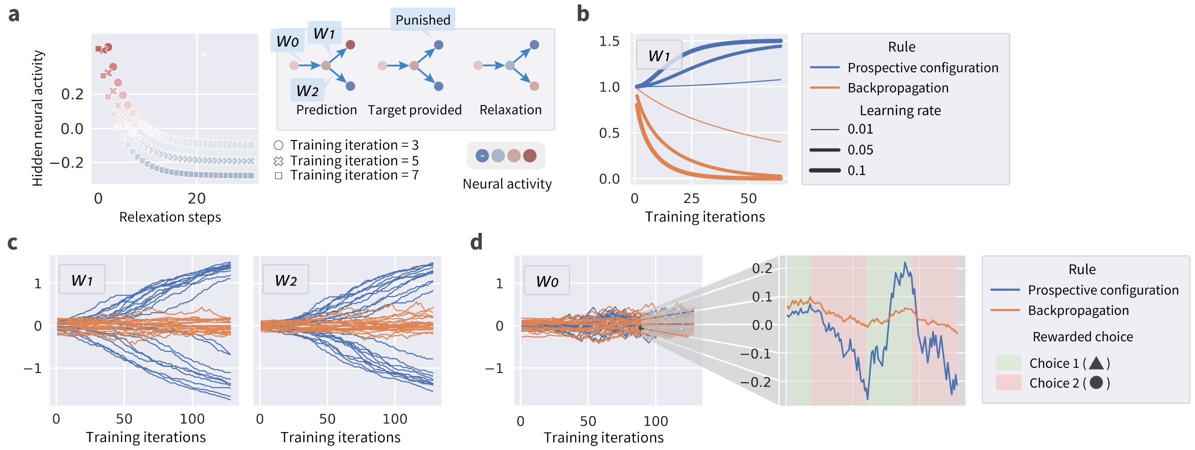

Supplementary Fig. 10

Inference of rewarded choice in the models of human reinforcement learning in Fig. 6. To shed light on the difference between prospective configuration and backpropagation in this task we first simulate an "idealized" version of the task, where the rewards and punishments are delivered deterministically, and the reversal only occurs once at the beginning of training (panels a and b), and then we show that insights from this idealized version translate to the full task from the human experiment (panels c and d).

▶ **a** | Here, we inspect prospective configuration at the first few training iterations: during relaxation, the hidden neuron is able to infer its prospective configuration, i.e., negative hidden activity encoding that the rewarded choice has reversed. The structure of the network is shown in the inset, it starts from ($\{W_0 = 1, W_1 = 1, W_2 = -1\}$) and is trained for 64 trials in total.

▶ **b** | Here, we show that such inference by prospective configuration results in an increase of W_1: since it has inferred from the punishment that the rewarded choice has reversed to a non-rewarded one, such punishment strengthens the connection from the latent state representing non-rewarded choice to a punishment. By contrast, in backpropagation W_1 is decreased: since it receives a punishment without updating the latent state (still encoding that the rewarded choice has not changed), it weakens the connection from the latent state to a reward.

▶ **c** | Here, we show that the W_1 and W_2 in the simulation of the full task with stochastic rewards. The weights follow a similar pattern as in the simplified task, i.e., their magnitude increases in prospective configuration. This signifies that the network learns that the rewards from the two options are jointly determined by a hidden state. This increase of the magnitude of W_1 and W_2 enables the network to infer the hidden state from the feedback, and learn the task structure (as described for panel b).

▶ **d** | Here, we show the evolution of W_0 in the full task. In prospective configuration, this weight remains closer to 0 than W_1 and W_2. Inset shows W_0 on one of the simulation in the main plot, where it is demonstrated that prospective configuration easily flips W_0 as the rewarded choice changes, while backpropagation has difficulty in accomplishing this. The reason of such behavior is as follows: thanks to large magnitude of W_1 and W_2 in prospective configuration, an error on the output unit results in a large error on the hidden unit, so the network is able to quickly flip the sign of W_0 whenever the observation mismatches the expectation. This results in an increased expectation on the Switch trials (Fig. 6c).

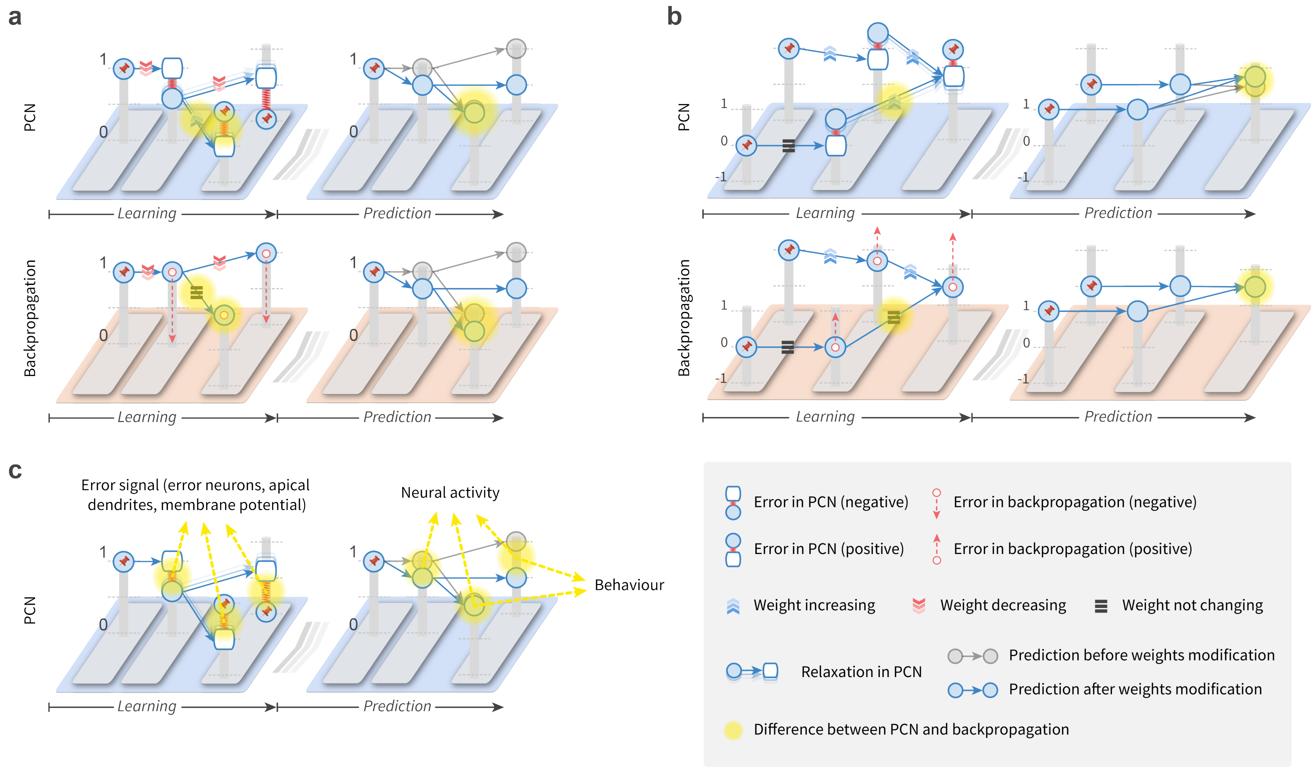

Supplementary Fig. 11

Experimental predictions of prospective configuration and backpropagation. To provide examples of experimental predictions of prospective configuration, panels a-b (and Supplementary Fig. 12) illustrate different behaviour of the learning rules in simple network motifs, which are minimal networks displaying given behaviour. Two motifs in this figure have been already analysed earlier in the paper, but there we focused on differences corresponding to experimentally observed effects, while in this figure we also add other qualitative differences that reveal a range of untested predictions of prospective configuration. Here, we consider a *predictive coding network*[7,8] (PCN) with the energy machine in Fig. 2, however, a similar analysis can be applied to other energy-based networks, which also follow the principle of prospective configuration. In each panel, the top and bottom rows demonstrate the prediction of PCNs and backpropagation, respectively. The left column adds the differences in the prediction errors during learning and the resulting weight update. The right column demonstrates the neuron activity before (transparent) and after (opaque) weight update. The differences between the rules are highlighted in yellow. Experimental predictions following from them can be derived as summarized in panel c.

▶ **a** | The error may spread to the branch where the prediction is correctly made. This motif has been compared with experimental data in Fig. 6, but here we focus on the effect illustrated in Fig. 1 and Fig. 2d, which despite being intuitive, has not been tested experimentally to our knowledge. The panel adds that an error on one output in PCN results in prediction error on the other, correctly predicted output. This produces an increase of the weight of the correct output neuron, which compensates for the decrease of the weight from the input, and enables the network to make correct prediction on the next trial.

▶ **b** | The error may cause a weight change in the sensory regions associated with absent stimuli. The panel shows a similar motif as the one investigated in Supplementary Fig. 9. The difference is that Supplementary Fig. 9 introduces negative error while this panel introduces positive error on the same architecture. Interestingly, introducing negative (Supplementary Fig. 9) or positive (this panel) error to the

same architecture produce a similar effect in the PCN, i.e. an increased predicted output for the stimulus not presented during learning.

▶ **c** | Observing model behaviour in experiments. The diagram summarizes how the differences illustrated in previous panels could be measured in experiments. The key difference in models' behaviour during learning is the difference in error signals. However, currently it is not clear how the prediction errors are represented in the cortical circuits. Three hypotheses have been proposed in the literature that errors are encoded in: activity of separate error neurons[2,7,22], membrane potential of value neurons[23,24], membrane potential in apical dendrites of value neurons[3,4]. Nevertheless, if the future research establishes how errors are encoded, it will be possible to test the predictions related to errors during learning. For example, one can design a task corresponding to panel a, where predictions in two modalities have to be made on the basis of a stimulus. One can then test if omission in one modality results in error signals in the brain region corresponding to the correctly predicted modality. The models also differ in the neural activity of the value nodes during the next trial following the learning. Such predictions are easier to test, because if the model makes a prediction without observing any supervised signal, then all errors are equal to 0 in PCNs, so the neural activity should reflect just the activity of value nodes. Additionally, the differences in the activity of the output value neurons should be testable in behavioural experiments. For example, panel b makes a behavioural prediction (presenting light with stronger shock should also increase freezing for tone) that can be tested in a similar way as described in Supplementary Fig. 9. Testing this prediction would also validate our explanation of the experimental result in Supplementary Fig. 9.

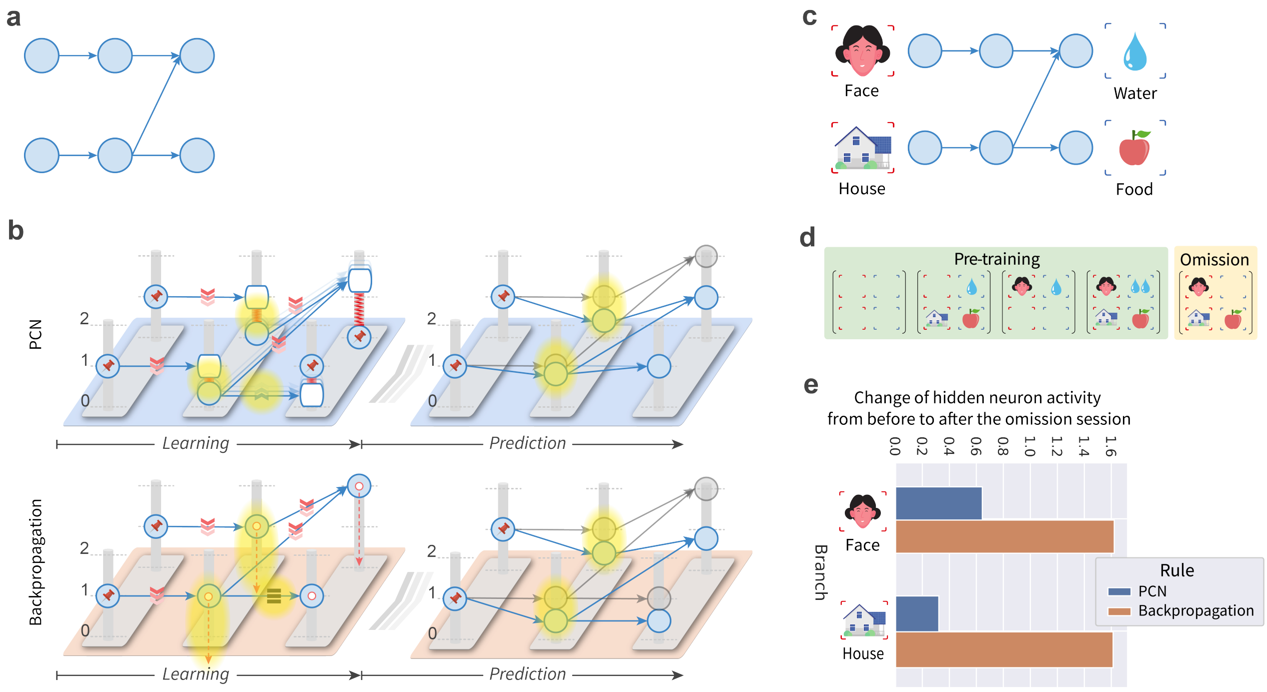

Supplementary Fig. 12

Experimental predictions concerning errors assigned to hidden nodes. The figure demonstrates a striking difference in how prospective configuration and backpropagation assign error to hidden nodes. Namely, in prospective configuration, the error assigned to a hidden node is reduced if the node is also connected to correctly predicted outputs. This difference is illustrated in a motif (panel a), for which we illustrate behaviour of learning rules with the energy machine (panel b), and describe a sample experiment testing model predictions (panels c–d) . Finally, we report the simulation results of the two learning rules (panel e), confirming that they indeed make distinct predictions for this motif.

▶ **a** | In this motif, two stimuli are presented and two predictions are made. One stimulus contributes to only one prediction, while the other stimulus contributes to both predictions.

▶ **b** | Comparison of learning rules' behaviour with the energy machine (notation as in Supplementary Fig. 11). The diagrams illustrate a network containing the motif (panel a), in a situations where one of the predicted outputs (top output) is omitted. A negative error is introduced to the prediction determined by both stimuli. Thus, we would expect the error to be assigned to hidden neurons on both branches. Both learning rules do so, however, they assign errors differently. PCNs allocate less error on the bottom hidden neuron than the top hidden neuron, because the bottom hidden neuron also contributes to another output that was correctly predicted, while backpropagation assigns the same error to both hidden neurons. This is also a nice example where prospective configuration (PCNs) demonstrates more intelligent behavior.

▶ **c** | Experimental stimuli. To test this motif, it is important to choose stimuli for which neural activity of "hidden" neurons can be easily measured. In case of a human experiment, inputs could contain faces and houses, because the hidden neurons would correspond to the brain regions known to be specifically excited by these particular types of stimuli, and the activity of these regions could be easily distinguished in an experiment[25]. The outputs could correspond to reward modalities (e.g. water and food). In case of a human experiment, these could be "virtual rewards" the participants are instructed to gather, while for animals, these could be the actual rewards.

▶ **d** | Experimental procedure. The motif shown in panel c could arise in brain networks from training

with examples shown in the green box. To test differences in behaviour of learning rules, partial omission trials could be presented, in which one of the expected outputs is omitted, as shown in the orange box.

► **e** | Results of simulations. We pre-train the models with the examples in the green box in panel d for a sufficient number of iterations until convergence, and then we train the model with the omission using the example in the orange box in panel d for one trial. We measure the change of hidden neural activity on both branches from before to after the above omission session. The graph shows simulation results of such change in hidden activity: PCNs predict different changes on different branches, while backpropagation predicts the same change on different branches (consistent with illustration in panel b, right).

Implementation details. Presenting and not presenting a stimulus (face, house, water, or food) are encoded as 1 and 0, respectively. Presenting two drops of water is encoded as 2. The network is initialized to the pre-trained connection pattern demonstrated in Supplementary Figs. 12c, i.e., the weights visible on the panel are set to one and other weights are set to zero. Such pattern of weights would arise from pre-training with the four examples in Supplementary Figs. 12d (in the green "Pre-training" box), but for simplicity, we do not simulate such pre-training but just set the weights as explained before. Next, to measure the activity of hidden units of such network during prediction, we set both inputs to 1 and record the hidden neural activity of the two branches. Subsequently, the model is presented with the omission trial shown in the orange box and the weights are updated once. Finally, to measure weight changes resulting from training on the subsequent prediction trial, we set both inputs to 1 and record the hidden neural activity of the two branches for the second time. The change of the hidden neuron activity from before to after the omission session can thus be computed for both branches.

2 Supplementary Notes

In this supplement, we present additional description and analysis of the simulated models. In Section 2.1, we provide details of all models simulated in the paper. In Section 2.2, we discuss relationship between prospective configuration and target propagation. In Section 2.3, we analyse prospective index of PCNs. In Section 2.4, we analyse target alignment of various learning models.

2.1 Details of simulated models

This section gives more details of all simulated models. The general idea of *energy-based networks* (EBNs) and *artificial neural networks* (ANNs), and one of EBNs, *predictive coding network*[7,8] (PCN), have been described in the Main article and Methods. PCN is again included here along with other simulated models to provide descriptions in a unified form, facilitating the reproduction of our reported results. Complete code and full documentation reproducing all simulation results written in Python is made publicly available at `https://github.com/YuhangSong/Prospective-Configuration`.

Algorithms 1 to 5 describe how the four models simulated in this paper predict and learn. These four models are: PCN, backpropagation, *GeneRec*[15], and *Almeida-Pineda*[16–18]. Among the four models, PCN and GeneRec are the two EBNs we investigate; backpropagation and Almeida-Pineda are the two ANNs we investigate. Specifically, PCN is compared against backpropagation, because it has been established that PCN are closely related to backpropagation[8,26] and they make the same prediction with the same weights and input pattern[8]. Therefore we simulated prediction in these two algorithms in the same way (Algorithm 1). However, they learn differently (c.f. Algorithm 2 and Algorithm 1 in the Methods of the main article). The other EBN, GeneRec, describes learning in recurrent networks, and ANN in this architecture is not trained by standard backpropagation, but a modified version proposed by Almeida and Pineda[16–18] (thus called the *Almeida-Pineda* algorithm). Thus, GeneRec should be compared against Almeida-Pineda because they make same prediction with the same weights and input pattern[15]. Therefore we simulated prediction in these two algorithms in the same way (Algorithm 3). But they learn differently (c.f. Algorithms 4 and 5). In a word, PCN and backpropagation are EBN and ANN working in feed-forward architecture, respectively; GeneRec and Almeida-Pineda are EBN and ANN working in recurrent architecture, respectively.

Algorithm 1: Predict with backpropagation or *predictive coding network*[7,8] (PCN)

Input: input pattern $\boldsymbol{s}^{\text{in}}$; synaptic weights $\{\boldsymbol{w}^1, \boldsymbol{w}^2, \cdots, \boldsymbol{w}^L\}$
Result: activity of output neurons $\boldsymbol{x}^{L+1}$

$\boldsymbol{x}^1 = \boldsymbol{s}^{\text{in}}$; // Clamp input neurons to input pattern
for $l = 1;\ l < L+1;\ l = l+1$ **do** // Forward pass of the network
  $\boldsymbol{x}^{l+1} = \boldsymbol{w}^l f\left(\boldsymbol{x}^l\right)$;
end

Particularly, PCN & Backpropagation work in a network where prediction is made from the input through a series of forward weights $\{\boldsymbol{w}^1, \boldsymbol{w}^2, \cdots, \boldsymbol{w}^L\}$; GeneRec & Almeida-Pineda works in a network where prediction is made from input through a mixture of forward weights $\{\boldsymbol{w}^1, \boldsymbol{w}^2, \cdots, \boldsymbol{w}^L\}$ and backward weights $\{\boldsymbol{m}^1, \boldsymbol{m}^2, \cdots, \boldsymbol{m}^L\}$. The forward weights $\{\boldsymbol{w}^1, \boldsymbol{w}^2, \cdots, \boldsymbol{w}^L\}$ and backward weights $\{\boldsymbol{m}^1, \boldsymbol{m}^2, \cdots, \boldsymbol{m}^L\}$ are not necessarily related. This architecture is also similar to the continuous Hopfield model[27,28]. Unlike in some previous studies[12], here, we focus on layered networks, where the sets of neurons at adjacent layers $\boldsymbol{x}^l$ and $\boldsymbol{x}^{l+1}$ are connected by synaptic weights. Thus, we define two sets of

weights for GeneRec & Almeida-Pineda that works in the recurrent network: $\boldsymbol{w}^l$ is the forward weights connecting from $\boldsymbol{x}^l$ to $\boldsymbol{x}^{l+1}$; $\boldsymbol{m}^l$ is the backward weights connecting from $\boldsymbol{x}^{l+1}$ to $\boldsymbol{x}^l$.

Algorithm 2: Learn with backpropagation

```
Input: input pattern s^in; target pattern s^target; synaptic weights {w^1, w^2, ..., w^L}
Output: updated synaptic weights {w^1, w^2, ..., w^L}
x^1 = s^in ;                                      // Clamp input neurons to input pattern
for l = 1; l < L+1; l = l+1 do                    // Forward pass of the network
    x^{l+1} = w^l f(x^l);
end
ε^{L+1} = s^target − x^{L+1} ;                    // Compute error of the output neurons
for l = L+1; l > 2; l = l−1 do                    // Backpropagation of error
    ε^{l−1} = f'(x^{l−1}) ∘ ((w^{l−1})^T ε^l);
end
for l = 1; l < L+1; l = l+1 do                    // Update weights
    Δw^l = α ε^{l+1} (f(x^l))^T;
    w^l = w^l + Δw^l;
end
```

Also note that GeneRec has been explored and re-discovered in recent works[29,30] showing how a closely related algorithm resembles backpropagation when the backward weights are the transposes of the forward weights $\boldsymbol{m}^l = \left(\boldsymbol{w}^l\right)^T$ (or for a fully-connected network in their context $w_{i,j} = w_{j,i}$), and how the extreme version of the algorithm approximate backpropagation[12].

Supplementary Fig. 5 additionally investigates *Strong Deep Feedback Control*[13,14] (strong-DFC). *Deep Feedback Control*[11] (DFC) was proposed to work with "infinitely weak nudging", as in equilibrium propagation[12]. More recent work demonstrates that it also works with "strong control"[13,14] (thus, called strong-DFC), i.e., with the natural form of EBNs. Thus, in this paper we investigate strong-DFC. In strong-DFC (or DFC in general), backward weights $\boldsymbol{m}^l$ do not connect from layer $l+1$ to layer l as in other models investigated in the paper. Instead, $\boldsymbol{m}^l$ connects from the output layer $L+1$ to layer l. We use the provided code in `https://github.com/mariacer/strong_dfc` to simulate strong-DFC. All hyper parameters are kept as is in the provided code. We remove the activation function of the last layer in the original implementation[11], to keep consistent with the rest of the models investigated in this paper, thus, providing a fair comparison. Derivation and motivation of the model can be found in the original paper[13,14].

Some common notations in the algorithms are: α is the learning rate for weights update; γ and $\mathscr{T}$ are the integration step and length of relaxation, respectively (specific to the two EBNs, PCN and GeneRec); $\boldsymbol{s}^{\text{in}}$ and $\boldsymbol{s}^{\text{target}}$ are the input and target patterns, respectively. For Almeida-Pineda, which requires additional iterative process to propagate error, β and $\mathscr{K}$ are the integration step and length of this iterative process, respectively. In our simulation, we use $\beta = 0.01$ and $\mathscr{K} = 1600$.

All simulated models work in mini-batch mode, that is to say, one iteration is to update the weights for one step on a mini-batch of data randomly sampled from the training set for classification tasks. The above sampling is without replacement, i.e., the same examples will not be sampled again before the completion of a epoch, which is when the entire training set has been sampled once. For example, considering a dataset of 1000 examples with a batch-size (number of examples in a mini-batch) of 10, then each iteration

would update weights for one step on 10 examples, and it will take 100 such iterations to complete one epoch. To implement the Algorithms 1 to 5 described below in mini-batch mode, one can simply add an extra-dimension, the size of which is batch-size, to all the neuron-specific vectors in the algorithms such as $\boldsymbol{x}^l$, $\boldsymbol{\varepsilon}^l$ and etc., and then reduce this dimension by summing over it when computing weight update $\Delta\boldsymbol{w}^l$ (and $\Delta\boldsymbol{m}^l$ if the model is GeneRec or Almeida-Pineda).

Note that learning with Almeida-Pineda involves relaxation of the model, i.e., updating neural activity, in lines 5-12 of Algorithm 4. However, its function is to make a prediction with current weights and input pattern so that the error on the output neurons can be computed (in the following line 13), similar as the function of "forward pass" in backpropagation in lines 2-4 of Algorithm 2. The neural activity in the Almeida-Pineda model is fixed during spreading of error, like in backpropagation. Thus, Almeida-Pineda is classified as an ANN rather than an EBN (which updates neural activity during spreading of error).

Algorithm 3: Predict with *Almeida-Pineda*[16–18] or *GeneRec*[15]

Input: input pattern $\boldsymbol{s}^{\text{in}}$; forward and backward synaptic weights $\{\boldsymbol{w}^1, \boldsymbol{w}^2, \cdots, \boldsymbol{w}^L\}$ and $\{\boldsymbol{m}^1, \boldsymbol{m}^2, \cdots, \boldsymbol{m}^L\}$

Result: activity of output neurons $\boldsymbol{x}^{L+1}$

$\boldsymbol{x}^1 = \boldsymbol{s}^{\text{in}}$; // Clamp input neurons to input pattern

for $l = 2;\ l < L+2;\ l = l+1$ **do** // Initialize $\boldsymbol{x}$

  $\boldsymbol{x}^l = \boldsymbol{0}$;

end

for $t = 0;\ t < \mathcal{T};\ t = t+1$ **do** // Relaxation

  for $l = 2;\ l < L+1;\ l = l+1$ **do**

    $\Delta\boldsymbol{x}^l = \gamma\left(-\boldsymbol{x}^l + \boldsymbol{m}^l f\left(\boldsymbol{x}^{l+1}\right) + \boldsymbol{w}^{l-1} f\left(\boldsymbol{x}^{l-1}\right)\right)$;

    $\boldsymbol{x}^l = \boldsymbol{x}^l + \Delta\boldsymbol{x}^l$;

  end

  $\Delta\boldsymbol{x}^{L+1} = \gamma\left(-\boldsymbol{x}^{L+1} + \boldsymbol{w}^L f\left(\boldsymbol{x}^L\right)\right)$;

  $\boldsymbol{x}^{L+1} = \boldsymbol{x}^{L+1} + \Delta\boldsymbol{x}^{L+1}$;

end

Algorithm 4: Learn with *Almeida-Pineda*[16–18]

Input: input pattern $\boldsymbol{s}^{\text{in}}$; target pattern $\boldsymbol{s}^{\text{target}}$; forward and backward synaptic weights $\{\boldsymbol{w}^1, \boldsymbol{w}^2, \cdots, \boldsymbol{w}^L\}$ and $\{\boldsymbol{m}^1, \boldsymbol{m}^2, \cdots, \boldsymbol{m}^L\}$

Output: updated forward and backward synaptic weights $\{\boldsymbol{w}^1, \boldsymbol{w}^2, \cdots, \boldsymbol{w}^L\}$ and $\{\boldsymbol{m}^1, \boldsymbol{m}^2, \cdots, \boldsymbol{m}^L\}$

$\boldsymbol{x}^1 = \boldsymbol{s}^{\text{in}}$; // Clamp input neurons to input pattern
for $l = 2;\ l < L+2;\ l = l+1$ **do** // Initialize $\boldsymbol{x}$
  $\boldsymbol{x}^l = \boldsymbol{0}$;
end
for $t = 0;\ t < \mathscr{T};\ t = t+1$ **do** // Relaxation
  for $l = 2;\ l < L+1;\ l = l+1$ **do**
    $\Delta\boldsymbol{x}^l = \gamma\left(-\boldsymbol{x}^l + \boldsymbol{m}^l f\left(\boldsymbol{x}^{l+1}\right) + \boldsymbol{w}^{l-1} f\left(\boldsymbol{x}^{l-1}\right)\right)$;
    $\boldsymbol{x}^l = \boldsymbol{x}^l + \Delta\boldsymbol{x}^l$;
  end
  $\Delta\boldsymbol{x}^{L+1} = \gamma\left(-\boldsymbol{x}^{L+1} + \boldsymbol{w}^L f\left(\boldsymbol{x}^L\right)\right)$;
  $\boldsymbol{x}^{L+1} = \boldsymbol{x}^{L+1} + \Delta\boldsymbol{x}^{L+1}$;
end
$\boldsymbol{\varepsilon}^{L+1} = \boldsymbol{s}^{\text{target}} - \boldsymbol{x}^{L+1}$; // Compute error of the output neurons
for $l = 1;\ l < L+1;\ l = l+1$ **do** // Initialize $\boldsymbol{\varepsilon}$
  $\boldsymbol{\varepsilon}^l = \boldsymbol{0}$;
end
for $t = 1;\ t < \mathscr{K}+1;\ t = t+1$ **do** // Backpropagation of error
  for $l = 2;\ l < L+1;\ l = l+1$ **do**
    $\Delta\boldsymbol{\varepsilon}^l = \beta\left(-\boldsymbol{\varepsilon}^l + f'\left(\boldsymbol{x}^l\right) \circ \left(\boldsymbol{m}^l \boldsymbol{\varepsilon}^{l+1}\right) + f'\left(\boldsymbol{x}^l\right) \circ \left(\boldsymbol{w}^{l-1} \boldsymbol{\varepsilon}^{l-1}\right)\right)$;
    $\boldsymbol{\varepsilon}^l = \boldsymbol{\varepsilon}^l + \Delta\boldsymbol{\varepsilon}^l$;
  end
  $\Delta\boldsymbol{\varepsilon}^1 = \beta\left(-\boldsymbol{\varepsilon}^1 + f'\left(\boldsymbol{x}^1\right) \circ \left(\boldsymbol{m}^1 \boldsymbol{\varepsilon}^2\right)\right)$;
  $\boldsymbol{\varepsilon}^1 = \boldsymbol{\varepsilon}^1 + \Delta\boldsymbol{\varepsilon}^1$;
end
for $l = 1;\ l < L+1;\ l = l+1$ **do** // Update weights
  $\Delta\boldsymbol{w}^l = \alpha \boldsymbol{\varepsilon}^{l+1} \left(f\left(\boldsymbol{x}^l\right)\right)^T$;
  $\boldsymbol{w}^l = \boldsymbol{w}^l + \Delta\boldsymbol{w}^l$;
  $\Delta\boldsymbol{m}^l = \alpha \boldsymbol{\varepsilon}^l \left(f\left(\boldsymbol{x}^{l+1}\right)\right)^T$;
  $\boldsymbol{m}^l = \boldsymbol{m}^l + \Delta\boldsymbol{m}^l$;
end

Algorithm 5: Learn with *GeneRec*[15]

Input: input pattern $\boldsymbol{s}^{\text{in}}$; target pattern $\boldsymbol{s}^{\text{target}}$; forward and backward synaptic weights $\{\boldsymbol{w}^1, \boldsymbol{w}^2, \cdots, \boldsymbol{w}^L\}$ and $\{\boldsymbol{m}^1, \boldsymbol{m}^2, \cdots, \boldsymbol{m}^L\}$

Output: updated forward and backward synaptic weights $\{\boldsymbol{w}^1, \boldsymbol{w}^2, \cdots, \boldsymbol{w}^L\}$ and $\{\boldsymbol{m}^1, \boldsymbol{m}^2, \cdots, \boldsymbol{m}^L\}$

$\boldsymbol{x}^1 = \boldsymbol{s}^{\text{in}}$; // Clamp input neurons to input pattern
for $l = 2;\ l < L+2;\ l = l+1$ **do** // Initialize $\boldsymbol{x}$
  $\boldsymbol{x}^l = \boldsymbol{0}$;
end
for $t = 0;\ t < \mathcal{T};\ t = t+1$ **do** // Relaxation
  for $l = 2;\ l < L+1;\ l = l+1$ **do**
    $\Delta \boldsymbol{x}^l = \gamma\left(-\boldsymbol{x}^l + \boldsymbol{m}^l f\left(\boldsymbol{x}^{l+1}\right) + \boldsymbol{w}^{l-1} f\left(\boldsymbol{x}^{l-1}\right)\right)$;
    $\boldsymbol{x}^l = \boldsymbol{x}^l + \Delta \boldsymbol{x}^l$;
  end
  $\Delta \boldsymbol{x}^{L+1} = \gamma\left(-\boldsymbol{x}^{L+1} + \boldsymbol{w}^L f\left(\boldsymbol{x}^L\right)\right)$;
  $\boldsymbol{x}^{L+1} = \boldsymbol{x}^{L+1} + \Delta \boldsymbol{x}^{L+1}$;
end
for $l = 1;\ l < L+1;\ l = l+1$ **do** // Update weights (negative phase)
  $\Delta \boldsymbol{w}^l = -\alpha f\left(\boldsymbol{x}^{l+1}\right)\left(f\left(\boldsymbol{x}^l\right)\right)^T$;
  $\boldsymbol{w}^l = \boldsymbol{w}^l + \Delta \boldsymbol{w}^l$;
  $\Delta \boldsymbol{m}^l = -\alpha f\left(\boldsymbol{x}^l\right)\left(f\left(\boldsymbol{x}^{l+1}\right)\right)^T$;
  $\boldsymbol{m}^l = \boldsymbol{m}^l + \Delta \boldsymbol{m}^l$;
end
$\boldsymbol{x}^{L+1} = \boldsymbol{s}^{\text{target}}$; // Clamp output neurons to target pattern
for $t = 0;\ t < \mathcal{T};\ t = t+1$ **do** // Relaxation
  for $l = 2;\ l < L+1;\ l = l+1$ **do**
    $\Delta \boldsymbol{x}^l = \gamma\left(-\boldsymbol{x}^l + \boldsymbol{m}^l f\left(\boldsymbol{x}^{l+1}\right) + \boldsymbol{w}^{l-1} f\left(\boldsymbol{x}^{l-1}\right)\right)$;
    $\boldsymbol{x}^l = \boldsymbol{x}^l + \Delta \boldsymbol{x}^l$;
  end
end
for $l = 1;\ l < L+1;\ l = l+1$ **do** // Update weights (positive phase)
  $\Delta \boldsymbol{w}^l = \alpha f\left(\boldsymbol{x}^{l+1}\right)\left(f\left(\boldsymbol{x}^l\right)\right)^T$;
  $\boldsymbol{w}^l = \boldsymbol{w}^l + \Delta \boldsymbol{w}^l$;
  $\Delta \boldsymbol{m}^l = \alpha f\left(\boldsymbol{x}^l\right)\left(f\left(\boldsymbol{x}^{l+1}\right)\right)^T$;
  $\boldsymbol{m}^l = \boldsymbol{m}^l + \Delta \boldsymbol{m}^l$;
end

2.2 Relationships of predictive coding networks to target propagation (Supplementary Fig. 3)

In Supplementary Fig. 3, we illustrate that prospective configuration, particularly, *predictive coding network*[7,8] (PCN), has close a relationship to target propagation[31]. In this section, we formally prove these observations.

Note that these relationships of PCNs to target propagation on one hand build interesting connections to existing work, on the other hand serve as a step in providing a mathematical explanation of the target alignment of PCNs, as discussed in the later Section 2.4.4.

2.2.1 Target propagation

Algorithm 6: Learn with target-propagation

Input: input pattern $\boldsymbol{s}^{\text{in}}$; target pattern $\boldsymbol{s}^{\text{target}}$; synaptic weights $\left\{\boldsymbol{w}^1, \boldsymbol{w}^2, \cdots, \boldsymbol{w}^L\right\}$
Output: updated synaptic weights $\left\{\boldsymbol{w}^1, \boldsymbol{w}^2, \cdots, \boldsymbol{w}^L\right\}$

$\boldsymbol{x}^1 = \boldsymbol{s}^{\text{in}}$; // Clamp input neurons to input pattern
for $l = 1;\ l < L+1;\ l = l+1$ **do** // Forward pass of the network
  $\boldsymbol{x}^{l+1} = \boldsymbol{w}^l f\left(\boldsymbol{x}^l\right)$;
end
$\tilde{\boldsymbol{x}}^{L+1} = \boldsymbol{s}^{\text{target}}$;
$\boldsymbol{\varepsilon}^{L+1} = \tilde{\boldsymbol{x}}^{L+1} - \boldsymbol{x}^{L+1}$;
for $l = L+1;\ l > 2;\ l = l-1$ **do** // Target-propagation
  $\tilde{\boldsymbol{x}}^{l-1} = f^{-1}\left(\left(\boldsymbol{w}^{l-1}\right)^{-1} \tilde{\boldsymbol{x}}^l\right)$;
  $\boldsymbol{\varepsilon}^{l-1} = \tilde{\boldsymbol{x}}^{l-1} - \boldsymbol{x}^{l-1}$;
end
for $l = 1;\ l < L+1;\ l = l+1$ **do** // Update weights
  $\Delta \boldsymbol{w}^l = \alpha \boldsymbol{\varepsilon}^{l+1} \left(f\left(\boldsymbol{x}^l\right)\right)^T$;
  $\boldsymbol{w}^l = \boldsymbol{w}^l + \Delta \boldsymbol{w}^l$;
end

We first briefly review target propagation. The key insight behind target propagation is that rather than updating weights based on a gradient of a loss function, one can instead attempt to explicitly compute what are the optimal activity for the neurons so that they can produce the desired target pattern, and then update the weights so as to nudge the current neural activity towards the optimal activity directly. We call these optimal activity *local target* since if the neurons take this activity, the network would produce the desired target pattern. Importantly, we can directly compute the local target in terms of the *inverses* of the weights and activation functions. Namely, suppose that we have a three-layer network with activation functions $f()$, weight matrices $\boldsymbol{w}^1, \boldsymbol{w}^2, \boldsymbol{w}^3$ and an input pattern $\boldsymbol{s}^{\text{in}}$. The output of this network is $\boldsymbol{x}^4 = \boldsymbol{w}^3 f\left(\boldsymbol{w}^2 f\left(\boldsymbol{w}^1 f\left(\boldsymbol{s}^{\text{in}}\right)\right)\right)$. Suppose instead that we do not want the network to output $\boldsymbol{x}^4$ for a given $\boldsymbol{s}^{\text{in}}$ but rather a given target pattern $\boldsymbol{s}^{\text{target}}$. Then, the activity at the first layer $\tilde{\boldsymbol{x}}^1$ that would produce this desired activity can be exactly computed by inverting[1] the network $\tilde{\boldsymbol{x}}^1 = f^{-1}\left(\left(\boldsymbol{w}^1\right)^{-1} f^{-1}\left(\left(\boldsymbol{w}^2\right)^{-1} f^{-1}\left(\left(\boldsymbol{w}^3\right)^{-1} \boldsymbol{s}^{\text{target}}\right)\right)\right)$. From this, we can define a recursion of one local target in terms of another at the layer above,

$$\begin{aligned} \tilde{\boldsymbol{x}}^l &= f^{-1}\left(\left(\boldsymbol{w}^l\right)^{-1} \tilde{\boldsymbol{x}}^{l+1}\right) \\ \tilde{\boldsymbol{x}}^{L+1} &= \boldsymbol{s}^{\text{target}} \end{aligned} \tag{2}$$

Based on these targets we can define the errors in target propagation as $\boldsymbol{\varepsilon}^l = \tilde{\boldsymbol{x}}^l - \boldsymbol{x}^l$. These errors drive the update of weights according to:

$$\Delta \boldsymbol{w}^l = \alpha \boldsymbol{\varepsilon}^{l+1} \left(f\left(\boldsymbol{x}^l\right)\right)^T \tag{3}$$

[1]Note that in realistic networks the weight matrices are not all square so an exact inverse $\left(\boldsymbol{w}^l\right)^{-1}$ does not exist. Instead, we can compute approximations of the inverse using the Moore-Penrose pseudoinverse[32] $\left(\boldsymbol{w}^l\right)^{\dagger}$, which is the least squares solution to the optimization problem $\arg\min_{\boldsymbol{w}} \left\|\boldsymbol{I} - \boldsymbol{w}^l \boldsymbol{w}\right\|$.

This algorithm is summarized in Algorithm 6.

2.2.2 Analyses of the relationships

Now we formally prove the below observations in Supplementary Fig. 3 about how prospective configuration, particularly PCN, has close a relationship to target propagation[5]. In other words, we formally prove that

- In an output-constrained PCN, neural activity after relaxation converges to the local target;
- In an input-output-constrained PCN, neural activity after relaxation approaches to the weighted sum of the predicting activity and the local target.

In the above, predicting activity refer to the neural activity when the model is making prediction, and they are the same for both backpropagation and PCN as they compute the same neural activity when making a prediction.

Output-constrained PCN As mentioned, we first investigate the "output-constrained PCN": in this PCN input neurons are not clamped to the input pattern but output neurons are clamped to the target pattern. We show that in this PCN, the activity after relaxation is precisely equal to the local target. Since $\boldsymbol{x}^1$ is not constrained to the input pattern, we can look at its dynamic by setting $l = 1$ in Eq. 12 in the Methods of the main article. Since there is no error term or error nodes at the input layer, there is only the later term left when setting $l = 1$ in Eq. 12 in the Methods of the main article (note that here we write in matrix & vector form):

$$\Delta \boldsymbol{x}^1 = \gamma f'\left(\boldsymbol{x}^1\right) \circ \left(\left(\boldsymbol{w}^1\right)^T \boldsymbol{\varepsilon}^2\right) \tag{4}$$

$$= \gamma f'\left(\boldsymbol{x}^1\right) \circ \left(\left(\boldsymbol{w}^1\right)^T \left(\boldsymbol{x}^2 - \boldsymbol{w}^1 f\left(\boldsymbol{x}^1\right)\right)\right) \tag{5}$$

Considering the above dynamic has converged, we can set $\Delta \boldsymbol{x}^1 = \boldsymbol{0}$ in the above equation and solving for $\boldsymbol{x}^1$, then we can obtain the converged value of $\boldsymbol{x}^1$:

$$\boldsymbol{x}^1 = f^{-1}\left(\left(\boldsymbol{w}^1\right)^{-1} \boldsymbol{x}^2\right) \tag{6}$$

Now we look at the dynamic of $\boldsymbol{x}^2$ by setting $l = 2$ in Eq. 12 in the Methods of the main article:

$$\Delta \boldsymbol{x}^2 = \gamma \left(-\boldsymbol{\varepsilon}^2 + f'\left(\boldsymbol{x}^2\right) \circ \left(\left(\boldsymbol{w}^2\right)^T \boldsymbol{\varepsilon}^3\right)\right) \tag{7}$$

$$= \gamma \left(-\left(\boldsymbol{x}^2 - \boldsymbol{w}^1 f\left(\boldsymbol{x}^1\right)\right) + f'\left(\boldsymbol{x}^2\right) \circ \left(\left(\boldsymbol{w}^2\right)^T \left(\boldsymbol{x}^3 - \boldsymbol{w}^2 f\left(\boldsymbol{x}^2\right)\right)\right)\right) \tag{8}$$

Putting the solved $\boldsymbol{x}^1$, i.e., Eq. (6), into the above Eq., we have:

$$\Delta \boldsymbol{x}^2 = \gamma f'\left(\boldsymbol{x}^2\right) \circ \left(\left(\boldsymbol{w}^2\right)^T \left(\boldsymbol{x}^3 - \boldsymbol{w}^2 f\left(\boldsymbol{x}^2\right)\right)\right) \tag{9}$$

Considering the above dynamic has converged, we can set $\Delta \boldsymbol{x}^2 = \boldsymbol{0}$ in the above equation and solving for $\boldsymbol{x}^2$, then we can obtain the converged value of $\boldsymbol{x}^2$:

$$\boldsymbol{x}^2 = f^{-1}\left(\left(\boldsymbol{w}^2\right)^{-1} \boldsymbol{x}^3\right) \tag{10}$$

One can now see the proof goes recursively until $l = L$ and $\boldsymbol{x}^{L+1}$ is fixed to the target pattern $\boldsymbol{s}^{\text{target}}$:

$$\begin{aligned}\boldsymbol{x}^l &= f^{-1}\left(\left(\boldsymbol{w}^l\right)^{-1}\boldsymbol{x}^{l+1}\right)\\ \boldsymbol{x}^{L+1} &= \boldsymbol{s}^{\text{target}}\end{aligned} \tag{11}$$

which is exactly the recursive formula of the local target in target propagation, i.e., Eq. (2). Thus, neural activity of output-constrained PCN after relaxation equals to the local target.

Input-output-constrained PCN Secondly, we investigate the "input-output-constrained PCN": in this PCN both input and output neurons are clamped to the input and target patterns, respectively. We show that in this PCN, the activity after relaxation are the weighted sum of the predicting activity and the local target. Particularly, since in an input-output-constrained PCN we can only solve for the equilibrium after relaxation analytically in the linear case, we prove this for a linear PCN. Nevertheless, the analysis still provides useful insights. Looking at the network dynamics at a given layer l, i.e., Eq. 12 in the Methods of the main article, we can write the dynamics in the linear case as,

$$\Delta\boldsymbol{x}^l = \gamma\left(-\left(\boldsymbol{x}^l - \boldsymbol{w}^{l-1}\boldsymbol{x}^{l-1}\right) + \left(\boldsymbol{w}^l\right)^T\left(\boldsymbol{x}^{l+1} - \boldsymbol{w}^l\boldsymbol{x}^l\right)\right) \tag{12}$$

If we then set $\Delta\boldsymbol{x}^l = \mathbf{0}$ and solve for $\boldsymbol{x}^l$, we obtain,

$$\Delta\boldsymbol{x}^l = \mathbf{0} \implies -\left(\boldsymbol{x}^l - \boldsymbol{w}^{l-1}\boldsymbol{x}^{l-1}\right) + \left(\boldsymbol{w}^l\right)^T\left(\boldsymbol{x}^{l+1} - \boldsymbol{w}^l\boldsymbol{x}^l\right) = \mathbf{0} \tag{13}$$

$$\implies -\boldsymbol{x}^l + \boldsymbol{w}^{l-1}\boldsymbol{x}^{l-1} + \left(\boldsymbol{w}^l\right)^T\boldsymbol{x}^{l+1} - \left(\boldsymbol{w}^l\right)^T\boldsymbol{w}^l\boldsymbol{x}^l = \mathbf{0} \tag{14}$$

$$\implies \boldsymbol{x}^l + \left(\boldsymbol{w}^l\right)^T\boldsymbol{w}^l\boldsymbol{x}^l = \boldsymbol{w}^{l-1}\boldsymbol{x}^{l-1} + \left(\boldsymbol{w}^l\right)^T\boldsymbol{x}^{l+1} \tag{15}$$

$$\implies \left(\boldsymbol{I} + \left(\boldsymbol{w}^l\right)^T\boldsymbol{w}^l\right)\boldsymbol{x}^l = \boldsymbol{w}^{l-1}\boldsymbol{x}^{l-1} + \left(\boldsymbol{w}^l\right)^T\boldsymbol{x}^{l+1} \tag{16}$$

$$\implies \boldsymbol{x}^l = \left(\boldsymbol{I} + \left(\boldsymbol{w}^l\right)^T\boldsymbol{w}^l\right)^{-1}\left(\boldsymbol{w}^{l-1}\boldsymbol{x}^{l-1} + \left(\boldsymbol{w}^l\right)^T\boldsymbol{x}^{l+1}\right) \tag{17}$$

If we assume that the norm of the weights is large compared to the identity matrix $\boldsymbol{I}$, i.e., we consider $\left(\boldsymbol{I} + \left(\boldsymbol{w}^l\right)^T\boldsymbol{w}^l\right)^{-1} \approx \left(\left(\boldsymbol{w}^l\right)^T\boldsymbol{w}^l\right)^{-1}$, the above equilibrium solution can further be approximated by:

$$\implies \boldsymbol{x}^l \approx \left(\left(\boldsymbol{w}^l\right)^T\boldsymbol{w}^l\right)^{-1}\left(\boldsymbol{w}^{l-1}\boldsymbol{x}^{l-1} + \left(\boldsymbol{w}^l\right)^T\boldsymbol{x}^{l+1}\right) \tag{18}$$

$$\implies \boldsymbol{x}^l \approx \underbrace{\left(\left(\boldsymbol{w}^l\right)^T\boldsymbol{w}^l\right)^{-1}}_{\text{constant}}\quad\underbrace{\boldsymbol{w}^{l-1}\boldsymbol{x}^{l-1}}_{\substack{\text{predicting activity}\\ \text{for backpropagation and PCN}}}\quad+\quad\underbrace{\left(\boldsymbol{w}^l\right)^{-1}\boldsymbol{x}^{l+1}}_{\substack{\text{local target}\\ \text{from target propagation}}} \tag{19}$$

where the equilibrium solution is simply the weighted sum of the predicting activity and the local target.

In summary, during relaxation the activity in PCNs tends to move from the predicting activity towards the local target that would be computed by target propagation. These relationships on one hand build interesting connections to existing work, on the other hand serve as a step in providing a mathematical explanation of the target alignment of PCNs, as discussed in the later Section 2.4.4.

2.3 Prospective index of predictive coding networks (Supplementary Fig. 5)

This section formally proves two properties of the prospective index ϕ^l of a *predictive coding network*[7,8] (PCN), that can be observed in Supplementary Fig. 5d. To briefly recap, prospective index ϕ^l quantifies to what extent the hidden neural activity of the network following clamping output neurons to a target pattern is shifting toward the hidden neural activity following subsequent weight modification. Below we show two properties visible in Supplementary Fig. 5d:

- Firstly, prospective index of the first hidden layer (ϕ^2) in a PCN is always one.
- Secondly, the prospective index in other layer is close to one because, the weights $\boldsymbol{W}$ in PCN are updated towards a configuration $\boldsymbol{W}^*$ whose prospective index is one.

Note that these observations of high prospective index of PCNs on one hand formally define what we proposed as "prospective configuration" and distinguishes itself from backpropagation, on the other hand serve as a step in providing a mathematical explanation of the target alignment of PCNs, as discussed in the later Section 2.4.4.

2.3.1 Prospective index of the first hidden layer of PCN is always one

We assume that the model does not make a perfect prediction with the current weights, so that the error in the prediction drives the learning. As defined in Supplementary Fig. 5a, vectors $\boldsymbol{v}^{\oplus,l}$ and $\boldsymbol{v}^{\prime,l}$ describe the changes in hidden neuron activity, due to target pattern being provided and learning respectively. Specifically for layer $l=2$, these vectors are:

$$\boldsymbol{v}^{\oplus,2} = \boldsymbol{x}_{\boldsymbol{W}}^{\oplus,2} - \boldsymbol{x}_{\boldsymbol{W}}^{\ominus,2} \tag{20}$$

$$\boldsymbol{v}^{\prime,2} = \boldsymbol{x}_{\boldsymbol{W}^\prime}^{\ominus,2} - \boldsymbol{x}_{\boldsymbol{W}}^{\ominus,2} \tag{21}$$

We will now show that for PCN the above vectors $\boldsymbol{v}^{\oplus,2}$ and $\boldsymbol{v}^{\prime,2}$ point in the same direction. The change in activity due to learning $\boldsymbol{v}^{\prime,2}$ is equal to

$$\boldsymbol{v}^{\prime,2} = \boldsymbol{w}^{\prime,1} f\left(\boldsymbol{x}_{\boldsymbol{W}^\prime}^{\ominus,1}\right) - \boldsymbol{w}^{1} f\left(\boldsymbol{x}_{\boldsymbol{W}}^{\ominus,1}\right) \tag{22}$$

Since the value nodes of the first (input) layer $\boldsymbol{x}^1$ are always fixed to the input signal $\boldsymbol{s}^{\text{in}}$, the above Eq. (22) can further be written as,

$$\begin{aligned}\boldsymbol{v}^{\prime,2} &= \boldsymbol{w}^{\prime,1} f\left(\boldsymbol{s}^{\text{in}}\right) - \boldsymbol{w}^{1} f\left(\boldsymbol{s}^{\text{in}}\right)\\ &= \left(\boldsymbol{w}^{\prime,1} - \boldsymbol{w}^{1}\right) f\left(\boldsymbol{s}^{\text{in}}\right)\\ &= \Delta \boldsymbol{w}^{1} f\left(\boldsymbol{s}^{\text{in}}\right)\end{aligned} \tag{23}$$

Using Eqs. 13 and 11 in the Methods of the main article, we write

$$\boldsymbol{v}^{\prime,2} = \alpha\left(\boldsymbol{x}_{\boldsymbol{W}}^{\oplus,2} - \hat{\boldsymbol{x}}_{\boldsymbol{W}}^{\oplus,2}\right)\left(f\left(\boldsymbol{s}^{\text{in}}\right)\right)^T f\left(\boldsymbol{s}^{\text{in}}\right) \tag{24}$$

In Eq. (24), $\hat{\boldsymbol{x}}^l$ denotes inputs to neurons in layer l, i.e., $\hat{\boldsymbol{x}}^l = \boldsymbol{w}^{l-1} f\left(\boldsymbol{x}^{l-1}\right)$. Note that $\hat{\boldsymbol{x}}_{\boldsymbol{W}}^{\oplus,2} = \boldsymbol{x}_{\boldsymbol{W}}^{\ominus,2}$, because both of these quantities are equal to $\boldsymbol{w}^1 f\left(\boldsymbol{s}^{\text{in}}\right)$ (the input of the first hidden layer ($l=2$) does not change

in response to output neuron being clamped). Using $\hat{\boldsymbol{x}}_{\boldsymbol{W}}^{\oplus,2} = \boldsymbol{x}_{\boldsymbol{W}}^{\ominus,2}$, the above Eq. (24) can further be written as,

$$\boldsymbol{v}'^{,2} = \left(\boldsymbol{x}_{\boldsymbol{W}}^{\oplus,2} - \boldsymbol{x}_{\boldsymbol{W}}^{\ominus,2}\right) \alpha \left(f\left(\boldsymbol{s}^{\text{in}}\right)\right)^{T} f\left(\boldsymbol{s}^{\text{in}}\right) \tag{25}$$

Note that $\alpha\left(f\left(\boldsymbol{s}^{\text{in}}\right)\right)^{T} f\left(\boldsymbol{s}^{\text{in}}\right)$ is a positive scalar (if at least one entry in the input pattern is non-zero). Comparing Eqs. (20) and (25), we can see that vectors $\boldsymbol{v}'^{,2}$ and $\boldsymbol{v}^{\oplus,2}$ are just scaled versions of each other, hence the cos of the angle between them is equal to 1, and thus prospective index is also equal to 1 (in the limit of $\kappa \to 0$).

2.3.2 Weights in PCN are updated towards a configuration with prospective index of one

As seen in Supplementary Fig. 5d, the prospective index for layers $l > 2$ is very close to one. To provide an intuition for why this is the case, in this section we demonstrate how PCNs would need to be modified to have prospective index equal to 1. We will refer to such modified model as target-PCN, and calculate its prospective index.

As in the previous section, we assume that the model does not make a perfect prediction with the current weights, so that the error in the prediction drives the learning. We start with recapping what happens in sequence in one iteration of the standard PCN.

1. Start from relaxation with only input neurons clamped to input pattern ($\ominus$) and with current weight $\boldsymbol{W}$, the hidden neuron activity settles to: $\boldsymbol{x}_{\boldsymbol{W}}^{\ominus,l}$
2. Both input and output neurons are clamped to the input and target pattern respectively ($\oplus$) and then the hidden neuron activity is relaxed to: $\boldsymbol{x}_{\boldsymbol{W}}^{\oplus,l}$
3. Weights $\boldsymbol{W}$ are updated for one step to $\boldsymbol{W}'$ to decrease the energy, while hidden neuron activity stays still from the last step: $\boldsymbol{x}_{\boldsymbol{W}}^{\oplus,l}$
4. Output neurons are freed but the input neuron is still clamped to the input pattern and then the hidden neuron activity is relaxed to: $\boldsymbol{x}_{\boldsymbol{W}'}^{\ominus,l}$

In the above step 3, weights are updated for one step from $\boldsymbol{W}$ to $\boldsymbol{W}'$. However, one can investigate the case of updating weights $\boldsymbol{W}$ for many steps until convergence $\boldsymbol{W}^*$ in the above step 3. This will result in weights $\boldsymbol{W}^*$ that represents: "the target towards which the weights $\boldsymbol{W}$ are updated". Thus, we call this variant "target-PCN" and it is summarized in Algorithm 7. Specifically, the procedure of target-PCN is to replace the above steps 3 and 4 of standard PCN with:

3. Weights are updated for many steps from $\boldsymbol{W}$ to $\boldsymbol{W}^*$ to decrease the energy till convergence, while hidden neuron activity stays still from the last step: $\boldsymbol{x}_{\boldsymbol{W}}^{\oplus,l}$;
4. Output neurons are freed but the input neuron is still clamped to the input pattern and then the hidden neuron activity is relaxed to: $\boldsymbol{x}_{\boldsymbol{W}^*}^{\ominus,l}$;

Algorithm 7: Learn with target-PCN

Input: input pattern $\boldsymbol{s}^{\text{in}}$; target pattern $\boldsymbol{s}^{\text{target}}$; synaptic weights $\{\boldsymbol{w}^1, \boldsymbol{w}^2, \cdots, \boldsymbol{w}^L\}$
Output: updated synaptic weights $\{\boldsymbol{w}^1, \boldsymbol{w}^2, \cdots, \boldsymbol{w}^L\}$

$\boldsymbol{x}^1 = \boldsymbol{s}^{\text{in}}$; // Clamp input neurons to input pattern
$\boldsymbol{x}^{L+1} = \boldsymbol{s}^{\text{target}}$; // Clamp output neurons to target pattern
for $t = 0$; $t < \mathscr{T}$; $t = t+1$ **do** // Relaxation
    for $l = 1$; $l < L+1$; $l = l+1$ **do**
        $\hat{\boldsymbol{x}}^{l+1} = \boldsymbol{w}^l f(\boldsymbol{x}^l)$;
        $\boldsymbol{\varepsilon}^{l+1} = \boldsymbol{x}^{l+1} - \hat{\boldsymbol{x}}^{l+1}$;
    end
    for $l = 2$; $l < L+1$; $l = l+1$ **do**
        $\Delta \boldsymbol{x}^l = \gamma\left(-\boldsymbol{\varepsilon}^l + f'(\boldsymbol{x}^l) \circ \left((\boldsymbol{w}^l)^T \boldsymbol{\varepsilon}^{l+1}\right)\right)$;
        $\boldsymbol{x}^l = \boldsymbol{x}^l + \Delta \boldsymbol{x}^l$;
    end
end
while $\{\boldsymbol{w}^1, \boldsymbol{w}^2, \cdots, \boldsymbol{w}^L\}$ *not converged* **do** // Update weights till convergence
    for $l = 1$; $l < L+1$; $l = l+1$ **do**
        $\hat{\boldsymbol{x}}^{l+1} = \boldsymbol{w}^l f(\boldsymbol{x}^l)$;
        $\boldsymbol{\varepsilon}^{l+1} = \boldsymbol{x}^{l+1} - \hat{\boldsymbol{x}}^{l+1}$;
    end
    for $l = 1$; $l < L+1$; $l = l+1$ **do** // Update weights
        $\Delta \boldsymbol{w}^l = \alpha \boldsymbol{\varepsilon}^{l+1} \left(f(\boldsymbol{x}^l)\right)^T$;
        $\boldsymbol{w}^l = \boldsymbol{w}^l + \Delta \boldsymbol{w}^l$;
    end
end

In the following, we demonstrate prospective index of target-PCN is one for all layers. First, we should notice that the minimum of energy E of PCN is zero, since the energy function is a sum of quadratic terms, i.e., Eq. 6 in the Methods of the main article. Then, we should notice that such energy E of PCN can be optimized to its minimum of zero by optimizing only $\boldsymbol{W}$. Particularly, the local energy term of layer l is:

$$\begin{aligned}\frac{1}{2}\left(\boldsymbol{\varepsilon}^l\right)^T \boldsymbol{\varepsilon}^l &= \frac{1}{2}\left(\boldsymbol{x}^l - \hat{\boldsymbol{x}}^l\right)^T \left(\boldsymbol{x}^l - \hat{\boldsymbol{x}}^l\right) \\ &= \frac{1}{2}\left(\boldsymbol{x}^l - \boldsymbol{w}^{l-1} f\left(\boldsymbol{x}^{l-1}\right)\right)^T \left(\boldsymbol{x}^l - \boldsymbol{w}^{l-1} f\left(\boldsymbol{x}^{l-1}\right)\right)\end{aligned} \tag{26}$$

In the above Eq., $\boldsymbol{x}^l - \boldsymbol{w}^{l-1} f\left(\boldsymbol{x}^{l-1}\right)$ can be optimized to produce a zero vector by optimizing only $\boldsymbol{w}^{l-1}$, as long as $f\left(\boldsymbol{x}^{l-1}\right)$ is not a zero vector. Specifically, let us denote all the non-zero entries in $f\left(\boldsymbol{x}^{l-1}\right)$ by $\left\{f\left(x_i^{l-1}\right)\right\}_{i\in I}$, where I is the set of indices i so that $f\left(x_i^{l-1}\right)$ is non-zero. Since $f\left(\boldsymbol{x}^{l-1}\right)$ is not a zero vector, $I \neq \emptyset$. To demonstrate that there exists a solution for $\left\{w_{j,i}^{l-1}\right\}_{i\in I}$ so that $x_j^l = \sum_{i\in I} w_{j,i}^{l-1} f\left(x_i^{l-1}\right)$, we construct an example of such solution. Such sample solution is to pick one index g from I, then have $w_{j,g}^{l-1} = \frac{x_j^l}{f\left(x_i^{l-1}\right)}$ and $\left\{w_{j,i}^{l-1} = 0 : i \in I, i \notin \{g\}\right\}$. Thus, as long as $f\left(\boldsymbol{x}^{l-1}\right)$ is not a zero vector ($I \neq \emptyset$), there exists a solution of $\boldsymbol{w}^{l-1}$ that makes $\boldsymbol{x}^l - \boldsymbol{w}^{l-1} f\left(\boldsymbol{x}^{l-1}\right)$ a zero vector.

Thus, in step 3 of the target-PCN, the energy of the network is at its minimum of zero. This further implies that in the step 4 of the target-PCN, the neural activity does not move, i.e.,

$$x^{\ominus,l}_{W^*} = x^{\oplus,l}_{W} \tag{27}$$

According to the definition of prospective index in Supplementary Figs. 5a-b, the prospective index of this target-PCN ($\phi^{*,l}$) is:

$$\begin{aligned}\phi^{*,l} &= \frac{v^{\oplus,l} \cdot v^{*,l}}{\left(\|v^{\oplus,l}\| + \kappa\right)\left(\|v^{*,l}\| + \kappa\right)} \\ &\approx cos\left(v^{\oplus,l}, v^{*,l}\right) \\ &= cos\left(\overrightarrow{x^{\ominus,l}_{W} x^{\oplus,l}_{W}}, \overrightarrow{x^{\ominus,l}_{W} x^{\ominus,l}_{W^*}}\right) \\ &= cos\left(\overrightarrow{x^{\ominus,l}_{W} x^{\oplus,l}_{W}}, \overrightarrow{x^{\ominus,l}_{W} x^{\oplus,l}_{W}}\right) \text{ according to Eq. (27)} \\ &= 1\end{aligned} \tag{28}$$

This theoretical result is further confirmed by empirical observation in Supplementary Fig. 5d. Since the standard PCN modifies the weights in a similar direction as target-PCN, it is likely to have a similar prospective index.

In summary, PCN has a high prospective index. This on one hand formally defines what we proposed as "prospective configuration" and distinguishes itself from backpropagation, on the other hand serve as a step in providing a mathematical explanation of the target alignment of PCNs, as discussed in the later Section 2.4.4.

2.4 Target alignment

In this section we provide a mathematical analysis of target alignment. First, we show that the target alignment is equal to 1 for various networks that do not include hidden layers. Next we demonstrate that target propagation produces target alignment of 1. The third subsections identifies a special condition under which backpropagation produces target alignment of 1. The last subsection addresses the question of why predictive coding networks (PCNs) have higher target alignment than backpropagation, using several findings in earlier sections.

2.4.1 Target alignment for networks without hidden layers (Fig. 3e)

Fig. 3e shows that target alignment for models without hidden layers, trained either with prospective configuration or backpropagation, is exactly one, and here we prove this property analytically. Without hidden layers, prospective configuration and backpropagation are identical algorithms. In a linear network, the change of the weight w^1 is:

$$\Delta w^1 = \alpha \varepsilon^2 \left(x^1\right)^T \tag{29}$$

We denote output after learning by x'^2. The change of the output $x'^2 - x^2$ is:

$$x'^2 - x^2 = w'^{,2} x^1 - w^2 x^1 \tag{30}$$

$$= \Delta w^1 x^1 \tag{31}$$

$$= \alpha \varepsilon^2 \left(x^1\right)^T x^1 \tag{32}$$

Here $\left(\boldsymbol{x}^1\right)^T \boldsymbol{x}^1$ is a positive scalar (if at least one entry in $\boldsymbol{x}^1$ is non-zero). Thus,

$$\boldsymbol{x}'^2 - \boldsymbol{x}^2 \sim \boldsymbol{\varepsilon}^2 \tag{33}$$

According to the definition of target alignment, which is the cosine similarity of the direction of the target (i.e., $\boldsymbol{\varepsilon}^2$) and the direction of learning (i.e., $\boldsymbol{x}'^2 - \boldsymbol{x}^2$), target alignment of this network is exactly one. This conclusion also applies to network with a nonlinear activation function.

2.4.2 Target alignment of target propagation (Supplementary Fig. 4a)

This subsection demonstrates that target alignment of target propagation is equal to 1. Such target alignment equal to 1 for target propagation is implied by Theorem 5 in the study of Meulemans et al.[6]. They show that if a network is linear and weights in each layer are invertible, then "parameter updates push the output activation along the negative gradient direction in the output space"[6]. Simulations in Supplementary Fig. 4a illustrate that the target alignment of target propagation is indeed equal to 1. For completeness we include in this paper a simple direct proof of this result (which we will also use in the next section).

For linear networks with invertible weights, the relationship between errors in adjacent layers in target propagation is:

$$\boldsymbol{\varepsilon}^l = \left(\boldsymbol{w}^l\right)^{-1} \boldsymbol{\varepsilon}^{l+1} \tag{34}$$

The activity of output neurons after the weight modification is:

$$\boldsymbol{x}'^{L+1} = \left(\boldsymbol{w}^L + \alpha \boldsymbol{\varepsilon}^{L+1} \left(\boldsymbol{x}^L\right)^T\right) \boldsymbol{w}'^{,L-1} \cdots \boldsymbol{w}'^{,1} \boldsymbol{x}^1 \tag{35}$$

$$= \boldsymbol{w}^L \boldsymbol{w}'^{,L-1} \cdots \boldsymbol{w}'^{,1} \boldsymbol{x}^1 + \boldsymbol{\varepsilon}^{L+1} \alpha \left(\boldsymbol{x}^L\right)^T \boldsymbol{w}'^{,L-1} \cdots \boldsymbol{w}'^{,1} \boldsymbol{x}^1 \tag{36}$$

Term $\alpha \left(\boldsymbol{x}^L\right)^T \boldsymbol{w}'^{,L-1} \cdots \boldsymbol{w}'^{,1} \boldsymbol{x}^1$ is a scalar, so let us denote it by c_L. Expanding $\boldsymbol{w}'^{,L-1}$ and using Eq. (34), we obtain:

$$\boldsymbol{x}'^{L+1} = \boldsymbol{w}^L \left(\boldsymbol{w}^{L_1} + \alpha \boldsymbol{\varepsilon}^L \left(\boldsymbol{x}^{L-1}\right)^T\right) \cdots \boldsymbol{w}'^{,1} \boldsymbol{x}^1 + c_L \boldsymbol{\varepsilon}^{L+1} \tag{37}$$

$$= \boldsymbol{w}^L \boldsymbol{w}^{L-1} \cdots \boldsymbol{w}'^{,1} \boldsymbol{x}^1 + \boldsymbol{w}^L \left(\boldsymbol{w}^L\right)^{-1} \boldsymbol{\varepsilon}^{L+1} \alpha \left(\boldsymbol{x}^{L-1}\right)^T \cdots \boldsymbol{w}'^{,1} \boldsymbol{x}^1 + c_L \boldsymbol{\varepsilon}^{L+1} \tag{38}$$

Note that $\boldsymbol{w}^L \left(\boldsymbol{w}^L\right)^{-1}$ is equal to the identity, so can be removed from the above equation, and $\alpha \left(\boldsymbol{x}^{L-1}\right)^T \cdots \boldsymbol{w}'^{,1} \boldsymbol{x}^1$ is a scalar, so denote it by c_{L-1}. Expanding all terms $\boldsymbol{w}'^{,l}$ analogously as above, we eventually obtain:

$$\boldsymbol{x}'^{L+1} = \boldsymbol{w}^L \cdots \boldsymbol{w}^1 \boldsymbol{x}^1 + (c_L + \cdots + c_1) \boldsymbol{\varepsilon}^{L+1} \tag{39}$$

Since the output before weight update was $\boldsymbol{w}^L \cdots \boldsymbol{w}^1 \boldsymbol{x}^1$, the change in the output is proportional to the direction towards target $\boldsymbol{\varepsilon}^{L+1}$, hence the target alignment is equal to 1. Given the similarity between target propagation and PCNs described in Section 2.2, the PCNs should also have target alignment relatively close to 1, as we will discuss further in Section 2.4.4.

It is also important to note that target propagation diverges from prospective configuration, when the feedback weights through which targets are propagated get small. This is implied by Eqs. (18) and (19) in Section 2.2, showing the relationship of predicting activity of backpropagation, local target of target propagation, and neural activities of PCN. Specifically, while deriving Eq. (19) (stating that neural activity in PCNs is a linear combination of predictive activity and local target from target propagation), an

assumption had to be made that the norm of the feedback weights $\boldsymbol{w}^l$ is large compared to the identity matrix $\boldsymbol{I}$. For example, if all feedback weights are equal to $\boldsymbol{w}^l_{i,j} = 0$, the neural activity $\boldsymbol{x}^l$ will be just equal to the predicting activity.

Since target propagation has a desirable property of perfect target alignment, one may ask if the brain can employ target propagation rather than prospective configuration as is main learning principle. However, energy-based networks have several advantages over target propagation both in terms of computational properties and relationship with experimental data. Since target propagation requires computation of multiple matrix inverses, it is numerically unstable, so for example in Supplementary Fig. 4a we only show the result for networks with up to 5 layers, because we were unable to perform target propagation in deeper networks due to numerical instabilities. PCNs offer a nice alternative which is related to target propagation, but is numerically stable (e.g., avoids propagating targets by infinitely large inverse matrices when a weight matrix is zero). Furthermore, target propagation does not modify the activity of the neurons during relaxation, so it does not follow prospective configuration. Consequently, in the case of the network in Fig. 1 target propagation would not compensate the weight to olfactory output, because such compensation relies on updating the activity of the hidden neuron. Theory reviewed in this section implies that target propagation only produces target alignment equal to 1 if the weights are invertable, but this is not the case in the network in Fig. 1, so target propagation would not produce unity target alignment for this problem. Moreover, target propagation would not be able to reproduce the patterns of behaviour and neural activity in Figs. 5, 6 and Supplementary Fig. 9, because reproducing these data relies on modifying activity of hidden neurons after feedback, and target propagation does not do it.

2.4.3 Target alignment for orthogonal initialization (Supplementary Fig. 4c)

This subsection identifies one special conditions under which backpropagation produces target alignment of 1. Specifically, simulations in Supplementary Fig. 4c show that target alignment is equal to 1 for backpropagation in linear networks, when the weights are initialized to orthogonal values $\left(\boldsymbol{w}^l\right)^T = \boldsymbol{w}^l$. This observation can be explained using results from the previous section: when weights are orthogonal, then $\left(\boldsymbol{w}^l\right)^T = \left(\boldsymbol{w}^l\right)^{-1}$, hence the relationship between errors in adjacent layers is the same as for target propagation (Eq. (34)). Consequently, the same argument can be applied to backpropagation on linear networks with orthogonal initialization to show that it has target alignment equal to 1.

2.4.4 Target alignment of predictive coding networks

The subsection addresses the question of why PCNs have higher target alignment than backpropagation, using several findings in earlier sections. Specifically, to justify why PCNs have high target alignment, we can combine 3 facts that we demonstrate in earlier sections, and summarize here:

1. Target alignment of target propagation is equal to 1. This is shown in Section 2.4.2.
2. When target pattern is provided to output neurons in PCNs, during relaxation the neural activity in hidden layers converges to values related to local targets in target propagation. This is shown in Section 2.2.
3. Weight modification in PCNs reinforces the pattern of activity to which it converged during relaxation. In other words, predicting activity changes as a result of weight modification in the direction of the equilibrium reached during relaxation. This is shown in Section 2.3.

According to fact 3, learning in PCNs reinforces the equilibrium activity, which, according to fact 2, is largely dependent on the local targets. Therefore, the changes in activity in hidden layers due to learning in

PCNs are similar to those in target propagation, and hence the changes in the output activity are also likely to be similar, and the two algorithms should also share a similarity in target alignment. According to fact 1, target propagation has target alignment of 1, so the PCNs should also share a similar target alignment.

References

[1] Donald Olding Hebb. *The organisation of behaviour: A neuropsychological theory*. Science Editions New York, 1949.

[2] Andre M Bastos et al. "Canonical microcircuits for predictive coding". In: *Neuron* 76.4 (2012), pp. 695–711.

[3] João Sacramento et al. "Dendritic cortical microcircuits approximate the backpropagation algorithm". In: *Advances in Neural Information Processing Systems (NeurIPS)*. 2018, pp. 8721–8732.

[4] James CR Whittington and Rafal Bogacz. "Theories of error back-propagation in the brain". In: *Trends in Cognitive Sciences* (2019).

[5] Dong-Hyun Lee et al. "Difference target propagation". In: *Joint European Conference on Machine Learning and Knowledge Discovery in Databases*. Springer. 2015, pp. 498–515.

[6] Alexander Meulemans et al. "A theoretical framework for target propagation". In: *Advances in Neural Information Processing Systems* 33 (2020), pp. 20024–20036.

[7] Rajesh PN Rao and Dana H Ballard. "Predictive coding in the visual cortex: A functional interpretation of some extra-classical receptive-field effects". In: *Nature Neuroscience* 2.1 (1999), pp. 79–87.

[8] James CR Whittington and Rafal Bogacz. "An approximation of the error backpropagation algorithm in a predictive coding network with local hebbian synaptic plasticity". In: *Neural Computation* 29.5 (2017), pp. 1229–1262.

[9] Xavier Glorot and Yoshua Bengio. "Understanding the difficulty of training deep feedforward neural networks". In: *Proceedings of the thirteenth international conference on artificial intelligence and statistics*. JMLR Workshop and Conference Proceedings. 2010, pp. 249–256.

[10] Andrew M Saxe, James L McClelland, and Surya Ganguli. "Exact solutions to the nonlinear dynamics of learning in deep linear neural networks". In: *arXiv preprint arXiv:1312.6120* (2013).

[11] Alexander Meulemans et al. "Credit assignment in neural networks through deep feedback control". In: *Advances in Neural Information Processing Systems* 34 (2021), pp. 4674–4687.

[12] Benjamin Scellier and Yoshua Bengio. "Equilibrium propagation: Bridging the gap between energy-based models and backpropagation". In: *Frontiers in Computational Neuroscience* 11 (2017), p. 24.

[13] Alexander Meulemans et al. "Minimizing control for credit assignment with strong feedback". In: *International Conference on Machine Learning*. PMLR. 2022, pp. 15458–15483.

[14] Alexander Meulemans et al. "The least-control principle for learning at equilibrium". In: *arXiv preprint arXiv:2207.01332* (2022).

[15] Randall C O'Reilly. "Biologically plausible error-driven learning using local activation differences: The generalized recirculation algorithm". In: *Neural Computation* 8.5 (1996), pp. 895–938.

[16] Luis B Almeida. "A learning rule for asynchronous perceptrons with feedback in a combinatorial environment". In: *Artificial Neural Networks: Concept Learning*. IEEE Computer Society Press, 1990, pp. 102–111.

[17] Fernando Pineda. "Generalization of back propagation to recurrent and higher order neural networks". In: *Advances in Neural Information Processing Systems (NeurIPS)*. 1987, pp. 602–611.

[18] Fernando J Pineda. "Dynamics and architecture for Neural Computation". In: *Journal of Complexity* 4.3 (1988), pp. 216–245.

[19] Mark A Kaufman and Robert C Bolles. "A nonassociative aspect of overshadowing". In: *Bulletin of the Psychonomic Society* 18.6 (1981), pp. 318–320.

[20] Robert A Rescorla. "A theory of Pavlovian conditioning: Variations in the effectiveness of reinforcement and nonreinforcement". In: *Current research and theory* (1972), pp. 64–99.

[21] Georg B Keller and Thomas D Mrsic-Flogel. "Predictive processing: A canonical cortical computation". In: *Neuron* 100.2 (2018), pp. 424–435.

[22] Alexander Attinger, Bo Wang, and Georg B Keller. "Visuomotor coupling shapes the functional development of mouse visual cortex". In: *Cell* 169.7 (2017), pp. 1291–1302.

[23] Martin Boerlin, Christian K Machens, and Sophie Denève. "Predictive coding of dynamical variables in balanced spiking networks". In: *PLoS Computational Biology* 9.11 (2013), e1003258.

[24] Wieland Brendel et al. "Learning to represent signals spike by spike". In: *PLoS Computational Biology* 16.3 (2020), e1007692.

[25] Hauke R Heekeren et al. "A general mechanism for perceptual decision-making in the human brain". In: *Nature* 431.7010 (2004), pp. 859–862.

[26] Yuhang Song et al. "Can the brain do backpropagation?—Exact implementation of backpropagation in predictive coding networks". In: *Advances in Neural Information Processing Systems (NeurIPS)*. Vol. 33. Europe PMC Funders, 2020, p. 22566.

[27] Dmitry Krotov and John J Hopfield. "Dense associative memory for pattern recognition". In: *Advances in Neural Information Processing Systems (NeurIPS)*. Vol. 29. 2016, pp. 1172–1180.

[28] Victor Soto, Alberto Suárez, and Gonzalo Martínez-Muñoz. "An urn model for majority voting in classification ensembles". In: *Advances in Neural Information Processing Systems (NeurIPS)*. Neural Information Processing Systems Foundation, 2016.

[29] Yoshua Bengio and Asja Fischer. "Early inference in energy-based models approximates back-propagation". In: *arXiv preprint arXiv:1510.02777* (2015).

[30] Yoshua Bengio et al. "STDP as presynaptic activity times rate of change of postsynaptic activity". In: *arXiv preprint arXiv:1509.05936* (2015).

[31] Yoshua Bengio. "How auto-encoders could provide credit assignment in deep networks via target propagation". In: *arXiv preprint arXiv:1407.7906* (2014).

[32] Roger Penrose. "A generalized inverse for matrices". In: *Mathematical proceedings of the Cambridge philosophical society*. Vol. 51. Cambridge University Press. 1955, pp. 406–413.
